# Diversity and dynamics of multiple symbionts contribute to early development of broadcast spawning reef-building coral *Dipsastraea veroni*

**DOI:** 10.1128/aem.02359-24

**Published:** 2025-01-29

**Authors:** Minglan Guo, Lei Jiang, Guowei Zhou, Jiansheng Lian, Xiaolei Yu, Hui Huang

**Affiliations:** 1 CAS Key Laboratory of Tropical Marine Bio-resources and Ecology, Guangdong Provincial Key Laboratory of Applied Marine Biology, South China Sea Institute of Oceanology, Chinese Academy of Sciences 74718 , Guangzhou, China; 2 CAS-HKUST Sanya Joint Laboratory of Marine Science Research, Key Laboratory of Tropical Marine Biotechnology of Hainan Province, Sanya Institute of Oceanology, SCSIO, Sanya, China; 3 Sanya National Marine Ecosystem Research Station, Tropical Marine Biological Research Station in Hainan, Chinese Academy of Sciences, Sanya, China; University of Delaware, Lewes, Delaware, USA

**Keywords:** sexual reproduction, scleractinian coral, *Dipsastraea veroni*, early life stage, horizontal acquisition, vertical transmission

## Abstract

Sexual reproduction and recruitment enhance the genetic diversity and evolution of reef-building corals for population recovery and coral reef conservation under climate change. However, new recruits are vulnerable to physical changes and the mechanisms of symbiosis establishment remain poorly understood. Here, *Dipsastraea veroni*, a broadcast spawning hermaphrodite reef-building coral, was subjected to settlement and juvenile growth in flow-through *in situ* seawater at 27.93 ± 0.96°C. Symbiosis of Symbiodiniaceae, bacteria, and/or archaea by horizontal acquisition and/or hypothetical vertical transmission through the mucus with symbionts from the parent appears to be a heritable process of selection and adaptation in *D. veroni* at the egg, larva, juvenile (5 days post settlement, d p.s. and 32 d p.s.) stages. Symbiodiniaceae was dominated by the genera *Cladocopium*, *Durusdinium*, *Symbiodinium,* with increasing relative abundance of *Durusdinium* at 5 d p.s. and *Symbiodinium* at 32 d p.s. Mixed acquisition of the dominant phyla Pseudomonadota, Bacteroidota, Cyanobacteriota, Bacillota, Planctomycetota, and Actinomycetota in egg, larva, and/or juvenile showed a winnowing and regulated bacterial diversity and dynamics, resulting in stage-abundant orders Pseudomonadales and Bacillales in egg and Rhodobacterales, Rhodospirillales, Cyanobacteria, and Cyanobacteriales in larva and/or juvenile. The photoautotrophic Chloroflexales, Cyanobacteriales, and Chlorobiales were abundant in adults. The stable archaeal community contained predominant Crenarchaeota, Halobacterota, Nanoarchaeia Thermoplasmatota, and eight rare phyla, with increased relative abundance of the genera *Bathyarchaeota*, *Candidatus_Nitrosopumilus*, *Candidatus_Nitrocosmicus*, *Nitrosarchaeum*, *Candidatus_Nitrosotenuis*, *Candidatus_Nitrosopelagicus*, *Cenarchaeum*, *Haladaptatus*, *Halogranum*, *Halolamina,* and *Woesearchaeales* and *GW2011-AR15* in juveniles. All results revealed flexible symbiotic mechanisms in *D. veroni* during early ontogeny for coral survival and evolution.

**IMPORTANCE:**

Flexible symbioses of Symbiodiniaceae, bacteria, and archaea appear to be a heritable process of selection and adaptation in *Dipsastraea veroni* in the field, benefiting early coral development and facilitating coral population recovery and reef conversation.

## INTRODUCTION

Coral reefs, one of the world’s most productive and biodiverse ecosystems, have been severely degraded due to climate change and anthropogenic disturbance (1, 2). Rapid declines in the cover of scleractinian corals, which form the framework of coral reefs and their diversity, have resulted in 19% loss and 35% severe damage to the world’s coral reefs (3) although a 2% recovery has been observed (4). Coral reef restoration requires the coral sexual reproduction and recruitment (5–7) and symbiosis with Symbiodiniaceae, bacteria, and archaea (8–10).

Sexual reproduction and recruitment are key processes for the maintenance and evolutionary adaptation of coral populations and the persistence of coral reef ecosystems (6, 11, 12). Sexual reproduction introduces offspring with genetic variation (13, 14) to replenish depleted adult populations and promote population recovery and evolution. Approximately half of the estimated 900 scleractinian corals have been studied for sexual reproduction (6, 11, 15), but the complex life cycle of the coral holobiont makes it challenging to identify symbiosis establishment and mechanisms during early development and recruitment with high mortality (5, 16).

Coral evolution is also influenced by symbiosis (9, 17). Corals provide a protective environment and essential compounds for symbionts (Symbiodiniaceae, bacteria, and archaea), which are fundamental to coral survival and evolution through energy supply, nutrient transfer, and genetic exchange (12, 17, 18). Symbiodiniaceae, a family of dinoflagellate microalgae that provide photosynthetic energy and/or nutrients via carbon fixation (19, 20), translocate up to 90% of the coral carbon demand (21, 22). Bacteria and archaea are involved in the transfer of trace metals, vitamins and products of carbon, nitrogen, and sulfur fixation, and metabolism to corals (8, 23–25) and/or against pathogens (9, 10).

To establish symbiosis, approximately 85% of scleractinian corals exhibit horizontal acquisition (assimilated from the environment) and 15% vertical transmission (inherited from parents) (11). Flexible symbiosis with more dynamic microbial communities through mixed-mode transmission or both simultaneously or in succession allows winnowing (elimination of certain symbionts) for favorable symbionts that may contribute to coral survival and evolution (26–28). Coral surface mucus plays an important role in these beneficial symbioses (29–31).

*Dipsastraea*, one of the stress-tolerant genera widely distributed in the Indo-Pacific, has persisted (32, 33) and/or recruited to coral reefs under environmental disturbance (34, 35). Among them, *Dipsastraea veroni* (Moll & Best, 1984), a rare massive reef-building coral on reef slopes, is moderately tolerant and listed as Near Threatened (https://www.iucnredlist.org/species/132894/3481551). In this study, *D. veroni* larvae from artificial sexual reproduction were settled and metamorphosed into juveniles in flow-through *in situ* seawater to study the onset and dynamics of symbiosis during early life stages. Community composition and diversity of Symbiodiniaceae, bacteria, and archaea were determined using inter-transcribed spacer region 2 (ITS2) and 16S ribosomal RNA gene (16S rDNA) amplicon sequencing of corals at adult, egg, larva, juvenile 1 (at 5 days post settlement, d p.s.), and juvenile 2 (at 32 d p.s.) stages. The flexible and specific symbiont-host association in *D. veroni* is critical for early coral development and could facilitate coral population recovery and reef conversation.

## MATERIALS AND METHODS

### Spawning event and coral larvae culture

**Fig 1:**
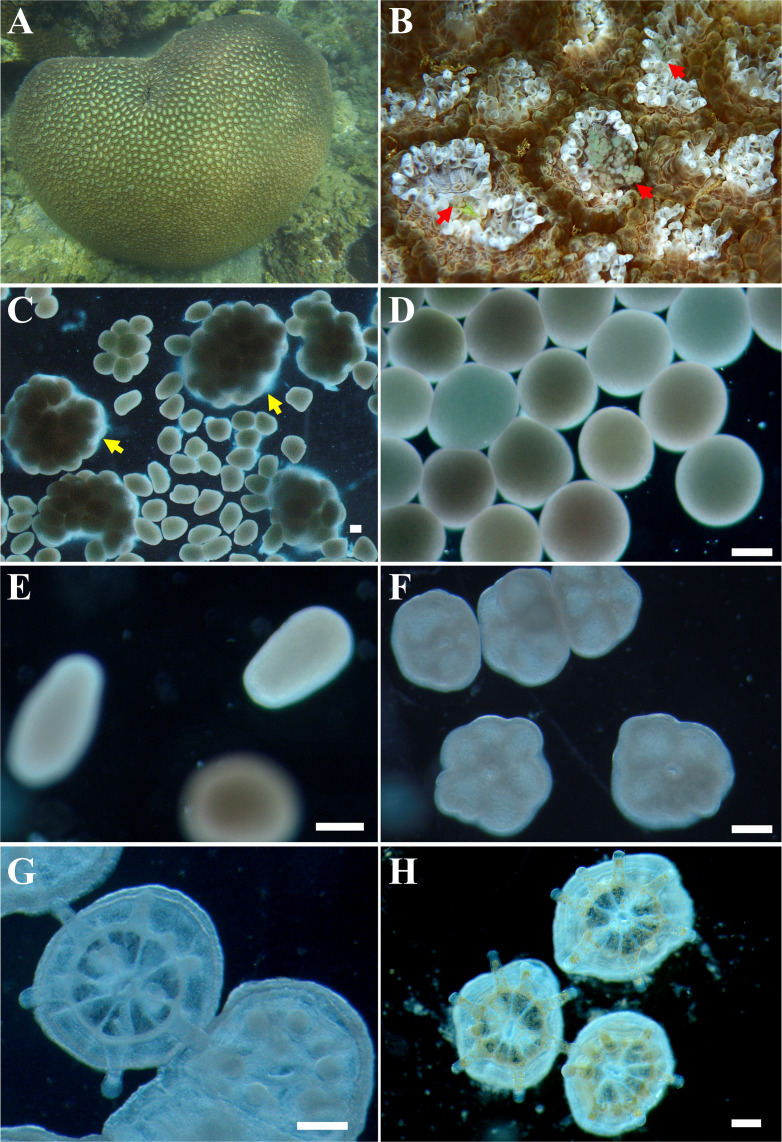
Photos of *D. veroni* at different life stages. (**A**) Adult *D. veroni* on the Luhuitou coral reef, Sanya Bay, China. (**B**) Slow extrusion of egg-sperm bundles in *D. veroni*. (**C**) Buoyant egg-sperm bundles. (**D**) Fertilized eggs (~1 h). (**E**) Planula larva. (**F–H**) Juveniles at 2, 5, and 32 d p.s., respectively. Bar = 200 µm.

Six gravid colonies of *D. veroni* (Fig. 1A, ~25 cm in diameter) were collected from 4 to 6 m depth at Luhuitou coral reef (18°12′N, 109°28′E), Sanya Bay, China, on 7 May 2020. They were maintained in flow-through *in situ* seawater (pumped from ~5 m depth) under natural light-dark cycles (~250 µmol photons per square meter per second at noon) at the Tropical Marine Biological Research Station in Hainan. Live brine shrimp *Artemia salina* nauplii, which hatched for approximately 36 hours (h), were fed to the corals every night until the corals spawned on 14–16 May (21–23 April, lunar date) (Fig. 1B). Egg-sperm bundles (Fig. 1C) were gently mixed for natural gamete fertilization (Fig. 1D) in *in situ* seawater filtered through a 5 µm filter bag. The embryos developed into swimming larvae (Fig. 1E), which were temporarily cultured in an aquarium filled with 2,000 L of filtered seawater above.

### Experimental setup

Prior to the experiments, 6 aquaria (volume ~90 L, outlet covered with 180 µm mesh) containing 15 cm Petri dishes (10 per aquarium) fixed with Gel-10 glue (Aron Alpha, Japan) were incubated with flow-through (flow rate ~50 mL/min) sand-filtered *in situ* seawater under natural light-dark cycles for 2 days. Settlement and juvenile growth were performed in these aquaria (mimicking field symbiosis) thermostatically controlled at 27.93 ± 0.96°C (Weipro, China). On 19 May, ~1,000 4-day-old larvae per aquarium were then placed on petri dishes with terracotta tiles (3.6 cm in diameter, field-preconditioned for 2 weeks). Larvae settled and metamorphosed on 21 May (Fig. 1F) when meshes were removed to release unsettled larvae. Juveniles continued to grow for 1 month. Images of eggs and juvenile polyps were taken with a light microscope (SZX7, Olympus, Japan) to count survivorship and measure egg diameter and juvenile surface area (lateral growth) using ImageJ software (National Institutes of Health) (36).

### Sample collection

Adult (18 May, Fig. 1A), egg (15 May, Fig. 1D), larva (19 May, Fig. 1E), juvenile 1 (24 May, Fig. 1G), and juvenile 2 (21 June, Fig. 1H) corals were sampled in triplicate. In detail, a small piece (~2 cm diameter) of polyp tissue was obtained from each adult (two per group) and primary polyp from 80 juveniles in each tank using a sterile scalpel. Approximately 80 eggs and/or larvae were pipetted onto 180 µm mesh for each of the three groups. Meanwhile, three replicates of 2 L of sand-filtered ambient seawater were filtered onto the 0.2 µm polycarbonate membranes as background. All samples were thoroughly washed three times with 0.22 µm filtered seawater, preserved in absolute ethyl alcohol, and stored at −20°C for the following experiment. Adults recovered for days and returned to the original reef.

### Scanning electron microscopy (SEM) and transmission electron microscopy (TEM)

Three groups of *D. veroni* eggs or larvae (~60 individuals per group) were washed three times as above and fixed in 2.5% (vol/vol) glutaraldehyde (Sigma, St.Louis, MO, USA) in 0.22 µm filtered seawater for 2 h at room temperature and ~20 h at 4°C for SEM. Parallel samples were fixed in 2.5% glutaraldehyde and 2% paraformaldehyde of 0.22 µm filtered seawater as above for TEM. On 12 April, the coral *Montipora turgescens* spawned the eggs fixed above as a control.

For SEM, samples were washed three times with phosphate buffer (PBS, 30 min/time). Dehydration was performed in a graded series of ethanol (30%, 50%, 70%, 90%, 95%, and 100%) for 20 min. Eggs or larvae were incubated with absolute ethanol and desiccated using a critical point dryer (Leica EM CPD300, Germany). After reaching the critical point, 15 individuals per replicate were split with dissecting needles on a copper foil for metallization of gold and platinum conduction using an ion sputtering (E-1010, Hitachi, Japan, 20 Kv). The copper foil was placed on an aluminum stub to visualize the cross section using a scanning electron microscope (Hitachi S-3400N, Japan).

For TEM, samples were washed five times in PBS (15 min/time) and postfixed for 1–2 h in 1% OsO_4_ (Sigma, St. Louis, MO, USA) in PBS. After three washes in PBS, eggs or larvae were dehydrated in a graded series of acetone (50%, 70%, 90%, and 100%) (20 min/time) and 100% acetone (20 min/time, three times), infiltrated in baths mixing acetone with Spurr’s resin in graded proportions (2:1, 1:2) for 3–4 h and 100% resin for 24 h, and embedded in 100% resin using a hot air oven (60°C) for 72 h. Ultrathin sections (50–70 nm) prepared on an Ultracut E microtome UC-7 (Leica, Germany) were placed on copper grids, stained with 5% uranyl acetate and Reynold’s lead citrate, and photographed using a transmission electron microscope (Hitachi H-7500, Japan).

### DNA extraction and amplicon sequencing

Absolute ethyl alcohol was removed by centrifugation at 12,000 *g* for 15min. Total genomic DNA was extracted from all samples using the Fast DNA SPIN Kit for soil (MP Biomedicals, Irvine, USA) according to the manufacturer’s instructions. Sample disruption was performed using a FastPrep-24 instrument (MP Biologicals, USA) for 1 min at a speed of 6 m/s. The eluted DNA was analyzed using a NanoDrop 2000 spectrometer (Thermo Fisher Scientific, USA) with a threshold of 1.8–2.0 for A260/280 and stored at −20°C until amplicon PCR amplification.

Primer pairs ITS2F (5′-GAATTGCAGAACTCCGTG-3′) and ITS2R (5′-GGATCCATATGCTTAAGTTCAGCGGGT-3′) (37), Bac341F (5′-CCTAYGGGRBGCASCAG-3′), and Bac802R (5′-TACNVGGGTATCTAATCC-3′) (38) were used to amplify the Symbiodiniaceae ITS2 gene and the bacterial 16S rDNA V3-V4 region, respectively. The primer pair SeqF2: 5′-GYMGCCRCGGKAAHAS-3′; SeqR2: 5′-NYRTACTYCCCARGYRG-3′ was designed for the archaeal 16S rDNA V4-V5 region (39). Each primer pair was mixed with the template DNA, Phusion High-Fidelity PCR Master Mix (New England Biolabs) according to the manufacturer’s protocol. PCR conditions consisted of denaturation for 4 min at 95°C, followed by 35 cycles of 30s at 95°C, 30 s at 55°C (bacterial 16S rDNA or Symbiodiniaceae ITS2) or 59°C (archaeal 16S rDNA) and 30 s at 72°C, and a final extension for 5 min at 72°C.

PCR products were purified using the Qiagen Gel Extraction Kit (Qiagen, Germany) to prepare libraries using the TruSeqDNA PCR-Free Sample Preparation Kit (Illumina, USA) following the manufacturer’s instructions. The purified libraries were checked using the Qubit@ 2.0 fluorometer (Thermo Fisher Scientific, USA) and the Agilent Bioanalyzer 2100 system (Agilent Technologies, Santa Clara, USA). Sequencing was then performed on an Illumina NovaSeq platform using 250 bp paired-end reads (Novogene, Beijing).

### Data Processing and statistical analysis

Raw data were processed into fastq files using UCHIME (V1.17) (40). Paired-end reads were assigned to samples based on barcodes and assembled into raw tags using FLASH (V1.2.7) (41). After quality filtering with QIIME (42), the clean reads were compared with the modified Symbiodiniaceae ITS2 database (1 , Supplementary information by BLASTN against NCBI database, clade, and subclade were renamed as genus and species) (43, 44) and the Silva database (https://www.arb-silva.de/) (16S rDNA) using UCHIME to remove the chimera sequences. Effective sequences with 97% similarity were assigned to operational taxonomic units (OTUs) (>2 sequences) using the Uparse software (V7.0.1001) (45). Taxonomic annotation and quantitative analysis of OTUs at the phylum, class, order, family, genus, and species level were performed using the BLASTN against the modified ITS2 database and the Mothur algorithm (46) against the Silva database at 70% confidence.

Relative abundances of OTUs were used for diversity and statistical analyses using QIIME (V1.9.1) and the Vegan package in R software (V2.15.3) (47). Alpha diversity (Richness, Shannon, Chao1, Simpson, ACE, and Good-coverage) was generated to estimate species complexity within the community. Comparative analyses of Richness (species) and Shannon were performed using a Wilcoxon rank-sum test. A square root-transformed Bray-Curtis dissimilarity based Principal Coordinate Analysis (PCoA) was performed to show the symbiont relationships at different stages. Adonis in Vegan package was used for the permutational multivariate analysis of variance based on Bray-Curtis dissimilarity to determine the statistical significance of stage separation and relationships. To identify stage-associated symbionts, relative abundances of symbionts were detected by linear discriminant analysis (LDA) effect size (LEfSe, V1.0), with LDA >4.00 and Wilcoxon rank-sum test *P* < 0.05 (48) .

All data were expressed as the mean ± standard deviation of three independent experiments. Tukey’s HSD multiple comparisons in SPSS (26.0) software were performed as post hoc tests when one-way analysis of variance (ANOVA) with *P* values < 0.05 detected significant differences in surface area, sequence number, and/or relative abundance of symbionts at different life stages.

## RESULTS

### Sexual reproduction and early development of *D. veroni*

*D. veroni* is a broadcast spawning hermaphrodite coral. Slow extrusion of gamete bundles containing both egg and sperm occurred (Fig. 1B and C) at ~22:30 for approximately 40 min during the sixth to eighth (peak on the seventh) night after the full moon in ambient seawater at 28.36 ± 0.15°C. Eggs were aqua (pale green/blue), pink, and tan (Fig. 1B through D) with a mean diameter of 405.60 ± 4.64 µm (Table S1-1). Each adult produced approximately 500,000 larvae (fertilization ~92%). It took ~48 h for the larvae to settle and metamorphose into juveniles (Fig. 1E and F).

Lateral growth of juveniles was significantly (*P* = 0.000) faster in the first half of the month (0.34 ± 0.04 mm^2^) than in the second half (0.08 ± 0.02 mm^2^) based on the surface areas at 2, 17, and 32 d p.s. (Fig. S1; Table S1-2). Brown Symbiodiniaceae became visible in polyp tentacles at 5 d p.s. (Fig. 1G) and thrived at 32 d p.s. (Fig. 1H) when the survivorship was 90.14 ± 0.04%.

### Ultrastructure of eggs and larvae

**Fig 2:**
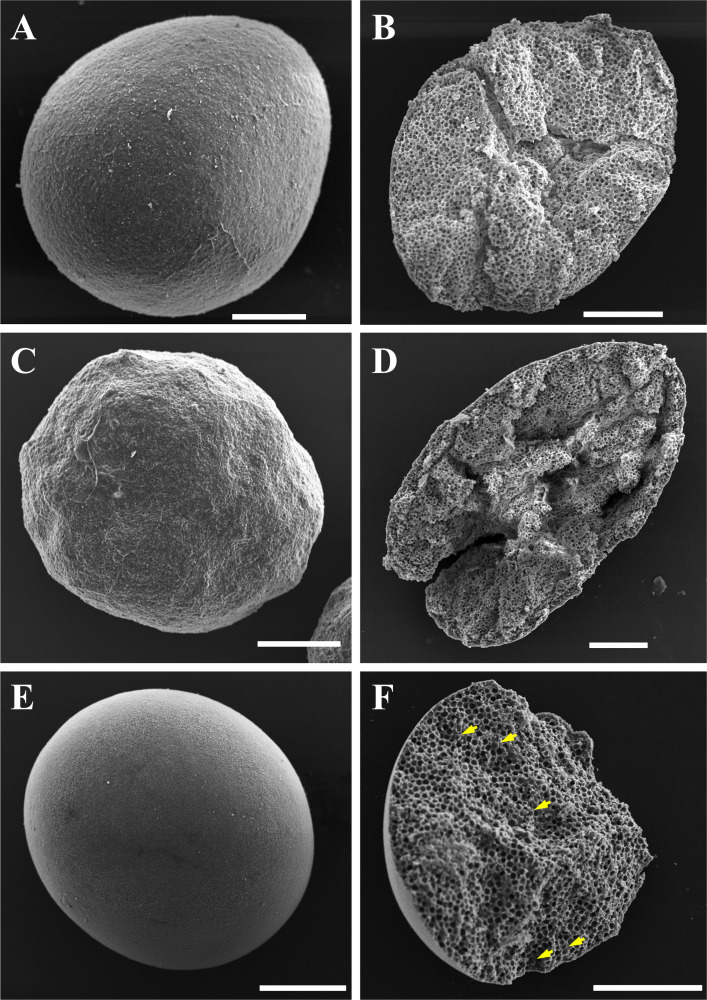
Scanning electron microscopy of eggs and larvae. (**A** and **B**) Surface and cross section of *D. veroni* egg. (**C** and **D**) Surface and cross section of *D. veroni* larva. (**E** and **F**) Spherical *M. turgescens* egg and its cross section with symbiotic Symbiodiniaceae (yellow arrows). Bar = 100 µm.

The surface of eggs and larvae was not smooth but attached with small particles (Fig. 2A, C, and E). All cross sections were occupied by ~5 µm diameter empty spherical spaces, except for the peripheral layer of ~5 µm yolk bodies and/or cortical vesicles (Fig. 2B, D, and F; Fig. S1B). Symbiodiniaceae cells did not appear in *D. veroni* eggs and larvae (Fig. 2B and D), but in *M. Turgescens* eggs (Fig. 2F, yellow arrows).

**Fig 3:**
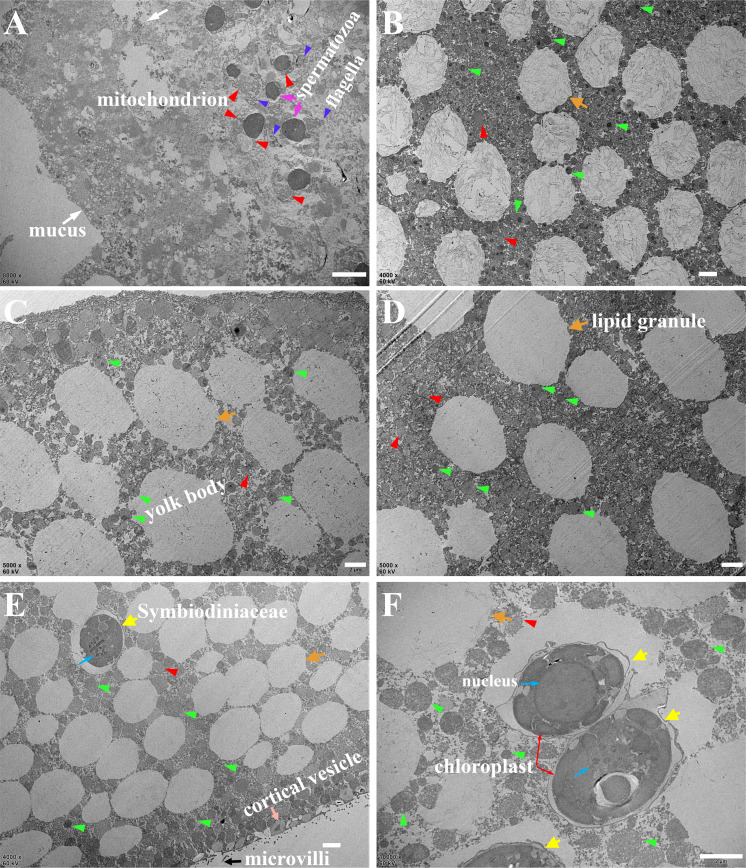
Transmission electron microscopy of eggs and larvae. (**A**) Mucus (white arrows) adhering to the egg-sperm bundles (magenta arrows). Mitochonria, red arrowheads; flagella, violet arrowheads. (**B**) Cross section of *D. veroni eggs* and larvae. (**C and D**) Yolk bodies, green arrowheads; lipid granules, orange arrows. (**E** and **F**) Symbiotic Symbiodiniaceae cells (yellow arrows) with nucleus (turquoise arrows) and chloroplast (red arrows) vertically transmitted in *M. Turgescens* eggs. Bar = 2 µm.

Mucus (white arrows) was attached to spermatozoa (magenta arrows, on the surface of the egg), which contained mitochonria (red arrowheads) and flagella (violet arrowheads) (Fig. 3A). *D. veroni* eggs (Fig. 3B) and larvae (Fig. 3C and D) were full of yolk bodies (green arrowheads) and lipid granules and some mitochondria, but no symbiotic microorganisms and/or Symbiodiniaceae cells (yellow arrows), which were vertically transmitted in *M. Turgescens* eggs (Fig. 3E and F). Dehydration resulted in the empty spherical spaces in the SEM samples (Fig. 2B, D and F; Fig. S1B) due to the dissolution of lipid granules, which were still visible in the TEM samples due to OxO_4_ treatment (Fig. 3B through F, orange arrows).

### Data and sequence information

Eighteen samples were obtained with 72,730 normalized reads (Table S2-1) clustering into 1,054 OTUs blasted to 10 genera in the family Symbiodiniaceae (Table S4-1). For bacterial and archaeal 16S rDNA, there were 39,817 and 27,484 normalized reads (Table S2-1) clustering into 7,732 (Table S4-2) and 9,181 OTUs (Table S4-3), respectively. Bacterial OTUs were annotated to 496 genera in 30 phyla. However, 2,154 archaeal OTUs were actually annotated to 66 genera in 12 phyla (Table S4-3). Sequencing and diversity of all samples reached a plateau based on rarefaction curves, rank-abundance curves (Fig. S2), and goods coverage (>0.96) (Table S2-1).

**Fig 4:**
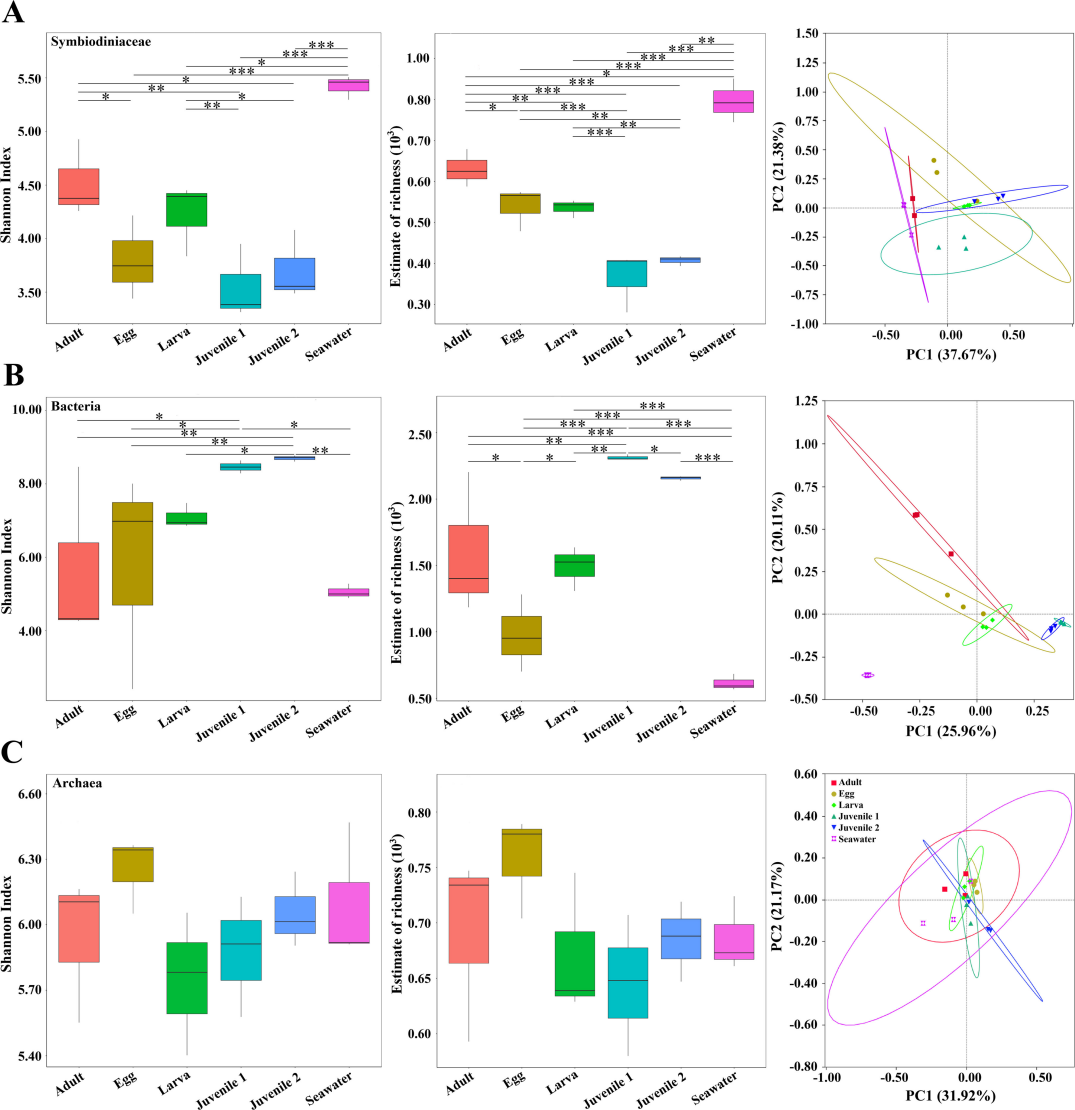
Diversity statistics of Shannon index, richness, and PCoA in *D.veroni* at different life stages. Box plots from left to right represent Shannon diversity, richness, and PCoA visualization based on Bray-Curtis dissimilarity of OTUs for Symbiodiniaceae (**A**), Bacteria (**B**), and Archaea (**C**). The PCoA axes represent the two synthetic variables that explain most of the variation in the data set. ^*^P < 0.05, ^**^P < 0.01, ^***^P < 0.001.

For Symbiodiniaceae ITS2, both Shannon diversity and richness were significantly higher in seawater than in *D. veroni,* which differed among stages except for egg and larva (Fig. 4A). For 16S rDNA, bacterial Shannon index was significantly higher in juvenile 2 than others, but similar to juvenile 1, whose richness was significantly higher than others (Fig. 4B), while no significant differences were found in Archaea (Fig. 4C; Table S2-2). PCoA followed this partitioning, with samples clustering into groups in Symbiodiniaceae (Fig. 4A), Bacteria (a clear separation of corals from seawater) (Fig. 4B), and Archeae (Fig. 4C; Tables S2-2 and S3-1).

In community composition, Symbiodiniaceae ITS2 differed significantly (*P* < 0.01) between adult and egg, larva and juvenile 1, juvenile 1 and juvenile 2, and egg, larva, or juvenile 1 and seawater. Bacterial 16S rDNA changed significantly between juvenile 2 and other stages, adult and egg or larva, and larva and juvenile 1, while archaeal 16S rDNA was stable among stages (*P* > 0.05) (Table S3-2).

### Specific transmission of Symbiodiniaceae in *D. veroni*

**Fig 5:**
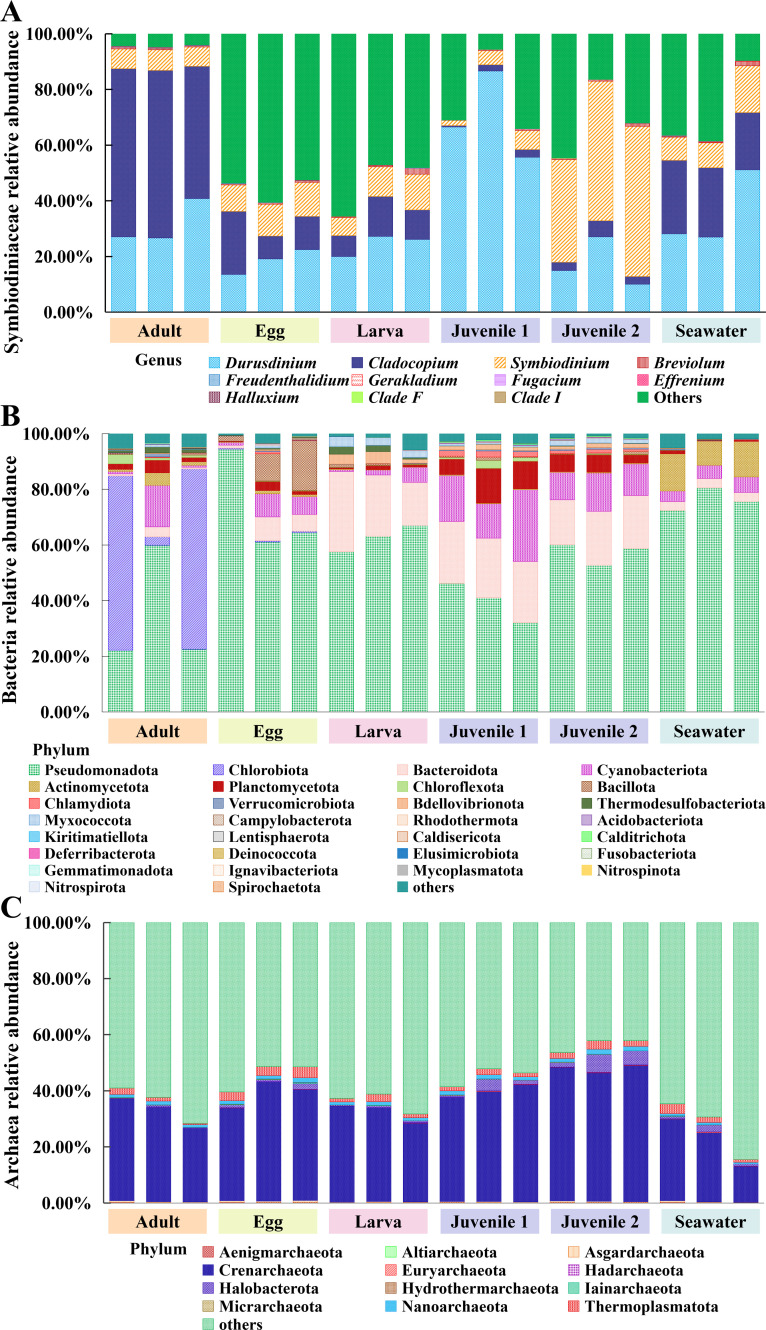
Relative abundance of symbionts in *D. veroni* at different life stages. (**A**) Symbiodiniaceae at the genus level. (**B**) Bacteria and (**C**) Archaea at the phylum level. Others represent the taxa that do not belong to the corresponding symbionts.

All groups were dominated by *Durusdinium*, *Cladocopium,* and *Symbiodinium* (Fig. 5A). Consistent with the sequence number (Fig. S1C), the relative abundance of Symbiodiniaceae ITS2 in the adult (95.54%) was significantly higher than that in the egg (44.39%) and larva (46.40%) (Fig. 5A). Adult was associated with *Durusdinium* (31.48%), *Symbiodinium* (7.12%), and especially *Cladocopium* (56.05%), which was significantly higher than egg (12.29%), larva (10.87%), juvenile 1 (1.87%), juvenile 2 (3.88%), and seawater (23.95%). Adult also had rare (<1.00%) *Freudenthalidium, Fugacium*, and *Gerakladium*, and *Halluxium* were lower than seawater. Juvenile 1 and juvenile 2 were rich in *Durusdinium* (69.59%) and *Symbiodinium* (46.95%), respectively (Tables S4-1, S5-1 and S5-2).

In *Cladocopium*, the stage-abundant GS_C3u, LJ_C2r, GS_C3w in the adult (Fig. S3A) were significantly eliminated in the juvenile (Tables S4-1 and S5-4), and presumably to be horizontal acquisition, which also occurred in *GS_A8*, *GS_I1* and *GS_I2*. The stage-abundant *LD_D17*, *GS_D6*, *GS_D2.2* of *Durusdinium* and *GS-A6* and *GS-A3* of *Symbiodinium,* with similar relative abundance in egg and/or larva as in adult, were significantly increased in juvenile 1 and juvenile 2, respectively (Fig. S3A; Tables S4-1, S5-3 through S5-5).

### Flexible symbiosis of Bacteria in *D. veroni*

Bacterial community in *D. veroni* was different from that in seawater and varied due to flexible symbiosis during early ontogeny (Fig. 5B; Tables S5-1 and S5-2). Horizontal acquisition, likely from seawater, occurred in the phyla Chlorobiota, Deinococcota, Spirochaetota, Fusobacteriota, Pseudomonadota, and Bacillota, the latter two with significantly higher relative abundance in egg (73.38% and 9.88%) than adult (36.59% and 0.42%) and juvenile 1 (39.77% and 0.65%). Phyla present in the egg were winnowed in the juvenile, such as Caldisericota, Chlorobiota, Gemmatimonadota, Ignavibacteriota, Kiritimatiellota, Lentisphaerota, and Mycoplasmatota. Phyla present in both adult and egg that significantly increased their relative abundance in larva, juvenile1, and/or juvenile 2 included Acidobacteriota, Bacteroidota, Bdellovibrionota, Calditrichota, Chlamydiota, Chloroflexota, Cyanobacteriota, Myxococcota, Planctomycetota, and Thermodesulfobacteriota (Tables S4-2, S5-1 and S5-2).

Stage-abundant genera included *Prosthecochloris* in adult, *Pseudomonas*, *Acinetobacter*, *Chelatococcus*, *Achromobacter*, *Brevibacillus*, *Bacillus,* and *Anoxybacillus* in egg, *Vibrio*, *Nautella*, *Shimia*, *Oleiphilus*, *Nonlabens,* and *Mesoflavibacter* in larva, *Reichenbachiella*, *Kordia,* and *Teredinibacter* in juvenile 1, *Rhodovulum* in juvenile 2 (Fig. S3B; Tables S4-2, S5-3 and S5-4). About a quarter of the genera in the adult were all present in egg, larva, juvenile 1, and juvenile 2, others were winnowed and/or probably acquired horizontally from seawater (Table S4-2).

Coral development drove the bacterial dynamics of *D. veroni* based on the variation in relative abundance of genera (Tables S4-2, S5-3 through S5-5). Significantly abundant genera in larvae were those in the orders Rhodobacterales, Oceanospirillales, Vibrionales, Flavobacteriales, and Chitinophagales. Juveniles had abundant genera in the orders Cyanobacteria, Rhodospirillales, Rhodobacterales, Gammaproteobacteria, Chitinophagales, Bdellovibrionales, Sphingomonadales, Alphaproteobacteria, Caulobacterales, Nostocales, Alteromonadales, Cellvibrionales, Oceanospirillales, Cytophagales, Flavobacteriales, Planctomycetales, and Phycisphaerales. While adults had genera in the orders Chlorobiales, Chromatiales, Nostocales, Chloroflexales, Rhodospirillales, Rhizobiales, Desulfobacterales, and Desulfarculales (Table S5-5).

### Stable archaeal community with high diversity in *D. veroni*

Similar to the Archaea sequence number (Fig. S1C), the relative abundance in seawater (27.18%) was significantly lower than that in egg (45.60%), juvenile 1 (45.23%), and/or juvenile 2 (56.46%), which was higher than that in adult (35.69%) and larva (35.97%). Crenarchaeota, Hadarchaeota, Halobacterota, and Nanoarchaeia contributed to this shift although all phyla in egg or juvenile 1 were similar to other stages (Fig. 5C; Tables S5-1 and S5-2). The dominant Crenarchaeota, Halobacterota, Nanoarchaeia, and Thermoplasmatota (>97.38% of total sequences) and the rare Aenigmarchaeota, Altiarchaeota, Asgardarchaeota, Euryarchaeota, Hadarchaeota, Hydrothermarchaeota, Iainarchaeota, and Micrarchaeota in the adult were detected in the offspring at all four stages (Fig. 5C; Table S4-3).

Community composition varied only in the relative abundance of some genera. *Methanobrevibacter*, *Haladaptatus*, *Haloarchaeobius*, *Halolamina, Haloferax*, *Candidatus_Halobonum, SCGC AAA011-D15, SCGC AAA286-E23,* and *Natronomonas* were horizontally acquired from seawater (Tables S4-3, S5-3 and S5-4). Stage-abundant genera were *Candidatus_Nitrososphaera* and *Hadarchaeales* in adult, *Bathyarchaeota*, *Candidatus_Nitrosopelagicus, Candidatus_Nitrosotenuis, Nitrosarchaeum*, *Candidatus_Nitrocosmicus*, *Haladaptatus*, *Halolamina*, *Halogranum,* and *GW2011-AR15* in juvenile 2 (Table S5-5). Both stages differed in *Halogranum*, *Candidatus_Nitrosopelagicus,* and *Candidatus_Nitrososphaera* (Fig. S3C). *Candidatus_Nitrosopumilus*, *Cenarchaeum, Woesearchaeales* were more abundant in corals than in seawater (Tables S4-3, S5-3 and S5-4).

## DISCUSSION

Over three-quarters of scleractinian corals are broadcast spawners (11, 15). The broadcast spawning hermaphrodite *D. veroni* is consistent with reports of gametes with different egg colors (49), a common sexual reproduction in *Dipsastraea* (11, 50), and close egg diameters (51, 52). Synchronized mass spawning in corals appears to be driven by moonrise time (53), lunar cycle, and environmental factors (6, 11). Coinciding with low amplitude tides in Sanya Bay, *D. veroni* spawned approximately 4.5 h after sunset on the sixth to eighth night after the full moon, which is similar to *Dipsastraea* species on other coral reefs in different months (49, 50, 54). This may be related to seawater temperature (6, 11), which increases by over 1℃ for *D. veroni* compared to *Acropora* corals (March–April).

Juvenile growth of *D. veroni* is slow compared to branching corals (52, 55). Like other lipid-rich and lecithotrophic larvae (11), *D. veroni* larvae appear to contain sufficient lipid and yolk bodies for the juveniles to grow faster in the first half of the month than in the second half, which is still a winnow for favorable symbionts to proliferate and thrive. Life history traits of *D. veroni*, such as large size at the onset of reproduction, slow growth, and broadcast spawning species with high fecundity, support a stress-tolerant *Dipsastraea* species (33, 56).

Coral mucus is essential for the regulation of microbial populations (29), particle trapping (31), and adaptation (30). Ultrastructural observation revealed no symbionts in mucus-coated *D. veroni* eggs and/or larvae. However, sequencing showed transmission from the adult based on symbionts that were significantly more abundant in the egg and/or larva than in seawater (such as *GS-A6, unidentified_Bathyarchaeota, Candidatus_Nitrosopumilus,* etc.) or absent from seawater (such as *Prosthecochloris*, *Rhodobacteraceae, Pseudomonas*, etc.). This hypothetical vertical transmission via the mucus coating of gamete bundles with maternally derived symbionts or the surrounding seawater released from the parent colony has been occurred in spawning corals (57, 58). Vertical transmission with tight coevolution from partners may increase genetic bottlenecks (6, 59), whereas flexible symbioses facilitate coral survival and evolution (27, 28). Similar to *Dipsastraea* species (60, 61), *D. veroni* showed a flexible symbiosis with bacteria.

Unlike the azooxanthellae eggs of over 80% of broadcast-spawning corals (11), *D. veroni* appears to acquire Symbiodiniaceae from seawater and/or mucus contamination of adult, including the tropically diverse and ancestral *Cladocopium* (62, 63), along with the thermotolerant *Durusdinium* (64, 65) and the *Symbiodinium* with extensive carbon and nitrogen transporters (66). *Durusdinium* (LJ-D17, GS_D6, and GS_D2.2) increased significantly in juvenile 1 but decreased in juvenile 2, suggesting a heritable flexibility. Rare Symbiodiniaceae in *D. veroni* may contribute to coral survival and stress-tolerant traits (63, 67).

The dominant Pseudomonadota, Bacteroidota, Cyanobacteriota, Bacillota, Planctomycetota, and Actinomycetota in *D. veroni* are similar to other reef-building corals (8, 68). Bacterial transmission appears to be related to coral development and environment, but not to sexual reproduction. Unlike broadcast spawning (69, 70) or brooding corals (71, 72), *D. veroni* juvenile acquired a greater diversity of bacteria than adult and differed from *Dipsastraea* species on other reefs (61, 73). Bacterial winnowing in larvae and/or juveniles suggests a flexible transmission mechanism as *D. veroni* develops. Environmental conditions influence juvenile bacterial communities more than maternal colonies (70, 72).

Corals prefer symbionts with specific functions at different stages. *D. veroni* adults had abundant photoautotrophic bacteria, including Chloroflexales, Cyanobacteriales, and Chlorobiales, which dominated the green layer of the coral skeleton (25, 61). Inconsistently, *D. veroni* larvae and/or juveniles contained photosynthetic bacteria such as Rhodobacterales, Rhodospirillales, Cyanobacteria, and Cyanobacteriales, which may provide photosynthetic products by carbon fixation (70) or metabolites from the sulfur (23) and/or nitrogen cycle (74). Photosymbionts, drivers of coral habitat selection (63, 75), may facilitate the survival and evolution of *D. veroni.* Stage-abundant Pseudomonadales and Bacillales in eggs may contribute to *D. veroni* defense against pathogens, as in other corals (29, 76).

Archaea may be abundant and not specifically associated with coral species (77, 78), but more geographically dependent (79). Archaeal symbiosis appeared to be coral and environment dependent. Compared to corals associated with 2–3 phyla (24, 80), *D. veroni* hosted 12 phyla of archaea with higher relative abundance than seawater, most by hypothetical vertical transmission and a few by horizontal acquisition. Archaea appear to favor juvenile survival, as more Crenarchaeota (*Bathyarchaeota, Candidatus_Nitrosopelagicus*, *Nitrosarchaeum*, *Candidatus_Nitrosopumilus*, *Candidatus_Nitrocosmicus,* and *Candidatus_Nitrosotenuis*), Halobacterota (*Haladaptatus*, *Halogranum* and *Halolamina*), and Nanoarchaeota (*GW2011-AR15* and *Woesearchaeales*), together with other rare phyla, are potentially involved in the carbon, nitrogen, sulfur, and iron cycling (24, 81).

In conclusion, *D. veroni* is a broadcast spawner. Lipid granules and yolk bodies contribute to the survival and growth of *D. veroni* at early life stages when symbiosis with Symbiodiniaceae, bacteria, and/or archaea appears to be a heritable process driven by coral development. Symbiotic establishment in *D. veroni* involved horizontal acquisition and hypothetical vertical transmission via the mucus coat with symbionts from the parent. Stage-abundant Symbiodiniaceae varied among the dominant *Cladocopium*, *Durusdinium,* and *Symbiodinium*, while bacteria varied among most phyla, especially those containing photoautotrophic and photosynthetic bacteria. The flexible symbiosis of bacteria in *D. veroni* shows a winnowing for favorable symbionts. The stable archaeal community is highly diverse, but with few stage-abundant genera. Reproductive traits and symbiotic mechanisms benefit *D. veroni* for survival and evolution during early ontogeny.

## Data Availability

All raw sequences have been submitted to the NCBI Sequence Read Archive as BioProject PRJNA811408, PRJNA810708, and PRJNA1056452. All data sets associated with this study are included in the article and the supplemental material.

## SUPPLEMENTAL MATERIAL

Data Set S1 aem.02359-24-s0001.doc Modified Symbiodiniaceae ITS2 database.

Supplemental figures aem.02359-24-s0002.pdf Figures S1 to S3.

Supplemental tables aem.02359-24-s0003.xlsx Tables S1-1 to S5-5.

ASM does not own the copyrights to Supplemental Material that may be linked to, or accessed through, an article. The authors have granted ASM a non-exclusive, world-wide license to publish the Supplemental Material files. Please contact the corresponding author directly for reuse.
