# Supplementary material for: Diversity and dynamics of multiple symbionts contribute to early development of broadcast spawning reef-building coral Dipsastraea veroni
Source: Appl Environ Microbiol. 2025 Jan 29;91(2):e02359-24. doi: 10.1128/aem.02359-24 (PMC11837535; doi:10.1128/aem.02359-24)
Supplement: Data Set S1 — Modified Symbiodiniaceae ITS2 database. [file aem.02359-24-s0001.doc]

| >GS_A1 (*Symbiodinium*) |
| --- |
| AACCAATGGCCTCTTGAACGTGCATTGCGCTCTTGGGATATGCCTGAGAGCATGTCTGCT |
| TCAGTGCTTCTACTTTCATTTTCTGCTGCTCTTGTTATCAGGAGCAGTGTTGCTGCATGC |
| TTCTGCAAGTGGCACTGGCATGCTAAATATCAAGTTTTGCTTGCTGTTGTGACTGATCAA |
| CATCTCATGTCGTTTCAGTTGGCGAAACAAAAGCTCATGTGTGTTCTTAACACTTCCTAG |
| CATGAAGTCAGACAA |
| >GS_A1.1=A13 (*Symbiodinium*) |
| AACCAATGGCCTCTTGAACGTGCATTGCGCTCTTGGGATATGCCTGAGAGCATGTCTGCT |
| TCAGTGCTTCTACTTTCATCTTCTGCTGCTCTTGTTATCAGGAGCAGTGTTGCTGCATGC |
| TTCTGCAAGTGGCACTGGCATGCTAAATATCAAGTTTTGCTTGCTGTTGTGACTGATCAA |
| CATCTCATGTCGTTTCAGTTGGCGAAACAAAAGCTCATGTGTGTTCTTAACACTTCCTAG |
| CATGAAGTCAGACAA |
| >GS_A1.2 (*Symbiodinium*) |
| AACCAATGGCCTCTTGAACGTGCATTGCGCTCTTGGGATATGCCTGAGAGCATGTCTGCT |
| TCAGTGCTTCTACTTTCATTTTCTGCTGCTCTTGTTATCAGGAGCAGTGTTGCTGCATGC |
| TTCTGCAAGTGGCACTGGCATGCTGAATATCAAGTTTTGCTTGCTGTTGTGACTGATCAA |
| CATCTCATGTCGTTTCAGTTGGCGAAACAAAAGCTCATGTGTGTTCTTAACACTTCCTAG |
| CATGAAGTCAGACAA |
| >ST_A1a (*Symbiodinium*) |
| AACCAATGGCCTCTTGAACGTGCATTGCGCTCTTGGGATATGCCTGAGAGCATGTCTGCTTCAGTGCTTCTACTTTCATTTTCTGCTGCTCTTGTTATCAGGAGCAGTGTTGCTGCATGCTTCTACAAGTGGCACTGGCATGCTAAATATCAAGTTTTGCTTGCTGTTGTGACTGATCAACATCTCATGTCGTTTCAGTTGGCGAAACAAAAGCTCATGTGTGTTCTTAACACTTCCTAGCATGAAGTCAGACAA |
| >ST_STR1 (*Symbiodinium*) |
| AACCAATGGCCTCTTGAACGTGCATTGCGCTCTTGGGATATGCCTGAGAGCATGTCTGCTTCAGTGCTTCTACTTTCATTTTCTGCTGGTCTTGTTATCAGGAGCAGTGTTGCTGCATGCTTCTGCAAGTGGCACTGGCATGCTAAATATCAAGTTTTGCTTGCTGTTGTGACTGATCAACATCTCATGTCGTTTCAGTTGGCGAAACAAAAGCTCATGT |
| >LJ_A1c (*Symbiodinium*) |
| GTCTGACTTCATGCTAGGAAGTGTTAAGAACACACATGAGCTTTTGTTTCGCCAACTGAAACGACATGAGATGTTGATCAGTCATAACAGCAAGCAAAACTTGATATTTAGCATGCCAGTGCCACTTGCAGAAGCATGCAGCAACACTGCTCCTGATAACAAGAGCAGCAGAAAATGAAAGTAGAAGCACTGAAGCAGACATGCTCTCAGGCATATCCCAAGAGCGCAATGCACGTTCAAGAGGCCATTGGTT |
| >ST_A1med (*Symbiodinium*) |
| AACCAATGGCCTCTTGAACGTGCATTGCGCTCTTGGGATATGCCTGAGAGCATGTCTGCTTCAGTGCTTCTTATATCCACTTGTTGCTGCTGCTCTTTCGTTAAAGGGCAGCGTGGGTGCATGCTACTGCTTTGCAGCACTGGCATGCGTAGATTTGCTGCTTGCTCACTGGTTCGATTGATCACTTCATCAAATCTTGTCAGTGTTGCAAGTCTTGCGTATGTGTTTTGACACATCCTAGCATGAAGTCAGACAA |
| >GS_A2 (*Symbiodinium*) |
| AACCAATGGCCTCTTGAACGTGCATTGCGCTCTTGGGATATGCCTGAGAGCATGTCTGCT |
| TCAGTGCTTCTAACTCTCTATGTGTCCTGCTGCTCTCTCCCTTTGAGAGCAGTGCTGCTG |
| CATGCTACTGCGCTGCTGCACTGGCATGCTCAATATCAAGTTTTGCCCACTGGTTTGACT |
| TGATCATCATCTTGTCTTGCAGATTGGGTAAAATAGGAAATAGTGTTTGCAACACTTCCT |
| AGCATGAAGTCAGACAA |
| >GS_A3 (*Symbiodinium*) |
| AACCAATGGCCTCTTGAACGTGCATTGCGCTCTTGGGATATGCCTGAGAGCATGTCTGCT |
| TCAGTGCTTCTACTTTCTTTTCTGCTGCTCTTGTTATCAGGAGCAGTGCTGCTGCATGCT |
| TCTGCAATTGGCACTGGCATGCTAAGTACCAAGTTTCGCTTGCTGTTGTGACTGATCAAC |
| ATCTCATGTCGTTTCAGTTGGCGAAACAAAGGCTTGTGTGTTCCAACACTTCCTAGCATG |
| AAGTCAGACAA |
| >ST_A3a_TD1E (*Symbiodinium*) |
| AACCAATGGCCTCTTGAACGTGCATTGCGCTCTTGGGATATGCCTGAGAGCATGTCTGCTTCAGTGCTTCTACTTTCTTTTCTGCTGCTCTTGTTATCAGGAGCAGTGCTGCTGCATGCTTCTGCAATTGGCACTGGCATGCTAAGTACCAAGTTTCGCTTGCTGTTGTGACTGATCAACATCTCATGTCGTTTCAGTTGGCAAAACAAAGGCTTGTGTGTTCCAACACTTCCTAGCATGAAGTCAGACAA |
| >GS_A3b (*Symbiodinium*) |
| AACCAATGGCCTCTTGAACGTGCATTGCGCTCTTGGGATATGCCTGAGAGCATGTCTGCT |
| TCAGTGCTTCTACTTTCTTTTCTGCTGCTCTTGTTATCAGGAGCAGTGCTGCTGCATGCT |
| TCTGCAATTGGCACTGGCATGCTAAGTACCAAGTTTTGCTTGCTGTTGTGACTGATCAAC |
| ATCTCATGTCGTTTCAGTTGGCGAAACAAAGGCTTGTGTGTTCCAACACTTCCTAGCATG |
| AAGTCAGACAA |
| >GS_A4 (*Symbiodinium*) |
| AACCAATGGCCTCTTGAACGTGCATTGCGCTCTTGGGATATGCCTGAGAGCATGTCTGCT |
| TCAGTGCTTCTACTTTCATTTTCTGCTGCTCTTGTTATCGGGAGCAGTGTTGCTGCATGC |
| TTCTGCAAAAGGCACTGGCATGCTAAGTATCAAGTTTTGCTTGCTGTTCTGACTGATCAA |
| CATCTCATGTCGTTTCAGTTGGCGAAACAAAAGCTGAAGTGTGTTCTTAACACTTCCTAG |
| CATGAAGTCAGACAA |
| >GS_A4a (*Symbiodinium*) |
| AACCAATGGCCTCTTGAACGTGCATTGCGCTCTTGGGATATGCCTGAGAGCATGTCTGCT |
| TCAGTGCTTCTACTTTCATTTGCTGCTGCTCTTGTTATCGGGAGCAGTGTTGCTGCATGC |
| TTCTGCAAAAGGCACTGGCATGCTAAGTATCAAGTTTTGCTTGCTGTTCTGACTGATCAA |
| CATCTCATGTCGTTTCAGTTGGCGAAACAAAAGCTGAAGTGTGTTCTTAACACTTCCTAG |
| CATGAAGTCAGACAA |
| >GS_A4.1 (*Symbiodinium*) |
| AACCAATGGCCTCTTGAACGTGCATTGCGCTCTTGGGATGCCTGAGAGCATGTCTGCTTC |
| AGTGCTTCTACTTTCATTTTCTGCTGCTCTTGTTATCGGGAGCAGTGTTGCTGCATGCTT |
| CTGCAAAAGGCACTGGCATGCTAAGTATCAAGTTTTGCTTGCTGTTCTGACTGATCAACA |
| TCTCATGTCGTTTCAGTTGGCGAAACAAAAGCTGAAGTGTGTTCTTAACACTTCCTAGCA |
| TGAAGTCAGACAA |
| >GS_A4.2 (*Symbiodinium*) |
| AACCAATGGCCTCTTGAACGTGCGCTCTTGGGATATGCCTGAGAGCATGTCTGCTTCAGT |
| GCTTCTACTTTCATTTTCTGCTGCTCTTGTTATCGGGAGCAGTGTTGCTGCATGCTTCTG |
| CAAAAGGCACTGGCATGCTAAGTATCAAGTTTTGCTTGCTGTTCTGACTGATCAACATCT |
| CATGTCGTTTCAGTTGGCGAAACAAAAGCTGAAGTGTGTTCTTAACACTTCCTAGCATGA |
| AGTCAGACAA |
| >GS_A4.3 (*Symbiodinium*) |
| AACCAATGGCCTCTTGAACGTGCATTGCGCTCTTGGGATATGCCTGACAGCATGTCTGCT |
| TCAGTGCTTCTACTTTCATTTTCTGCTGCTCTTGTTATCGGGAGCAGTGTTGCTGCATGC |
| TTCTGCAAAAGGCACTGGCATGCTAAGTATCAAGTTTTGCTTGCTGTTCTGACTGATCAA |
| CATCTCATGTCGTTTCAGTTGGCGAAACAAAAAAGCTGAAGTGTGTTCTTAACACTTCCT |
| AGCATGAATTCAGACAA |
| >GS_A5 (*Symbiodinium*) |
| AACCAATGGCCTCTTGAACGTGCATTGCGCTCTTGGGATATGCCTGAGAGCATGTCTGCT |
| TCAGTGCTTCTACTTTCATTTTCTGCTGCTCTTGTTATCAGGAGCAGTGCTGCTGCATGC |
| TTCTGCAATGGGCACTGGCATGCTAAGTACCAAGTTTTGCTTGCTGTTGTGACTGATCAA |
| CATCTCATGTCGTTTCAGTTGGCGAAACAAAGGCTTGTGTGTTCCAACACTTCCTAGCAT |
| GAAGTCAGACAA |
| >GS_A6 (*Symbiodinium*) |
| AACCAATGGCCTCTTGAACGTGCATTGCGCTCTTGGGATATGCCTGAGAGCATGTCTGCT |
| TCAGTGCTTCTACTTTCTTTTCTGCTGCTCTTGTTATCAGGAGCAGTGCTGCTGCATGCT |
| TCTGCAATTGGCACTGGCATGCTAAGTACCAAGTTTCGCTCGCTGTTGTGACTGATCAAC |
| ATCTCATGTCGTTTCAGTTGGCGAAACAAAGGCTTGTGTGTTCCAACACTTCCTAGCATG |
| AAGTCAGACAA |
| >GS_A7 (*Symbiodinium*) |
| AACCAATGGCCTCTTGAACGTGCATTGCGCTCTTGGGATATGCCTGAGAGCATGTCTGCT |
| TCAGTGCTTCTACTTTCTTTTCTGCTGCTCTTGTTATCATGAGCAGTGCTGCTGCATGCT |
| TCTGCAATTGGCACTGGCATGCTAAATACCAAGTTTTGCTTGCTGTTGTGACTGATCAAC |
| ATCTCATGTCGTTTCAGTTGGCGAAACAAAGGCTTGTGTGTTCCAACACTTCCTAGCATG |
| AAGTCAGATAA |
| >GS_A8 (*Symbiodinium*) |
| AACCAATGGCCTCTTGAACGTGCATTGCGCTCTTGAGATATGCCTGAGAGCATGTCTGCT |
| TCAGTGCTTCTACTTTCTTTTCTGCTGCTCTTGTTATCATGAGCAGTGCTGCTGCATGCT |
| TCTGCAATTGGCACTGGCATGCTAAGTATCAAGTTTTGCTTGCTGTTGTGACTGATCAAC |
| ATCTCATGTCGTTTCAGTTGGCGAAACAAAGGCTTGTGTGTTCCAACACTTCCTAGCATG |
| AAGTCAGATAA |
| >GS_A9 (*Symbiodinium*) |
| AACCAATGGCCTCTTGAACGTGCATTGTGCTCTTGGGATATGCCTGAGAGCATGTCTGCT |
| TCAGTGCTTCTACTTTCATTTTCTGCTGGTCTTGTTATCAGGAGCAGTGTTGCTGCGTGC |
| TTCTGCAAGTGGCACTGGCATGCTAAATATCAAGTTTTGCTTGCTGTTGTGACTGATCAA |
| CATCTCATGTCGTTTCAGTTGGCGAAACAAAAGCTCATGTGTGTTCTTAACACTTCCTAG |
| CATGAAGTCAGACAA |
| >GS_A10 (*Symbiodinium*) |
| AACCAATGGCCTCTTGAACGTGCATTGCGCTCTTGGGATATGCCTGAGAGCATGTCTGCT |
| TCAGTGCTTCTTCTTTCATTTTCTGCTGCTCTTGTTATCCGGAGCAGTGTTGCTGCAAGC |
| TTCTGCAAGTGGCACTGGCATGCTAAATATCGTTTTGCTTGCTGTTGTGACTGATCAACA |
| TCTCATGTCGTTTCAGTTGGCGAAACAAAAGCTCATGTGTGTTCTTAACACTTCCTAGCA |
| TGAAGTCAGACAA |
| >GS_A11 (*Symbiodinium*) |
| AACCAATGGCCTCTTGAACGTGCATTGCGCTCTTGGGATATGCCTGAGAGCATGTCTGCT |
| TCAGTGCTTCTACTATCTTTTCTGCTGCTCTTGTTGTCAGGAGCAGTGCTGCTGCATGCT |
| TCTGCAATTGGCACTGGCATGCTAAGTACCAAGTTTCGCTTGCTGTTGTGACAGATCAAC |
| ATCTCATGTCGTTTCAGTTGATGAAACAAGGCTTGCGTGTTCCAACACTTCCTAGCATGA |
| AGTCAGACAA |
| >GS_A12 (*Symbiodinium*) |
| AACCAATGGCCTCTTGAACGTGTATTGCGCTCTTGGGATATGCCTGAGAGCATGTCTGCT |
| TCAGTGCTTCTACTTTCATTTTCTGCTGCTCTTGCTATCAGGAGCAGTGTTGCTGCATGC |
| TTCTGCAAGCGGCACTGGCATGCTAAATATCAAGTTTTGCTTGCTGTTGTGACTGATCAA |
| CATCTCATGTCGTTTCAGTTGGCGAAACAAAAGCTCATGTGTGTTCTTAACACTTCCTAG |
| CATGAAGTCAGACAA |
| >LJ_A14 (*Symbiodinium*) |
| TTGTCTGACTTCATGCTAGGAAGTGTTAAGAACACACTTCAGCTTTTGTTTCGCCAACTGAGACGACATGAGATGTTGATCAGTCAGAACAGCAAGCAAAACTTGATACTTAGCATGCCAGTGCCTTTTGCAGAAGCATGCAGCAACACTGCTCCCGATAACAAGAGCAGCAGAGAATGAAAGTAGAAGCACTGAAGCAGACATGCTCTCAGGCATATCCCAAGAGCGCAATGCACGTTCAAGAGGCCATTGGTTCA |
| >GS_A15 (*Symbiodinium*) |
| AACCAATGGCCTCTTGAACGTGCATTGCGCTCTTGGGATATGCTTGAGAGCATGTCTGCT |
| TCAGTGCTTCTACTTTCTTCTCTGGTGCTCTTGTTATCATGAGCAGTGCTGCTGCATGCT |
| TCTGCAATTGGCACTGGCATGCTAAGTACCAAGTTTTGCTTGCTGTTGTGACTGATCAAC |
| ATCTCATGTCGTTTCAGTTAGCGAAACAAAGGCTTGTGTGTTCCAACACTTCCTAGCATG |
| AAGTCAGATAA |
| >GS_A15a (*Symbiodinium*) |
| AACCAATGGCCTCTTGAACGTGCATTGCGCTCTTGGGATATGCTTGAGAGCATGTCTGCT |
| TCAGTGCTTCTACTTTCTTCTCTGGTGCTCTTGTTATCATGAGCAGTGCTGCTGCATGCT |
| TCTGCAATTGGCACTGGCATGCTAAGTACCAAGTTTTGCTTGCTGTTGTGACTGATCAAC |
| ATCTCATGTCGTTTCAGTTGGCGAAACAAAGGCTTGTGTGTTCCAACACTTCCTAGCATG |
| AAGTCAGATAA |
| >GS_A15b (*Symbiodinium*) |
| AATCAATGGCCTCTTGAACGTGCATTGCGCTCTTGGGATATGCTTGAGAGCATGTCTGCT |
| TCAGTGCTTCTACTTTCTTCTCTGGTGCTCTTGTTATCATGAGCAGTGCTGCTGCATGCT |
| TCTGCAATTGGCACTGGCATGCTAAGTACCAAGTTTTGCTTGCTGTTGTGACTGATCAAC |
| ATCTCATGTCGTTTCAGTTAGCGAAACAAAGGCTTGTGTGTTCCAACACTTCCTAGCATG |
| AAGTCAGATAA |
| >GS_A15c (*Symbiodinium*) |
| AACCAATGGCCTCTTGAACGTGCATTGCGCTCTTGGGATATGCTTGAGAGCATGTCTGCT |
| TCAGTGCTTCTACTTTCTTCTCTGGTGCTCTTGTTATCATGAGCAGTGCTGCTGCATGCT |
| TCTGCAATTGGCACTGGCATGCTAAGTACCAAGTTTTGCTTGCTGTTGTGACTGATCAAC |
| ATCTCATGTCGTTTCAGTTAGCGAAACAAAGGCTTGTGTGCTCCAACACTTCCTAGCATG |
| AAGTCAGATAA |
| >GS_A16 (*Symbiodinium*) |
| AACCAATGGCCTCTTGAACGTGCATTGCGCTCTTGGGATATGCCTGAGAGCATGTCTGCT |
| TCAGTGCTTCTACTTTCTTTTCTGCTGCTCTTGTTATCATGAGCAGTGCTGCTGCATGCT |
| TCTGCAATTGGCACTGGCATGCTAGGTACCAAGTTTTGCTTGCTGTTGTGACTGATCAAC |
| ATCTCATGTCGTTTCAGTTGGCGAAACAAAGGCTTGTGTGTTCCAACACTTCCTAGCATG |
| AAGTCAGATAA |
| >ST_FREE(A) (*Symbiodinium*) |
| AACCAATGGCCTCTTGAACGTGCATTGCGCTCTTGGGATATGCCTGAGAGCATGTCTGCTTCAGTGCTTCTACTTTTCCTATTCCTGCTGCTCCTTTCAAGGGGTGGTGCTGTTGTGTGCTACTGCATACTTTGCATTGGCATGCTCAGTATTAAGCATTGCCCACTGGGTTGACTGATCAACGTTTCATGTCTTTTTCAGTCGGGCAACTCAACACCTGGTGTCTTGAACACTTCCTAGCATGAAGTCAGACAAGTGA |
| >GS_B1 (*Breviolum*) |
| AACCGATGGCCTCCTGAACGCGCATTGCGCTCTCGGGATTTCCTGAGAGCAGGTCTGCTT |
| CAGTGCTTAGCATTATCTACCTGTGCTTGCAAGCAGCATGTATGTCTGCATTGCTGCTTC |
| GCTTTCCAACAAGTCATCGATCGCTTTTGTGTTCGTAAATGGCTTGTTTGCTGCCTGGCC |
| CATGCGCCAAGCTTGAGCGTACTGTTGTTCCAAGCTTTGCTTGCATCGTGCAGCTCAAGC |
| GCGCAGCTGTCGGGATGCTGATGCATGCCCTTAGCATGA |
| >GS_B1a (*Breviolum*) |
| AACCGATGGCCTCCTGAACGCGCATTGCGCTCTCGGGATTTCCTGAGAGCAGGTCTGCTT |
| CAGTGCTTAGCATTATCTACCTGTGCTTGCAAGCAGCATGTATGTCTGCATTGCTGCTTC |
| GCTTTCCAACAAGCCATCGATCGCTTTTGTGTTCGTAAATGGCTTGTTTGCTGCCTGGCC |
| CATGCGCCAAGCTTGAGCGTACTGTTGTTCCAAGCTTTGCTTGCATCGTGCAGCTCAAGC |
| GCGCAGCTGTCGGGATGCTGATGCATGCCCTTAGCATGA |
| >GS_B1b (*Breviolum*) |
| AACCGATGGCCTCCTGAACGCGCATTGCGCTCTCGGGATTTCCTGAGAGCAGGTCTGCTT |
| CAGTGCTTAGCATTATCTACCTGTGCTTGCGAGCAGCGTATGTCTGCATTGCTGCTTCGC |
| TTTCCAACAAGTCATCGATCGCTTCGTAAATGGCTTGTTTGCTGCCTGGCCCACGTGCCA |
| AGCTTGAGCGTACTGTTGTTCCAAGCTTTGCTTGCATCGTGCAGCTCAAGCGCGCAGCTG |
| TCGGGATGCTGATGCATGCCCTTAGCATGA |
| >GS_B1d (*Breviolum*) |
| AACCGATGGCCTCCTGAACGCGCATTGCGCTCTCGGGATTTCCTGAGAGCAGGTCTGCTT |
| CAGTGCTTAGCATTATCTACCTGTGCTTGCAAGCAGCATGTATGTCTGCATTGCTGCTTC |
| GCTTTCCAACAAGTCATTGATCGCTTTTGTGTTCGTAAATGGCTTGTTTGCTGCCTGGCC |
| CATGCGCCAAGCTTGAGCGTACTGTTGTTCCAAGCTTTGCTTGCATCGTGCAGCTCAAGC |
| GCGCAGCTGTCGGGATGCTGATGCATGCCCTTAGCATGA |
| >GS_B1e (*Breviolum*) |
| AACCGATGGCCTCCTGAACGCGCATTGCGCTCTCGGGATTTCCTGAGAGCAGGTCTGCTT |
| CAGTGCTTAGCATTATCTACCTGTGCTTGCAAGCAGCATGTATGTCTGCATTGCTGCTTC |
| GCTTTCCAACAAGTCATCAATCGCTTTTGTGTTCGTAAATGGCTTGTTTGCTGCCTGGCC |
| CATGCGCCAAGCTTGAGCGTACTGTTGTTCCAAGCTTTGCTTGCATCGTGCAGCTCAAGC |
| GCGCAGCTGTCGGGATGCTGATGCATGCCCTTAGCATGA |
| >GS_B1g (*Breviolum*) |
| AACCGATGGCCTCCTGAACGCGCATTGCGCTCTCGGGATTTCCTGAGAGCAGGTCTGCTT |
| CAGTGCTTAGCATTATCTACCTGTGCTTGCAAGCAGCATGTATGTCTGCATTGCTGCTTC |
| GCTTTCCAACAAGTCATCGACCGCTTTTGTGTTCGTAAATGGCTTGTTTGCTGCCTGGCC |
| CCACGCGCCAAGCTTGAGCGTACTGTTGTTCCAAGCTTTGCTTGCATCGTGCAGCTCAAG |
| CGCGCAGCTGTCGGGATGCTGATGCATGCCCTTAGCATGA |
| >GS_B1i (*Breviolum*) |
| TTGTCTGACTTCATGCTAAGGGCATGCATCAGCATCCCGACAGCTGCGCGCTTGAGCTGCACGATGCAAGCAAAGCTTGGAACAACAGTACGCTCAAGCTTGGCGCATGGGCCAGGCAGCAAACAAGCCATTTACGAACACAAAAGCGATCGATGACGTGTTGGAAAGCGAAGCAGCAATGCAGACATACATGCTGCTTGCAAGCACAGGTAGATAATGCTAAGCACTGAAGCAGACCTGCTCTCAGGAAATCCCGAGAGCGCAATGCGCGTTCAGGAGGCCATCGGTT |
| >GS_B1j (*Breviolum*) |
| AACCGATGGCCTCCTGAACGCGCATTGCGCTCTAGGGATTTCCTGAGAGCAGGTCTGCTT |
| CAGTGCTTAGCATTATCTACCTGTGCTTGCAAGCAGCATGTATGTCTGCATTGCTGCTTC |
| GCTTTCCAACAAGTCATCGATCGCTTTTGTGTTCGTAAATGGCTTGTTTGCTGCCTGGCC |
| CATGCGCCAAGCTTGAGCGTACTGTTGTTCCAAGCTTTGCTTGCATCGTGCAGCTCAAGC |
| GTGCAGCTGTCGGGATGCTGATGCATGCCCTTAGCATGA |
| >GS_B1k (*Breviolum*) |
| AACCGATGGCCTCATCGCTTTGTGTTCGTAAATGGCTTGTTTGCTGCCTGGCCCATGCGC |
| CAAGCTTGAGCATACTGTTGTTCCAAGCTTTGCTTGCATCGTCCAGCTCAAGCGCGCAGC |
| TGTCGGGATGCTGATGCATGCCCTTAGCATGA |
| >GS_B1L (*Breviolum*) |
| AACCGATGGCCTCCTGAACGCGCATTGCGCTCTCGGGATTTCCTGAGAGCAGGTCTGCTT |
| CAGTGCTTAGCATTATCTACCTGTGCTTGCAAGCAGCATGTATGTCTGCATTGCTGCTTC |
| GCTTTCCAACAAGTCATCGATCGCTTTTGTGTTCGTAAATGGCTTGTTTGCTGCCTGGCC |
| CACGCGCCAAGCTTGAGCGTACTGTTGTTCCAAGCTTTGCTTGCATCGTGCAGCTCAAGC |
| GCGCAGCTGTCGGGATGCTGATGCATGCCCTTAGCATGA |
| >GS_B1m (*Breviolum*) |
| AACCGATGGCCTCCTGAACGCGCATTGCGCTCTCGGGATTTCCTGAGAGCAGGTCTGCTT |
| CAGTGCTTAGCATTATCTACCTGTGCTTGCAAGCAGCATGTATGTCTGCATTGCTGCTTC |
| GCTTTCCGACAAGTCATCGATCGCTTTTGTGTTCGTAAATGGCTTGTTTGCTGCCTGGCC |
| CATGCGCCAAGCTTGAGCGTACTGTTGTTCCAAGCTTTGCTTGCATCGTGCAGCTCAAGC |
| GCGCAGCTGTCGGGATGCTGATGCATGCCCTTAGCATGA |
| >GS_B2 (*Breviolum*) |
| AACCGATGGCCTCCTGAACGCGCATTGCGCTCTCGGGATTTCCTGAGAGCAGGTCTGCTT |
| CAGTGCTTAGCATTATCTACCTGTGCTTGCAAGCAGCATGTCTACACTGCTGCTTTGCTT |
| TCCAACAAGTCATCGATCGCGTTTGTGTTCGTAAATGGCTTGTTTGCTGCCTGGCCCATG |
| CGCCAAGCTTGAGCGTACTGTTGTTCCAAGCTTAGCTTGCATCGTACAGCTCAAGCGCGC |
| AGCTGTTGGGATGCTGATGCATGCCCTTAGCATGA |
| >GS_B3 (*Breviolum*) |
| AACCGATGGCCTCCTGAACGCGCATTGCGCTCTCGGGATTTCCTGAGAGCAGGTCTGCTT |
| CAGTGCTTAGCATTATCTACCTCTGCTTGCAAGCAGCATGTCTACACTGCTGCTTTGCTT |
| TCCAAGAAGTCATCGATCGCGTTTGTGTTCGTAAATGGCTTGTTTGCTGCCTGGCCCATG |
| CGCCAAGTTTGAGCGTACTGTTGTTCCAAGCTTAGCTTGCATCGTACAGCTCAAGCGCGC |
| AGCTGTTGGGATGCTGATGCATGCCCTTAGCATGA |
| >GS_B4 (*Breviolum*) |
| AACCGATGGCCTCCTGAACGCGCATTGCGCTCTCGGGATTTCCTGAGAGCACGTCTGCTT |
| CAGTGCTTAGCCTTATTTACTTGTGCTTGCAAGCAGCATGTGTGCACTGCTGCTTTGCTT |
| TCCAACAAGTCACTGGCGTGTAGTGGCTTGTTTGCTGCCTGGCCCATGCGCCAAGCTTGA |
| GCGTACTGTTGTTCCAAGCTTATGCCTGCCTGGTGTAGCTTGCATCATGCTGCTCAAGCG |
| CGCAGCTGTCGGGATGCTGGTGCATGCCCTTAGCATGA |
| >GS_B5 (*Breviolum*) |
| AACCGATGGCCTCCTGAACGCGCATTGCGCTCTCGGGATTTCCTGAGAGCAGGTCTGCTT |
| CAGTGCTTAGCATTATCTACCTGTGCTTGCAAGCAGCATGTATGTCTGCACTGCTGCTTC |
| GCTTTCCAACAAGTCATCGATCGCTTTTGTGTTCGTAAATGGCTTGTTTGCTGCCTGGCC |
| CATGCGCCAAGCTTGAGCGTACTGTTGTTCCAAGCTTTGCTTGCATCGTGCAGCTCAAGC |
| GCGCAGCTGTCGGGATGCTGATGCATGCCCTTAGCATGA |
| >GS_B5a (*Breviolum*) |
| AACCGATAGCCTCCTGAACGCGCATTGCGCTCTCGGGATTTCCTGAGAGCAGGTCTGCTT |
| CAGTGCTTAGCATTATCTACCTGTGCTTGCAAGCAGCATGTATGTCTGCACTGCTGCTTC |
| GCTTTCCAACAAGTCATCGATCGCTTTTGTGTTCGTAAATGGCTTGTTTGCTGCCTGGCC |
| CATGCGCCAAGCTTGAGCGTACTGTTGTTCCAAGCTTTGCTTGCATCGTGCAGCTCAAGC |
| GCGCAGCTGTCGGGATGCTGATGCATGCCCTTAGCATGA |
| >GS_B6 (*Breviolum*) |
| AACCGATGGCCTCCTGAACGCGCATTGCGCTCTCGGGATTTCCTGAGAGCAGGTCTGCTT |
| CAGTGCTTAGCATTATCTACCTGTGCTTGCAAGCAGCATGTCTACACTGCTGCTTCGCTT |
| TCCAACAAGTCATCGATCGCGTTTGTGTTCGTAAATGGCTTGTTTGCTGCCTGGCCCATG |
| CGCCAAGCTTGAGCGTACTGTTGTTCCAAGCTTAGCTTGCTTGCATCGTACAGCTCAAGC |
| GCGCAGCTGTTGGGATGCTGATGCATGCCCTTTAGCATGA |
| >GS_B7 (*Breviolum*) |
| AACCGATGGCCTCCTGAACGCGCATTGCGCTCTCGGGATTTCCTGAGAGCAGGTCTGCTT |
| CAGTGCTTAGCATTCTCTACCTGTGCTTGCAAGCAGCATGTATGTCTGCATTGCTGCTTC |
| GCTTTCCAACAAGTCATCGATCGCTTTTGTGTTCGTAAATGGCTTGTTTGCTGCCTGGCC |
| CATGCGCCAAGCTTGAGCGTACTGTTGTTCCAAGCTTTGCTTGCATCGTGCAGCTCAAGC |
| GCGCAGCTGTCGGGATGCTGATGCATGCCCTTAGCATGA |
| >GS_B8 (*Breviolum*) |
| AACCGATGGCCTCCTGAACGCGCATTGCGCTCTCGGGATTTCCTGAGAGCAGGTCTGCTT |
| CAGTGCTTAGCATTATCTACCTGTGCTTGCAAGCAGCATGTATGTCTGCATTGCTGCTTC |
| GCTTTCCCAACAAGTCATCGATCGCTTTTGTGTTCGTAAATGGCTTGTTTGCTGCCTGGC |
| CCACGCGCCAAGCTTGAGCGTACTGTTGTTCCAAGCTTTGCTTGCATCGTGCAGCTCAAG |
| CGCGCAGCTGTCGGGATGCTGATGCATGCCCTTAGCATGA |
| >GS_B9 (*Breviolum*) |
| AACCGATGGCCTCCTGAACGTGCATTGCGCTCTCGGGGTTTCCTGAGAGCAGGTCTGCTT |
| CAGTGCTTAGCATTATCTACCTGTGCTTGCAAGCAGCATGTCTACACTGCTGCTTCGCTT |
| TCCAACAAGTCATCGATCGCGTTTGTGTTCGTAAATGGCTTGTTTGCTGCCTGGCCCATG |
| CGCCAAGCTTGAGCGTACTGTTGTTCCAAGCTTAGCTTGCATCGTATAGCTCAAGCGCGC |
| AGCTGTTGGGATGCTGATGCATGCCCTTAGCATGA |
| >GS_B10 (*Breviolum*) |
| AACCGATGGCCTCCTGAACGCGCATTGCGCTCTCGGGATTTCCTGAGAGCAGGTCTGCTT |
| TAGTGCTTAGCATTATCTACCTGTGCTTGCAAGCAGCATGTATGTCTGCATTGCTGCTTC |
| GCTTTCCAACAAGTCATCGATCGCTTTTGTGTTCGTAAATGGCTTGTTTGCTGCCTGGCC |
| CATGCGCCAAGCTTGAGCGTGCTGTTGTTCCAAGCTTTGCTTGCATCGTGCAGCTCAAGC |
| GCGCAGCTGTCGGGATGCTGATGCATGCCCTTAGCATGA |
| >GS_B11 (*Breviolum*) |
| AACCGATGGCCTCCTGAACGCGCATTGCGCTCTCGGGATTTCCTGAGAGCAGGTCTGCTT |
| CAGTGCTTAGCATTATCTACCTGTGCTTGCAAGCAGCATGTATGTCTGCACACTGCTGTT |
| TCGCTTTCCAACAAGTCATCGATCGCTTTTGTGTTCGTAAATGGCTTGTTCGCTGCCTGG |
| CCCATGCGCCAAGCTTGAGCGTACTGTTGTTCCAAGCTTTGCTTGCATCGTGCTGCTCAA |
| GCGCGCAGCTGTCGGGATGCTGATGCATGCCCTTAGCATGA |
| >GS_B13 (*Breviolum*) |
| TGAACGCGCATTGCGCTCTCGGGATTTCCTGAGAGCAGGTCTGCTTCAGTGCTTAGCATT |
| CTCTACCTGTGCGTGCAAGCAGCATGTATGTCTGCATTGCTGCTTCGCTTTCCAACAAGT |
| CATCGATCGCTTTTGTGTTCGTAAATGGCTTGTTTGCTGCCTGGCCATGCGCCAAGCTTG |
| AGCGTACTGTTGTTCCAAGCTTTGCTTGCATCGTGCAGCTCAAGCGCGCAGCTGTCGGGA |
| TGCTGATGCATGCCCTTAGCATGA |
| >GS_B14 (*Breviolum*) |
| AACCGATGGACCTCCTGAACGCGCACTGCGCTCTCGGGATTTCCTGAGAGCAGGTCTGCT |
| TCAGTGCTTAGCATTATCTACCTGTGCTTGCAAGCAGCATGTATGTCTGCATTGCTGCTT |
| CGCTTTCCAACAAGTCATCGATCGCTTTTGTGTTCGTAAATGGCTTGTTTGCTGCCTGGC |
| CCATGCGCCAAGCTTGAGCGTACTGTTGTTCCAAGCTTTGCTTGCATCGTGCAGCTCAAG |
| CGCGCAGCTGTCGGGATGCTGATGCATGCCCTTAGCATGA |
| >GS_B15 (*Breviolum*) |
| AACCGATGGCCTCCTGAACGCGCAKTGCGCTCTCGGGATTTCCTGAGAGCAGGTCTGCTT |
| CAGTGCTTAGCATTATCTACCTGTGCTTGCAAGCAGCGTGTCTACACTGCTGCTTCGCTT |
| TCCAACAAGTCATCGATCGCGTTTGTGTTCGTAAGTGGCTTGTTTGCTGCCTGGCCCATG |
| CGCCAAGCTTGAGCGTACTGTTGTTCCAAGCTTTGCTTGCATCGTACAGCTCAAGCGCGC |
| AGCTGTTGGGATGCTGATGCATGCCCT |
| >GS_B17 (*Breviolum*) |
| AACCGATGGCCTCCTGAACGCGCATTGCGCTCTCGGGATTTCCTGAGAGCAGGTCTGCTT |
| CAGTGCCTAGCATTATCTACCTGTGCTTGCAAGCAGCATGTATGTCTGCATTGCTGCTTC |
| GCTTTCCAACAAGTCATCGATCGCTTTTGTGTTCGTAAATGGCTTGTTTGCTGCCTGGCC |
| CATGCGCCAAGCTTGAGCGTACTGTTGTTCCAAGCCTTGCTTGGATCGTGCAGCTCAAGC |
| GCGCAGCTGTCGGGATGCTGATGCATGCCCTTAGCATGA |
| >GS_B18 (*Breviolum*) |
| AACCGATGGCCTCCTGAACGCGCATTGCGCTCTCGGGATTTCCTGAGAGCAGGTCTGCTT |
| CAGTGCTTAGCATTATCTACCTGTGCTTGCAAGCAGCATGTCTACACTGCTGCTTCGCTT |
| TCCAACAAGTCATCGATCGCATTTGTGTTCGTAAATGGCTTGTTTGCTGCCTGGCCCATG |
| CGCCAAGTTTGAGCGTACTGTTGTTCCAAGCTTAGCTTGCATCGTACAGCTCAAGCGCGC |
| AGCTGTTGGGATGCTGATGCATGCCCTTAGCATGA |
| >ST_B18a_FB4A (*Breviolum*) |
| AACCGATGGCCTCCTGAACGCGCATTGCGCTCTCGGGATTTCCTGAGAGCAGGTCTGCTTCAGTGCTTAGCATTATCTACCTGTGCTTGCAAGCAGCATATCTACACTGCTGCTTCGCTTTCCAACAAGTCATCGATCGCATTTGTGTTCGTAAATGGCTTGTTTGCTGCCTGGCCCATGCGCCAAGTTTGAGCGTACTGTTGTTCCAAGCTTAGCTTGCATCGTACAGCTCAAGCGCGCAGCTGTTGGGATGCTGATGCATGCCCTTAGCATGAAGTCAGACAAGAGA |
| >GS_B19 (*Breviolum*) |
| AACCGATGGCCTCCTGAACGCGCATTGCGCTCTCGGGATTTCCTGAGAGCAGGTCTGCTT |
| CAGTGCTTAGCATTATCTACCTGTGCTTGCAAGCAGCATGTCTACACTGCTGCTTCGCTT |
| TCCAACAAGTCATCGATCGCGTTTGTGTTCGTAAATGGCTTGTTTGCTGCCTGGCCCATG |
| CGCCAAGCTTGAGCGTACTGTTGTTCCAAGCTTAGCTTGCATCGTACAGCTCAAGCGCGC |
| AGCTGTTGGGATGCTGATGCATGCCCTTAGCATGA |
| >GS_B20 (*Breviolum*) |
| AACCGATGGCCTCCTGAACGCGCATTGCGCTCTCGGGATTTCCTGAGAGCAGGTCTGCTT |
| CAGTGCTTAGCATTATCTACCTGTGCTTGCAAGCAGCATGTATGTCTGCATTGCTGCTTC |
| GCTTTCCAACAAGTCATCGATCGCTTTTGTGCGATCGTAAATGGCTTGTTTGCTGCCTGG |
| CCCATGCGCCAAGCTTGAGCGTACTGTTGTTCCAAGCTTTGCTTGCATCGTGCAGCTCAA |
| GCGCGCAGCGTCGGGATGCTGATGCATGCCCTTAGCATGA |
| >GS_B21=B38 (*Breviolum*) |
| AACCGATGGCCTCCTGAACGCGCATTGCGCTCTCGGGATTTCCTGAGAGCAGGTCTGCTT |
| CAGTGCTTAGCATTATCTACCTGTGCTTGCAAGCAGCATGTCTACACTGCTGCTTCGCTT |
| TCCAACAAATCATCGCGTTTGTGTTCGTAAATGGCTTGTTTGCTGCCTGGCCCATGCGCC |
| AAGCTTGAGCGTAGTGTTGTTCCAAGCTTAGCTTGCATCGTACAGCTCAAGCGCGCAGCT |
| GTTGGGATGCTGATGCATGCCCTTAGCATGA |
| >LJ_B23 (*Breviolum*) |
| TTGTCTGACTTCATGCTAAGGGCATGCATCAGCATCCCAACAGCTGCGCGCTTGAGCTGTACGATGCAAGCTAAGCTTGGAACAACAGTACGCTCAAACTTGGCGCATGGGCCAGGCAGCAAACAAGCCATTTACGAACACACACACGATCGATGACTTGTTGGAAAGCGAAGCAGCAGTGTAGACATGCTGCTTGCAAGCAGAGGTAGATAAATGCTAAGCACTGAAGCAGACCTGCTCTCAGGGAATCCCGAGAGCGCAATGCGCGTTCAGGAGCCCATCGATT |
| >GS_B30 (*Breviolum*) |
| AATCGATGGGCTCCTGAACGCGCATTGCGCTCTCGGGATTCCCTGAGAGCAGGTCTGCTT |
| CAGTGCTTAGCATTTATCTACCTCTGCTTGCAAGCAGCATGTCTACACTGCTGCTTCGGT |
| TTCCAACAAGTCATCGATCGTGTGTGTATTCGTAAATGGCTTGTTTGCTGCCTGGCCCAT |
| GCGCCAAGTTTGAGCGTACTGTTGTTCCAAGCTTAGCTTGCATCGTACAGCTCAAGCGCG |
| CAGCTGTTGGGATGCTGATGCATGCCCTTAGCATGA |
| >GS_B31 (*Breviolum*) |
| AACCGATGGCCTCCTGAACGCGCATTGCGCTCTCGGGATTTCCTGAGAGCAGGTCTGCTT |
| CAGTGCTTAGCATTATCTACCTGTGCTTGCAAGCAGCATGCATGTCTGCATTGCTGCTTC |
| GCTTTCCAACAAGTCATCGATCGCTTTTGTGTTCGTAAATGGCTTGTTTGCTGCCTGGCC |
| CATGCGCCAAGCTTGAGCGTACTGTTGTTCCAAGCTTTGCTTGCATCGTGCAGCTCAAGC |
| GCGCAGCTGTCGGGATGCTGATGCATGCCCTTAGCATGA |
| >GS_B31a (*Breviolum*) |
| AACCGATGGCCTCCTGAACGCGCATTGCGCTCTCGGGATTTCCTGAGAGCAGGTCTGCTT |
| CAGTGCTTAGCATTATCTACCTGTGCTTGCAAGCAGCATGCATGTCTGCATTGCTGCTTC |
| GCTTTCCAACAAGTCATCGATCGCTTTTGTGTTCGTAAATGGCTTGTTTGCTGCCTGGCC |
| CATGCGCCAAGCTTGAGCGTACTGTTGTTCCAAGCTTTGCTTGCATCGTGCACCTCAAGC |
| GCGCAGCTGTCGGGATGCTGATGCATGCCCTTAGCATGA |
| >GS_B32 (*Breviolum*) |
| AACCGATGGCCTCCTGAACGCGCATTGCGCTCTCGGGATTTCCTGAGAGCAGGTCTGCTT |
| CAGTGCTTAGCATTATCTACCTGTGCTTGCAAGCAGCATGCATGTCTGCATTGCTGCTTC |
| GCTTTCCAACAAGTCATCGATCGCTTTTGTGTTCGTAAATGGCTTGTTTGCTGCCTGGCC |
| CATGCGCCAAGCTTGAGCGTACTATTGTTCCAAGCTTTGCTTGCATCGTGCAGCTCAAGC |
| GCGCAGCTGTCGGGATGCTGATGCATGCCCTTAGCATGA |
| >LJ_B33 (*Breviolum*) |
| TTGTCTGACTTCATGCTAAGGGCATGCATCAGCATCCCAACAGCTGCGCGCTTGAGCTGTACAATGCAAGCTAAGCTTGGAACAACAGTACGCTCAAGCTTGGCGCATGGGCCAGGCAGCAAACAAGCCATTTACGAACACAAACGCGATCGATGACTTGTTGGAAAGCGAAGCAGCAGTGTGGACATGCTGCTTGCAAGCACAGGTAGATAATGCTAAGCACTGAAGCAGACCTGCTCTCAGGAAATCCCGAGAGCGCAATGCGCGTTCAGGAGGCCATCGGTT |
| >LJ_B36 (*Breviolum*) |
| TTGTCTGACTTCATGCTAAGGGCATGCATCAGCATCCCGACAGCTGCGCGCTTGAGCTGCACGATGCAAGCAAAGCTTGGAACAACAGTACGCTCAAGCTTGGCGCATGGGCCAGGCAGCAAACAAGCCATTTACGAACACAAAAGCGATCGATGACTTGTTGGAAAGTGAAGCAGCAATGCAGACATACATGCTGCTTGCAAGCACAGGTAGATAATGCTAAGCACTGAAGCAGACCTGCTCTCAGGAAATCCCGAGAGCGCAATGCGCGTTCAGGAGGCCATCGGTT |
| >GS_B37 (*Breviolum*) |
| AACCGATGGCCTCCTGAACGCGCATTGCGCTCTCGGGATTTCCTGAGAGCAGGTCTGCTT |
| CAGTGCTTTTAGCATTATCTACCTGTGCTTGCAAGCAGCATGTATGTCTGCATTGCTGCT |
| TCGCTTTCCAACAAGTCATCGATCGCTTTTGTGTTCGTAAATGGCTTGTTTGCTGCCTGG |
| CCCATGCGCCAAGCTTGAGCGTACTGTTGTTCCAAGCTTTGCTTGCATCGTGCAGCTCAA |
| GCGTGCAGCTGTCGGGATGCTGATGCATGCCCTTAGCATGA |
| >GS_B40 (*Breviolum*) |
| AACCGATGGCCTCCTGAACGCGCATTGCGCTCTCGGGATTTCCTGAGAGCAGGTCTGCTT |
| CAGTGCTTTTAGCATTTATCTACCTGTGCTTGCAAGCAGCGCTTCGCTTTCCAACAAGTC |
| ATCGATCGCGTTTGTGTTCGTAAGTGGCTTGTTTGCTGCCTGGCCCATGCGCCAAGCTTG |
| AGCGTACCGTACTGTTGTTCCAAGCTTTGCTTGCATCGTACAGCTCAAGCGCGCAGCTGT |
| TGGGATGCTGATGCATGCCCCTTAGCATGA |
| >ST_B02_71 (*Breviolum*) |
| GCATTGCGCTCTCGGGATTTCCTGAGAGCAGGGCTGATTCAGTGCTTAGCATTATCTACCTGTGCTTGCAAGCAGCATGTCTACACATGTCTACACTGCTGCTTCGCTTTCCAACAAGTCATCGATCGCGTTTGTGTTCGTAAATGGCTTGTTTGCTGTGCCTGGCCTTAGCTTGCATAGAAACCCCTGCAACTAACGTAGGGATCCCAATGCATGCCCTTAGCATGAAGTCAGACAAG |
| >GS_Cspa (*Cladocopium*) |
| AAGCAATGGCCTCCTGAACGTGCGTTGCACTCTTGGGATTTCCTGAGAGTATGTCTGCTT |
| CAGTGCTTAACTTGCCCCAACTTTGCAAGCAGGATGTGTTTCTGCCTTGCGTTCTTATGA |
| GCTATTGCCCTCTGAGCCAATGGCTTGTTAATTGCTTGGTTCTTGCAAAATGCTTTGCGC |
| GCTGTTATTCAAGTTTCTACCTTCGTGGTTTTACTTGAGTGACGCTGCTCATGCTTGCAA |
| CTGCTGGGATGCAGGTGCATGCCTCTAGCATGAAGTCAGACAA |
| >GS_Cspb (*Cladocopium*) |
| AACCAATGGCCTCCTGAACGTGCGTTGCACTCTTGGGATTTCCTGAGAGTATGTCTGCTT |
| CAGTGCTTAACTTGCCCCAACTTTGCAAGCAGGATGTGTTTCTGCCTTGCGTTCTTATGA |
| GCTATTGCCCTCTGAGCCAATGGCTTGTTAATTGCTTGGTTCTTGCAAAATGCTTTGCGC |
| GCTGTTATTCAATTTTCTACCTTCGTGGTTTTACTTGAGTGACGCTGCTCATGCTTGCAA |
| CCGCTGGGATGCAGGTGCATGCCTCTAGCATGAAGTCAGACAA |
| >GS_Cspd (*Cladocopium*) |
| AACCAATGGCCTCCTGAACGTGCGTTGCACTCTTGGGATTTCCTGAGAGTATGTCTGCTT |
| CAGCGCTTAACTTGCCCCAACTTTGCAAGCAGGATGTGTTTCTGCCTTGCGTTCTTATGA |
| GCTATTGCCCTCTGAGCCAATGGCTTGTTAATTGCTTGGTTCTTGCAAAATGCTTTGCGC |
| GCTGCTATTCAGGTTTCTACCTTCGTGGTTTTACTTGAGTGACGCTGCTCATGCTTGCAA |
| CCGCTGGGATGCAGGTGCATGCCTCTAGCATGAAGTCAGACAA |
| >GS_Cspe (*Cladocopium*) |
| AACCAATGGCCTCCTGAACGTGCGTTGCACTCTTGGGATTTCCTGAGAGTATGTCTGCTT |
| CAGTGCTTAACTTGCCCCAACTTTGCAAGCAGGATGTGTTTCTGCCTTGCGTTCTTATGA |
| GCTATTGCCCTCTGAGCCAATGGCTTGTTAATTGCTTGGTTCTTGCAAGATGCTTTGCGC |
| GCTGTTATTCAAGGTTCTACCTTCGTGGTTTTACTTGAGTGACGCTGCTCATGCTTGCAA |
| CCGCTGGGATGCAGGTGTGCATGCCTCTAGCATGAAGTCAGACAA |
| >GS_Cspf (*Cladocopium*) |
| AAGCAATGGCCTCCTGAACGTGTGTTGCACTCTTGGGATTTCCTGAGAGTATGTCTGCTT |
| CAGTGCTTAACTTGCCCCAACTTTGCAAGCAGGATGTGTTTCTGCCTTGCGTTCTTATGA |
| GCTATTGCCCTCTGAGCCAAGGGCTTGTTAATTGCTTGGTTCTTGCAAAATGCTTTGCGC |
| GCTGTTATTCAAGTTTCTACCTTTGTGGTTTTACTTGAGTGACGCTGCGCATGGTTGCAA |
| CCGCTGGGATGCAGGTGCATGCCTCTAGCATGAAGTCAGACAA |
| >GS_C1 (*Cladocopium*) |
| AACCAATGGCCTCCTGAACGTGCGTTGCACTCTTGGGATTTCCTGAGAGTATGTCTGCTT |
| CAGTGCTTAACTTGCCCCAACTTTGCAAGCAGGATGTGTTTCTGCCTTGCGTTCTTATGA |
| GCTATTGCCCTCTGAGCCAATGGCTTGTTAATTGCTTGGTTCTTGCAAAATGCTTTGCGC |
| GCTGTTATTCAGGTTTCTACCTTCGTGGTTTTACTTGAGTGACGCTGCTCATGCTTGCAA |
| CCGCTGGGATGCAGGTGCATGCCTCTAGCATGAAGTCAGACAA |
| >GS_C1ca=C1b=C1e (*Cladocopium*) |
| AACCAATGGCCTCCTGAACGTGCGTTGCACTCTTGGGATTTCCTGAGAGTATGTCTGCTT |
| CAGTGCTTAACTTGCCCCAACTTTGCAAGCAGATGTGTTTCTGCCTTGCGTTCTTATGAG |
| CTATTGCCCTCTGAGCCAATGGCTTGTTAATTGCTTGGTTCTTGCAAAATGCTTTGCGCG |
| CTGTTATTCAGGTTTCTACCTTCGTGGTTTTACTTGAGTGACGCTGCTCATGCTTGCAAC |
| CGCTGGGATGCAGGTGCATGCCTCTAGCATGAAGTCAGACAA |
| >GS_C1c.C45 (*Cladocopium*) |
| AACCAATGGCCTCCTGAACGTGCGTTGCACTCTTGGGATTTCCTGAGAGTATGTCTGCTT |
| CAGTGCTTAACTTGCCCCAACTTTGCAAGCAGGATGTGTTTCTGCCTTGCGTTCTTATGA |
| GCTATTGCCCTCTGAGCCAATGGCTTGTGAATTGCTTGGTTCTTGCAAAATGCTTTGCGC |
| GCTGTTATTCAGGTTTCTACCTTCGTGGTTTTACTTGAGTGACGCTGCTCATGCTTGCAA |
| CCGCTGGGATGCAGGTGCATGCCTCTAGCATGAAGTCAGACAA |
| >GS_C1d (*Cladocopium*) |
| AATCAATGGCCTCCTGAACGTGCGTTGCACTCTTGGGATTTCCTGAGAGTATGTCTGCTT |
| CAGTGCTTAACTTGCCCCAACTTTGCAAGCAGGATGTGTTTCTGCCTTGCGTTCTTATGA |
| GCTATTGCCCTCTGAGCCAATGGCTTGTGAATTGCTTGGTTCTTGCAAAATGCTTTGCGC |
| GCGGTTATTCAGGTTTCTACCTTCGTGGTTTTACTTGAGTGACGCTGCTCATGCTTGCAA |
| CCGCTGGGATGCAGGTGCATGCCTCTAGCATGAAGTCAGACAA |
| >GS_C1f (*Cladocopium*) |
| AACCAATGGCCTCCTGAACGTGCGTTGCACTCTTGGGATTTCCTGAGAGTATGTCTGCTT |
| CAGTGCTTAACTTGCCCCAACTTTGCAAGCAGGATGTGTTTCTGCCTTGCATTCTTATGA |
| GCTATTGCCCTCTGAGCCAATGGCTTGTTAATTGCTTGGTTCTTGCAAAATGCTTTGCGC |
| GCTGTTATTCAGGTTTCTACCTTCGTGGTTTTACTTGAGTGACGCTGCTCATGCTTGCAA |
| CCGCTGGGATGCAGGTGCATGCCTCTAGCATGAAGTCAGACAA |
| >GS_C1g (*Cladocopium*) |
| AACCAATGGCCTCCTGAACGTGCGTTGCACTCTTGGGATTTCCTGAGAGCATGTCTGCTT |
| CAGTGCTTAACTTGCCCCAACTTTGCAAGCAGGATGTGTTTCTGCCTTGCGTTCTTATGA |
| GCTATTGCCCTCTGAGCCAATGGCTTGTTAATTGCTTGGTTCTTGCAAAATGCTTTGCGC |
| GCTGTTATTCAGGTTTCTACCTTCGTGGTTTTACTTGAGTGACGCTGCTCATGCTTGCAA |
| CCGCTGGGATGCAGGTGCATGCCTCTAGCATGAAGTCAGACAA |
| >GS_C1h (*Cladocopium*) |
| AACCAATGGCCTCCTGAACGTGCGTTGCACTCTTGGGATTTCCTGAGAGTATGTCTGCTT |
| CAGTGCTTAACTTGCCCCAACTTTGCAAGCAGGATGTGTTTCTGCCTTGTGTTCTTATGA |
| GCTATTGCCCTCTGAGCCAATGGCTTGTTAATTGCTTGGTTCTTGCAAAATGCTTTGCGC |
| GCTGTTATTCAGGTTTCTACCTTCGTGGTTTTACTTGAGTGACGCTGCTCATGCTTGCAA |
| CCGCTGGGATGCAGGTGCATGCCTCTAGCATGAAGTCAGACAA |
| >GS_C1i (*Cladocopium*) |
| AACCAATGGCCTCCTGAACGTGCGTTGCACTCTTGGGATTTCCTGAGAGTATGTCTGCTT |
| CAGTGCTTAACTTGCCCCAACTTTGCAAGCAGGATGTGTTTCTGCCTTGCGTTCTTATGA |
| GCCATTGCCCTCTGAGCCAATGGCTTGTTAATTGCTTGGTTCTTGCAAAATGCTTTGCGC |
| GCTGTTATTCAGGTTTCTACCTTCGCGGTTTTACTTGAGTGACGCTGCTCATGCTTGCAA |
| CCGCTGGGATGCAGGTGCATGCCTCTAGCATGAAGTCAGACAA |
| >GS_C1j (*Cladocopium*) |
| AACCAATGGCCTCCTGAACGTGCGTTGCACTCTTGGGATTTCCTGAGAGTATGTCTGCTT |
| CAGTGCTTACCTTGCCCCAACTTTGCAAGCAGGATGTGTTTCTGCCTTGCGTTCTTATGA |
| GCTATTGCCCTCTGAGCCAATGGCTTGTTAATTGCTTGGTTCTTGCAAAATGCTTTGCGC |
| GCTGTTATTCAGGTTTCTACCTTCGTGGTTTTACTTGAGTGACGCTGCTCATGCTTGCAA |
| CCGCTGGGATGCAGGTGCATGCCTCTAGCATGAAGTCAGACAA |
| >GS_C1k (*Cladocopium*) |
| AACCAATGGCCTCCTGAACGTGCGTTGCACTCTTGGGATTTCCTGAGAGTATGTCTGCTT |
| CAGTGCTTAACTTGCCCCAACTTTGCAAGCAGGATGTGTTTCTGCCTTGCGTTCTTATGA |
| GCTATTGCCCTCTGAGCCAATGGCTTGTGAATTGCTTGGTTCTTGCAAAATGCTTTGCGC |
| GCTGTTATTCAGGTTTCTACCTTCGTGGTTTTACTTGAGTGACGCTGCTCATGCTTGCAA |
| CCGCTGGGATGCACGTGCATGCCTCTAGCATGAAGTCAGACAA |
| >GS_C1m (*Cladocopium*) |
| AGCCAATGGCCTCCTGAACGTGCGTTGCACTCTTGGGATTTCCTGAGAGTATGTCTGCTT |
| CAGTGCTTAACTTGCCCCAACTTTGCAAGCAGGATGTGTTTCTGCCTTGCGTTCTTACGA |
| GCTATTGCCCTCTGAGCCAATGGCTTGTTAATTGCTTGGTTCTTGCAAAATGCTTTGCGC |
| GCTGTTATTCAGGTTTCTACCTTCGTGGTTTTACTTGAGTGACGCTGCTCATGCTTGCAA |
| CCGCTGGGATGCAGGTGCATGCCTCTAGCATGAAGTCAGACAA |
| >GS_C1r (*Cladocopium*) |
| AACCAATGGCCTCCTGAACGTGCGTTACACTCTTGGGATTTCCTGAGAGTATGTCTGCTT |
| CAGTGCTTAACTTGCCCCAACTTTGCAAGCAGGATGTGTTTCTGCCTTGCGTTCTTATGA |
| GCTATTGCCCTCTGAGCCAATGGCTTGTTAATTGCTTGGTTCTTGCAAAATGCTTTGCGC |
| GCTGTTATTCAGGTTTCTACCTTCGTGGTTTTACTTGAGTGACGCTGCTCATGCTTGCAA |
| CCGCTGGGATGCAGGTGCATGCCTCTAGCATGAAGTCAGACAA |
| >GS_C1s (*Cladocopium*) |
| AACCAATGGCCTCCTGAACGTGCGTTGCACTCTTGGGATTTCCTGAGAGTATGTCTGCTT |
| CAGTGCTTAACTTGCCCCAACTTTGCAAGCAGGATGTGTTTCTGCCTTGCGTTCTTATGA |
| GCTATTGCCCTCTGAGCCAATGGCTTGTTAATTGCTTGGTTCTTGCAAAATGCTTTGCGC |
| GCTGTTATTCAGGTGACGCTGCTCATGCTTGCAACCGCTGGGATGCAGGTGCATGCCTCT |
| AGCATGAAGTCAGACAA |
| >GS_C1t (*Cladocopium*) |
| AACCAATGGCCTCCTGAACGTGCGTTGCACTCTTGGGATTTCCTGAGAGTATGTCTGCTT |
| CAGTGCTTAACTTGCCCCAACTTTGCAAGCAGGATGTGTTTCTGCCTTGCGTTCTTATGA |
| GCTATTGCCCTCTGAGCCAATGGCTTGTGAATTGCTTGGTTCTTGCAAAATGCTTTGCGC |
| GCGGTTATTCAGGTTTCTACCTTCGTGGTTTTACTTGAGTGACGCTGCTCATGCTTGCAA |
| CCGCTGGGATGCAGGTGCATGCCTCTAGCATGAAGTCAGACAA |
| >GS_C1p=C1.8 (*Cladocopium*) |
| AATCAATGGCCTCCTGAACGTGCGTTGCACTCTTGGGATTTCCTGAGAGTATGTCTGCTT |
| CAGTGCTTAACTTGCCCCAACTTTGCAAGCAGGATGTGTTTCTGCCTTGCGTTCTTATGA |
| GCTATTGCCCTCTGAGCCAATGGCTTGTTAATTGCTTGGTTCTTGCAAAATGCTTTGCGC |
| GCTGTTATTCAGGTTTCTACCTTCGTGGTTTTACTTGAGTGACGCTGCTCATGCTTGCAA |
| CCGCTGGGATGCAGGTGCATGCCTCTAGCATGAAGTCAGACAA |
| >GS_C1aa (*Cladocopium*) |
| AACCAATGGCCTCCTGAACGTGCGTTGCACTCTTGGGATTTCCTGAGAGTATGTCTGCTT |
| CAGTGCTTAACTTCCAACTTTGCAAGCAGGATGTGTTTCTGCCTTGCGTGCTTATGAGCT |
| ATTGCCCTCTGAGCCAATGGTTTGTGAATCGCTTGGTTCTTGCAAAATGCTTTGCGCGCT |
| GTTATTCAGGTATCTACCTTCGTGGTTTTACTTGAGTGACGCTGCTCATGCTTGCAACCG |
| CTGGGATGCAGGTGCATGCCTCTAGCATGAAGTCAGACAA |
| >GS_C# (*Cladocopium*) |
| AACCAATGGCCTCCTGAACGTGCGTTGCACTCTTGGGATTTCCTGAGAGTATGTCTGCTT |
| CAGTGCTTAACTTGCCCCAACTTTGCAAGCAGGATGTGTTTCTGCCTTGCGTTCTTATGA |
| GCTATTGCCCTCTGAGCCAATGGCTTGTTAATTGCTTGGTTCTTGCAAACTGCTTTGCGC |
| GCTGTTATTCAGGTTTCTACCTTCGTGGTTTTACTTGAGTGACGCTGCTCATGCTTGCAA |
| CCGCTGGGATGCAGGTGCATGCCTCTAGCATGAAGTCAGACAA |
| >GS_C1# (*Cladocopium*) |
| AACCAATGGCCTCCTGAACGTGCGTTGCACCCTTGGGATTTCCTGAGAGTATGTCTGCTT |
| CAGTGCTTAACTTGCTCCAACTTTGCAAGCAGGATGTGTTTCTGCCTTGCGTTCTTATGA |
| GCTATTGCCTTCTGCGCCAATGGCTTGTTAATTGCTTGGTTCTTGCAAAATGCTTTGCGC |
| GCTGTTATTCAAGTTTCTACCTTCGCGGTTTTACTTGAGTGACGCTGCTCATGCTTGCAA |
| CCGCTGGGATGCAGGTGCATGCCTCTAGCATGAAGTCAGACAA |
| >GS_C1.5 (*Cladocopium*) |
| AACCAATGGCCTCCTGAACGTGCGTTGCACTCTTGGGATTTCCTGAGAGTATGTCTGCTT |
| CAGTGCTTAACTTGCCCCAACTTTGCAAGCAGGATGTGTTTCTGCCTTGCGTTCTTATGA |
| GCTATTGCCCTCTGAGCCAATGGCTTGTTAATTGCTTGGTTCTTGCAAAATGCTTTGCGC |
| GCTGTTATTCAGGTTTCTACCTTCGTGGTCTTACTTGAGTGACGCTGCTCATGCTTGCAA |
| CCGCTGGGATGCAGGTGCATGCCTCTAGCATGAAGTCAGACAA |
| >GS_C1.6 (*Cladocopium*) |
| AACCAATGGCCTCCTGAACGCGCGTTGCACTCTTGGGATTTCCTGAGAGTATGTCTGCTT |
| CAGTGCTTAACTTGCCCCAACTTTGCAAGCAGGATGTGTTTCTGCCTTGCGTTCTTATGA |
| GCTATTGCCCTCTGAGCCAATGGCTTGTTAATTGCTTGGTTCTTGCAAAATGCTTTGCGC |
| GCTGTTATTCAGGTTTCTACCTTCGTGGTTTTACTTGAGTGACGCTGCTCATGCTTGCAA |
| CCGCTGGGATGCAGGTGCATGCCTCTAGCATGAAGTCAGACAA |
| >GS_C1.7 (*Cladocopium*) |
| AACCAATGGCCTCCTGAACGTGCGTTGCACTCTTGGGATTTCCTGAGAGTATGTCTGCTT |
| CAGTGCTTAACTTGCCCCAACTTTGCAGGCAGGATGTGTTTCTGCCTTGCGTTCTTATGA |
| GCTATTGCCCTCTGAGCCAATGGCTTGTTAATTGCTTGGTTCTTGCAAAATGCTTTGCGC |
| GCTGTTATTCAGGTTTCTACCTTCGTGGTTTTACTTGAGTGACGCTGCTCATGCTTGCAA |
| CCGCTGGGATGCAGGTGCATGCCTCTAGCATGAAGTCAGACAA |
| >GS_C1.v1a (*Cladocopium*) |
| AACCAATGGCCTCCTGAACGTGCGTTGCACTCTTGGGATTTCCTGAGAGTATGTCTGCTT |
| CAGTGCTTAACTTGCCCCAACTTTGCAAGCAGGATGTGTTTCTGCCTTGCGTTCTTATGA |
| GCTATTGCCCTCTGAGCCAATGGCTTGTTAATTGCTTGGTTCTTGCAAAATGCTTTGCGC |
| GCTGTTATTCAGGTTTCTACCTTCGTGGTTTTACTTGAGTGACGCTGCTCATGCTTGCAA |
| CCGCTGGGATGCAGGTGCATGCCTCTAGCATGAAGTCAGATAA |
| >GS_C1.v1b (*Cladocopium*) |
| AACCAATGGCCTCCTGAACGTGCGTTGCACTCTTGGGATTTCCTGAGAGTATGTCTGCTT |
| CAGTGCTTAACTTGCCCCAACTTTGCAAGCAGGATGTGTTTCTGCCTTGCGTTCTTATGA |
| GCTATTGCCCTCTGAGCCAATGGCTTGTTAATTGCTTGGTTCTTGCAAAATGCTTTGCGC |
| GCTGTTATTCAGGTTTCTACCTTCGTGGTTTTACTTGAGTGACGTTGCTCATGCTTGCAA |
| CCGCTGGGATGCAGGTGCATGCCTCTAGCATGAAGTCAGATAA |
| >GS_C2 (*Cladocopium*) |
| AACCAATGGCCTCCTGAACGTGCGTTGCACTCTTGGGATTTCCTGAGAGTATGTCTGCTT |
| CAGTGCTTAACTTGCCCCAACTTTGCAAGCAGGATGTGTTTCTGCCTTGCGTTCTTATGA |
| GCTATTGCCTTCTGCGCCAATGGCTTGTTAATTGCTTGGTTCTTGCAAAATGCTTTGCGC |
| GCTGTTATTCAAGTTTCTACCTTCGCGGTTTTACTTGAGTGACGCTGCTCATGCTTGCAA |
| CCGCTTGGGATGCAGGTGCATGCCTCTAGCATGAAGTCAGACAA |
| >LJ_C2r (*Cladocopium*) |
| TTGTCTGACTTCATGCTAGAGGCATGCACCTGCATCCCAGCGGTTGCAAGCATGAGCAGCGTCACTCAAGTAAAACCACGAAGGTAGAAACTTGAATAACAGCGCGCAAAGCATTTTGCAAGAACCAAGCAATTAACAAGCCATTGGCTCAGAGGGCAATAGCTCATAAGAATGCAAGGCAGAAACACATCCTGCTTGCAAAGTTGGGGCAAGTTAAGCACTGAAGCAGACATACTCTCAGGAAATCCCAAGAGTGCAACGCACGTTCAGGAGGCCATTGGTTCACGGAGTT |
| >GS_C3_new (*Cladocopium*) |
| AACCAATGGCCTCCTGAACGTGCGTTGCACTCTTGGGATTTCCTGAGAGTATGTCTGCTT |
| CAGTGCTTAACTTGCCCCAACTTTGCAATCAGGATGTGTTTCTGCCTTGCGTTCTTATGA |
| ACTATTGCCCTCTGAGCCAATGGCTTGKTAATTGCTTGGTTCTTGCAAAATGCTTTGCGC |
| GCTGTTATTCAAGTTTCTACCTTCSTGGTTTTACTTGAGTGACGCTGCTCATGC |
| >GS_C3aa (*Cladocopium*) |
| AAGCAATGGCCTCCTGAACGTGCGTTGCACTCTTGGGATTTCCTGAGAGTATGTCTGCTT |
| CAGTGCTTAACTTGCCCCAACTTTGCAAGCAGGATGTGTTTCTGCCTTGCGTTCTTATGA |
| GCTATTGCCCTCTGAGCCAATGGCTTGTTAATTGCTTGGTTCCTGCAAAATGCTTTGCGC |
| GCTGTTATTCAAGTTTCTACCTTCGCGGTTTTACTTGAGTGACGCTGCTCATGCTTGCAA |
| CCGCTGGGATGCAGGTGCATGCCTCTAGCATGAAGTCAGACAA |
| >GS_C3ff (*Cladocopium*) |
| AATCAATGGCCTCCTGAACGTGCGTTGCACTCTTGGGATTTCCTGAGAGTATGTCTGCTT |
| CAGTGCTTAACTTGCCCCAACTTTGCAAGCAGGATGTGTTTCTGCCTTGCGTGCTTATGA |
| GCTATTGCCCTCTGAGCCAATGGCTTGTTACTTGCTTGGTTCTTGCAAAATGCTTTGCGC |
| GCTGTTATTCAAGTTTCTACCTTCGTGGTTTTACTTGAGTGACGCTGCTCATGCTTGCAA |
| CCGCTGGGATGCAGGTGCATGCCTCTAGCATGAAGTCAGACAA |
| >GS_C3a (*Cladocopium*) |
| AACCAATGGCCTCCTGAACGTGCGTTGCACTCTTGGGATTTCCTGAGAGTATGTCTGCTT |
| CAGTGCTTAACTTGCCCCAACTTTGCAAGCAGGATGTGTTTCTGCCTTGCGTTCTTATGA |
| GCTATTGCCCTCTGAGCCAATGGCTTGTTAATTGCTTGGTTCTTGCAAAATGCTTTGCGC |
| GCTGTTATTCAAGTTTCTACCTTCGTGGTTTTACTTGAGTGATGCTGCTCATGCTTGCAA |
| CCGCTGGGATGCAGGTGCATGCCTCTAGCATGAAGTCAGACAA |
| >GS_C3b (*Cladocopium*) |
| AACCAATGGCCTCCTGAACGTGCGTTGCACTCTTGGGATTTCCTGAGAGTATGTCTGCTT |
| CAGTGCTTAACTTGCCCAACTTTGCAAGCAGGATGTGTTTCTGCCTTGCGTTCTTATGAG |
| CTATTGCCCTCTGAGCCAATGGCTTGTTAATTGCTTGGTTCTTGCAAAATGCTTTGCGCG |
| CTGTTATTCAAGTTTCTACCTTCGTGGTTTTACTTGAGTGACGCTGCTCATGCTTGCAAC |
| CGCTGGGATGCAGGTGCATGCCTCTAGCATGAAGTCAGACAA |
| >GS_C3d=C21 (*Cladocopium*) |
| AACCAATGGCCTCCTGAACGTGCGTTGCACTCTTGGGATTTCCTGAGAGTATGTCTGCTT |
| CAGTGCTTAACTTGCCCCAACTTTGCAAGCATTTCTGCCTTGCGTTCTTATGAGCTATTG |
| CCCTCTGAGCCAATGGCTTGTTAATTGCTTGGTTCTTGCAAAATGCTTTGCGCGCTGTTA |
| TTCAAGTTTCTACCTTCGTGGTTTTACTTGAGTGACGCTGCTCATGCTTGCAACCGCTGG |
| GATGCAGGTGCATGCCTCTAGCATGAAGTCAGACAA |
| >GS_C3e (*Cladocopium*) |
| AACCAATGGCCCCCTGAACGTGCGTTGCACTCTTGGGATTTCCTGAGAGTATGTCTGCTT |
| CAGTGCTTAACTTGCCCCAACTTTGCAAGCAGGATGTGTTTCTGCCTTGCGTTCTTATGA |
| GCTATTGCCCTCTGAGCCAATGGCTTGTTAATTGCTTGGTTCTTGCAAAATGCTTTGCGC |
| GCTGTTATTCAAGTTTCTACCTTCGTGGTTTTACTTGAGTGACGCTGCTCATGCTTGCAA |
| CCGCTGGGATGCAGGTGCATGCCTCTAGCATGAAGTCAGACAA |
| >GS_C3f (*Cladocopium*) |
| AACCAATGGCCTCCTGAACGTGCGTTGCACTCTTGGGATTTCCTGAGAGTATGTCTGCTT |
| CAGTGCTTAACTTGCCCCAACTTTGCAAGCAGGATGTGTTTCTGCCTTGCGTTCTTATGA |
| GCTATTGCCCTCTGAGCCAATGGCTTGTTAATTGCTTGGTTCTTGCAAAATGCTTTGCGC |
| GCTGTTATTCAAGTTTCTACCTTCGTTGTTTTACTTGAGTGACGCTGCTCATGCTTGCAA |
| CCGCTGGGATGCAGGTGCATGCCTCTAGCATGAAGTCAGACAA |
| >GS_C3g (*Cladocopium*) |
| AACCAATGGCCTCCTGAACGTGTGTTGCACTCTTGGGATTTCCTGAGAGTATGTCTGCTT |
| CAGTGCTTAACTTGCCCCAACTTTGCAAGCAGGATGTGTTTCTGCCTTGCGTTCTTATGA |
| GCTATTGCCCTCTGAGCCAATGGCTTGTTAATTGCTTGGTTCTTGCAAAATGCTTTGCGC |
| GCTGTTATTCAAGTTTCTACCTTCGTGGTTTTACTTGAGTGACGCTGCTCATGCTTGCAA |
| CCGCTGGGATGCAGGTGTGCATGCCTCTAGCATGAAGTCAGACAA |
| >GS_C3h (*Cladocopium*) |
| AACCAATGGCCTCCTGAACGTGCGTTGCACTCTTGGGGTTTCCTGAGAGTATGTCTGCTT |
| CAGTGCTTAACTTGCCCCAACTTTGCAAGCATTTCTGCCTTGCGTTCTTATGAGCTATTG |
| CCCTCTGAGCCAATGGCTTGTTAATTGCTTGGTTCTTGCAAAATGCTTTGCGCGCTGTTA |
| TTCAAGTTTCTACCTTCGTGGTTTTACTTGAGTGACGCTGCTCATGCTTGCAACCGCTGG |
| GATGCAGGTGCATGCCTCTAGCATGAAGTCAGACAA |
| >GS_C3ha (*Cladocopium*) |
| AACCAATGGCCTCCTGAACGTGCGTTGCACTCTTGGGATTTCCTGAGAGTATGTCTGCTT |
| CAGTGCTTAACTTGCCCCAACTTTGCAAGCATTTCTGCCTTGCGTTCTTATGAGCTATTG |
| CCCTCTGAGCCAATGGTTTGTTAATTGCTTGGTTCTTGCAAAATGCTTTGCGCGCTGTTA |
| TTCAAGGTTCTACCTTCGTGGTTTTACTTGAGTGACGCTGCTCATGCTTGCAACCGCTGG |
| GATGCAGGTGCATGCCTCTAGCATGAAGTCAGACAA |
| >GS_C3j (*Cladocopium*) |
| AAGCAATGGCCTCCTGAACGTGCGTTGCACTCTTGGGATTTCCTGAGAGTATGTCTGCTT |
| CAGTGTTTAACTTGCCCCAACTTTGCAAGCAGGATGTGTTTCTGCCTTGCGTTCTTATGA |
| GCTATTGCCCTCTGAGCCAATGGCTTGTTAATTGCTTGGTTCTTGCAAAATGCTTTGCGC |
| GCTGTTATTCAAGTTTCTACCTTCGTGGTTTTACTTGAGTGACGCTGCTCATGCTTGCAA |
| CCGCTGGGATGCAGGTGCATGCCTCTAGCATGAAGTCAGACAA |
| >GS_C3k (*Cladocopium*) |
| AACCAATGGCCTCCTGAATGTGCGTTGCACTCTTGGGATTTCCTGAGAGTATGTCTGCTT |
| CAGTGCTTAACTTGCCCCAACTTTGCAAGCAGGATGTGTTTCTGCCTTGCGTTCGTATGA |
| GCTGTTGCCCTCTGAGCCAATGGCTTGTTAATTGCTTGGTTCTTGCAAAATGCTTTGCGC |
| GCTGTTATTCAAGTTTCTACCTTCGTGGTTTTACTTGAGTGATGCTGCTCATGCTTGCAA |
| CCGCTGGGATGCAGGTGCATGCCTCTAGCATGAAGTCAGACAA |
| >GS_C3L (*Cladocopium*) |
| AACCAATGGCCTCCTGAACGTGCGTTGCACTCTTGGGATTTCCTGAGAGTATGTCTGCTT |
| CAGTGCTTAACTTGCCCAACTTTGCAAGCAGGATGTGTTTCTGCCTGCGTTCTTATGAGC |
| TATTGCCCTCTGAGCCAATGGCTTGTTAATTGCTTGGTTCTTGCAAAATGCTTTGCGCGC |
| TGTTATTCAAGTTTCTACCTTCGTGGTTTTTTTACTTGAGTGACGCTGCTCATGCTTGCA |
| ACCGCTGGGATGCAGGTGCATGCCTCTAGCATGAAGTCAGACAA |
| >GS_C3n (*Cladocopium*) |
| AATCAATGGCCTCCTGAACGTGCGTTGCACTCTTGGGATTTCCTGAGAGTATGTCTGCTT |
| CAGTGCTTAACTTGCCCCAACTTTGCAAGCAGGATGTGTTTCTGCCTTGCGTTCTTATGA |
| GCTATTGCCCTCTGAGCCAATGGCTTGTTACTTGCTTGGTTCTTGCAAAATGCTTTGCGC |
| GCTGTTATTCAAGTTTCTACCTTCGTGGTTTTACTTGAGTGACGCTGCTCATGCTTGCAA |
| CCGCTGGGATGCAGGTGCATGCCTCTAGCATGAAGTCAGACAA |
| >GS_C3t (*Cladocopium*) |
| AACCAATGGCCTCCTGAACGTGCGTTGCACTCTTGGGATTTCCTGAGAGTATGTCTGCTT |
| CAGTGCTTAACTTGCCCCAACTTTGCAAGCAGGATGTGTTTCTGCCTTGCGTTCTTATGA |
| GCTATTGCCCTCTGAGCCAATGGCTTGTTACTTGCTTGGTTCTTGCAAAATGCTTTGCGC |
| GCTGTTATTCAAGTTTCTACCTTCGTGGTTTTACTTGAGTGACGCTGCTCATGCTTGCAA |
| ACGCTGGGATGCAGGTGCATGCCTCTAGCATGAAGTCAGACAA |
| >GS_C3p (*Cladocopium*) |
| AACCAATGGCCTCCTGAACGTGCGTTGCACTCTTGGGATTCCTGAGAGTATGTCTGCTTC |
| AGTGCTTGACTTGCCCCAACTTTGCAAGCAGGATGTGTTTCTGCCTTGCGTTCTTATGAG |
| CTATTGCCCTCTGAGCCAATGGCTTGTTAATTGCTTGGTTCTTGCAAAATGCTTTGCGCG |
| CTGTTATTCAAGTTTCTACCTTCGTTGTTTTACTTGAGTGACGCTGCTCATGCTTGCAAC |
| CGCTGGGATGCAGGTGCATGCCTCTAGCATGAAGTCAGACAA |
| >GS_C3q (*Cladocopium*) |
| AACCAATGGCCTCCTGAACGTGCGTTGCACTCTTGGGATTTCCTGAGAGTATGTCTGCTT |
| CAGTGCTTTAACTTGCCCCAACTTTGCAAGCATTTCTGCCTTGCGTTCATATGAGCTATT |
| GCCCTCTGAGCCAATGGCTTGTTAATTACTTGGTTCTTGCGAAATGGTTTGTGCGCTGTT |
| ATTCAAGTTTCTACCTTTGTGGTTTTACTTGAGTGACGCTGCTCATGCTTGCAACCGCTG |
| GGATGCAGGTGCATGCCTCTAGCATGAAGTCAGACAA |
| >LJ_C3s (*Cladocopium*) |
| TTGTCTGACTTCATGCTAGAGGCATGCACCTGCATCCCAGCGGTTGCAAGCATGAGCAGCGTCACTCAAGTAAAACCACGAAGGTAGAAACTTGAATAACAGCGCGCAAAGCATTTTGCAAGAACCAAGCAATTAACAAGCCATTGGCTCAGAGGGCAATAGCTCATAAGAACGCAAGGCAGAAACACATCCTGCTTGCAAAGTTGGGGCAAGTTAAGCACTGAAGCAGACATACTCTCAGGAAATCCCAAAAGTGCAACGCACGTTCAGGAGGCCATTGGTTCA |
| >GS_C3u (*Cladocopium*) |
| AAGCAATGGCCTCCTGAACGTGCGTTGCACTCTTGGGATTTCCTGAGAGTATGTCTGCTT |
| CAGTGCTTAACTTGCCCCAACTTTGCAAGCAGGATGTGTTTCTGCCTTGCGTTCTTATGA |
| GCTATTGCCCTCTGAGCCAATGGCTTGTTAATTGCTTGGTTCTTGCAAAATGCTTTGCGC |
| GCTGTTATTCAAGTTTCTACCTTCGCGGTTTTACTTGAGTGACGCTGCTCATGCTTGCAA |
| CCGCTGGGATGCAGGTGCATGCCTCTAGCATGAAGTCAGACAA |
| >GS_C3v (*Cladocopium*) |
| AACCAATGGCCTCCTGAACGTGCGTTGCACTCTTGGGATTTCCTGAGAGTATGTCTGCTT |
| CAGTGCTTAACTTGCCCCAACTTTGCAAGCAGGATGTGTTTCTGCCTTGCGTTCTTATGA |
| GCTATTGCCCTCTGAGCCAATGGCTTGTTCATTGCTTGGTTCTTGCAAAATGCTTTGCGC |
| GCTGTTATTCAGGTTTCTACCTTCGTGGTTTTACTTGAGTGATGCTGCTCATGCTTGCAA |
| CCGCTGGGATGCAGGGTGCATGCCTCTAGCATGAAGTCAGACAA |
| >GS_C3w (*Cladocopium*) |
| AATCAATGGCCTCCTGAACGTGCGTTGCACTCTTGGGATTTCCTGAGAGTATGTCTGCTT |
| CAGTGCTTAACTTGCCCCAACTTTGCAAGCAGGATGTGTTTCTGCCTTGCGTTCTTATGA |
| GCTATTGCCCTCTGAGCCAATGGCTTGTTAATTGCTTGGTTCTTGCAAAATGCTTTGCGC |
| GCTGTTATTCAAGTTTCTACCTTCGTGGTTTTACTTGAGTGACGCTGCTCATGCTTGCAA |
| CCGCTGGGATGCAGGTGCATGCCTCTAGCATGAAGTCAGACAA |
| >GS_C3y (*Cladocopium*) |
| AACCAATGGCCTCCTGAACGTGCGTTGCACTCTTGGGATTTCCTGAGAGTATGTCTGCTT |
| CAGTGCTTAACTTGCCCCAACTTTGCAAGCAGGATGTGTTTCTGCCTTGCGTTCTTATGA |
| GCTATTGCCCTCTGAGCCAGTGGCTTGTTAATTGCTTGGTTCTTGCAAAATGCTTTGCGC |
| GCTGTTATTCAAGTTTCTACCTTCGTGGTTTTACTTGAGTGACGCTGCTCATGCTTGCAA |
| CCGCTGGGATGCAGGTGCATGCCTCTAGCATGAAGTCAGACAA |
| >GS_C3z (*Cladocopium*) |
| AACCAATGGCCTCCTGAACGTGCGTTGCACTCTTGGGATTTCCTGAGAGTATGTCTGCTT |
| CAGTGCTTAACTTGCCCCAACTTTGCAAGCAGGATGTGTTTCTGCCTTGCGTTCTTATGA |
| GCTATTGCCCTCTGAGCCAATGGCTTGTTAATTGCTTGGTTCTTGCAAAATGCTTTGTGC |
| GCTGTTATTCAAGTTTCTACCTTCGTGGTTTTACTTGAGTGACGCTGCTCATGCTTGCAA |
| CTGCTGGGATGCAGGTGCATGCCTCTAGCATGAAGTCAGACAA |
| >GS_C3.2 (*Cladocopium*) |
| AACCAATGGCCTCCTGAACGTGCGTTGCACTCTTGGGATTTCCTGAGAGTATGTCTGCTT |
| CAGTGCTTAACTTGCCCCAACTTTGCAAGCAGATGTGTTTCTGCCTTGCGTTCTTATGAG |
| CTATTGCCCTCTGAGCCAATGGCTTGTTAATTGCTTGGTTCTTGCAAAATGCTTTGCGCG |
| CTGTTATTCAAGTTTCTACCTTCGTGGTTTTACTTGAGTGACGCTGCTCATGCTTGCAAC |
| CGCTGGGATGCAGGTGCATGCCTCTAGCATGAAGTCAGACAA |
| >GS_C3.7 (*Cladocopium*) |
| AACCAATGGCCTCCTGAACGTGCGTTGCACTCTTGGAATTTCCTGAGAGTATGTCTGCTT |
| CAGTGCTTAACTTGCCCCAACTTTGCAAGCAGGATGTGTTTCTGCCTTGCGTTCTTATGA |
| GCTATTGCCCTCTGAGCCAATGGCTTGTTAATTGCTTGGTTCTTGCAAAATGCTTTGCGC |
| GCTGTTATTCAAGTTTCTACCTTCGTGGTTTTACTTGAGTGACGCTGCTCATGCTTGCAA |
| CCGCTGGGATGCAGGTGCATGCCTCTAGCATGAAGTCAGACAA |
| >GS_C3.8 (*Cladocopium*) |
| AACCAATGGCCTCCTGAACGTGCGTTGCACTCTTGGGATTTCCTGAGAGTATGTCTGCTT |
| CAGTGCTTAACTTGCCCCAACTTTGCAAGCAGGATGTGTTTCTGCCTTGCGTTCTTATGA |
| GCTATTGCCCTCTGAGCCAATGGCTTGTTAATTGCTTGGTTCTTGCAAAATGCTTTGCGC |
| GCTGTTATTCAAGTTTCTATCTTCGTGGTTTTACTTGAGTGACGCTGCTCATGCTTGCAA |
| CCGCTGGGATGCAGGTGCATGCCTCTAGCATGAAGTCAGACAA |
| >GS_C3.9 (*Cladocopium*) |
| AACCAATGGCCTCCTGAACGTGCGTTGCACTCTTGGGATTTCCTGAGAGTATGTCTGCTT |
| CAGTGCTTAACTTGCCCAACTTTGCAAGCAGGATGTGTTTCTGCCTTGCGTTCTTATGAG |
| CTATTGCCCTCTGAGCCAATGGCTTGTTAATTGCTTGGTTCTTGCAAAATGCTTTGCGCG |
| CTGTTATTCAAGTTTCTATCTTCGTGGTTTTACTTGAGTGACGCTGCTCATGCTTGCAAC |
| CGCTGGGAGCAGGTGCATGCCTCTAGCATGAAGTCAGACAA |
| >GS_C3.10 (*Cladocopium*) |
| AACCAATGGCCTCCTGAACGTGCGTTGCACTCTTGGGATTTCCTGAGAGTATGTCTGCTT |
| CAGTGCTTAACTTGCCCCAACTTTGCAAGCAGGATGTGTTTCTGCCTTGCGTTCTTATGA |
| GCTATTGCCCTCTGAGCCAATGGCTTGTTAATTGCTTGGTTCTTGCAAAATGCTTTGCGC |
| GCTGTTATTCAAGTTTCTACCTTCGTGGTTTTACTTGAGTGACGCTGCTCATGCTTGCAA |
| CTGCTGGGATGCAGGTGCATGCCTCTAGCATGAAGTCAGACAA |
| >GS_C3.11 (*Cladocopium*) |
| AACCAATGGCCTCCTGAACGTGCGTTGCACTCTTGGGATTTCCTGAGAGTATGTCTGCTT |
| CAGTGCTTAACGTGCCCCAACTTTGCAAGCAGGATGTGTTTCTGCCTTGCGTTCTTATGA |
| GCTATTGCCCTCTGAGCCAATGGCTTGTTAATTGCTTGGTTCTTGCAAAATGCTTTGCGC |
| GCTGTTATTCAAGTTTCTACCTTCGTGGTTTTACTTGAGTGACGCTGCTCATGCTTGCAA |
| CCGCTGGGATGCAGGTGCATGCCTCTAGCATGAAGTCAGACAA |
| >GS_C3.12 (*Cladocopium*) |
| AACCAATGGCCTCCTGAACGTGCGTTGCACTCTTGGGATTTCCTGAGAGTATGTCTGCTT |
| CAGTGCTTAACTTGCCCCAACTTTGCAAGCAGGATGTGTTTCTGCCTTGCGTTCTTATGA |
| GCTATTGCCCTCTGAGCCAATGGCTTGTGAATTGCTTGGTTCTTGCAAAATGCTTTGCGC |
| GCTGTTATTCAAGTTTCTACCTTCGTGGTTTTACTTGAGTGACGCTGCTCATGCTTGCAA |
| CCGCTGGGATGCAGGTGCATGCCTCTAGCATGAAGTCAGACAA |
| >GS_C3.14 (*Cladocopium*) |
| AACCAATGGCCTCCTGAACGTACGTTGCACTCTTGGGATTTCCTGAGAGTATGTCTGCTT |
| CAGTGCTTAACTTGCCCCAACTTTGCAAGCAGGATGTGTTTCTGCCTTGCGCTCTTATGA |
| GCTATTGCCCTCTGAGCCAATGGCTTGTTAATTGCTTGGTTCTTGCAAAATGCTTTGCGC |
| GCTGTTATTCAAGTTTCTACCTTCGTGGTTTTACTTGAGTGACGCTGCTCATGCTTGCAA |
| CCGCTGGGATGCAGGTGCATGCCTCTAGCATGAAGTCAGACAA |
| >GS_C4 (*Cladocopium*) |
| AACCAATGGCCTCCTGAACGTGCGTTGCACTCTTGGGATTTCCTGAGAGTATGTTTGCTT |
| CAGTGCTTAACTTGCCCCAACTTTGCAAGCAGGATGTGTTTCTGCCTTGCGTTCTTATGA |
| GCTATTGCCCTCTGAGCCAATGGCTTGTGAATTGCTTGGTTCTTGCAAAATGCTTTGCGC |
| GCTGTTATTCAGGTTTCTACCTTCGTGGTTTTACTTGAGTGACGCTGCTCATGCTTGCAA |
| CCGCTGGGATGCAGGTGCATGCCTCTAGCATGAAGTCAGACAA |
| >GS_C5 (*Cladocopium*) |
| AACCAATGGCCTCCTGAACGTGCGTTGCACTCTTGGGATTTCCTGAGAGTATGTCTTCTT |
| CAGTGCTTAACTTGCCCCAACTTTGCAAGCAGGATATGTTTCTGCCTTGCGTTCTTATGA |
| GCTATTGCCCTCTGCGCCAATGGCTTGTTAATTGCTTGGTTCTTGCAAAATGCTTTGCGC |
| GCTGTTATTCAAGTTTCTACCCTCGCGGTTTTACTTGAGTGACGCTGCTCATGCTTGCAA |
| CCGCTGGGATGCAGGTGCATGCCTCTAGCATGAAGTCAGACAA |
| >GS_C6 (*Cladocopium*) |
| AACCAATGGCCTCCTGAACGTGCGTTGCACTCTTGGGATTTCCTGAGAGTATGTCTGCTT |
| CAGTGCTTACCTTGCCCCAACTTTGCAAGCAGGATGTGTTTCTGCCTTGCGTGCTTATGA |
| GCTATTGCCCTCTGAGCCAATGGCTTGTTAATTGCTTGGTTCTTGCAAAATGCTTTGCGC |
| GCTGTTATTCAAGTTTCTACCTTCGTGGTTTTACTTGAGTGACGCTGCTCATGCTTGCAA |
| CCGCTGGGATGCAGGTGCATGCCTCTAGCATGAAGTCAGACAA |
| >GS_C7 (*Cladocopium*) |
| AACCAATGGCCTCCTGAACGTGCGTTGCACTCTTGGGATTTCCTGAGAGTATGTCTGCTT |
| CAGTGCTTAACTTGCCCCAACTTTGCAAGCAGGATGTGTTTCTGCCTTGTGTTCTTATGA |
| GCTATTGCCCTCTGAGCCAATGGCTTGTTAATTGCTTGGTTCTTGCAAAATGCTTTGCGC |
| GCTGTTATTCAAGTTTCTACCTTCGTGGTTTTACTTGAGTGACGCTGCTCATGCTTGCAA |
| CCGCTGGGATGCAGGTGCATGCCTCTAGCATGAAGTCAGACAA |
| >GS_C8 (*Cladocopium*) |
| AACCAATGGCCTCCTGAACGTGCGTTGCACTCTTGGGATTTCCTGAGAGTATGTCTGCTT |
| CAGTCCTTAACTTGCCCCAACTTTGCAAGCAGGATGTGTTTCTGCCTTGCGTTCTTATGA |
| GCTATTGCCCTCTGAGCCAATGGCTTGTGAATTGCTTGGTTCTTGCAAAATGCTTTGCGC |
| GCTGTTATTCAGGTTTCTACCTTCGTGGTTTCACTTGAGTGACGCTGCTCATGCTTGCAA |
| CCGCTGGGATGCAGGTGCATGCCTCTAGCCTGAAGTCAGACAA |
| >GS_C8a (*Cladocopium*) |
| AACCAATGGCCTCCTGAACGTGCGTTGCACTCTTGGGATTTCCTGAGAGTATGTCTGCTT |
| CAGTCCTTAACTAGCCCCAACTTTGCAAGCAGGATGTGTTTCTGCCTTGCGTTCTTATGA |
| GCTATTGCCCTCTGAGCCAATGGCTTGTGAATTGCTTGGTTCTTGCAAAATGCTTTGCGC |
| GCTGTTATTCAGGTTTCTACCTTCGTGGTTTCACTTGAGTGACGCTGCTCATGCTTGCAA |
| CCGCTGGGATGCAGGTGCATGCCTCTAGCCTGAAGTCAGACAA |
| >GS_C8b (*Cladocopium*) |
| AACCAATGGCCTCCTGAACGTGCGTTGCACTCTTGGGATTTCCTGAGAGTGTGTCTGCTT |
| CAGTCCTTAACTTGCCCCAACTTTGCAAGCAGGATGTGTTTCTGCCTTGCGTTCTTATGA |
| GCTATTGCCCTCTGAGCCAATGGCTTGTGAATTGCTTGGTTCTTGCAAAATGCTTTGCGC |
| GCTGTTATCAGGTTTCTACCTTCGTGGTTTCACTTGAGTGACGCTGCTCATGCTTGCAAC |
| CGCTGGGATGCAGGTGCATGCCTCTAGCCTGAAGTCAGACAA |
| >GS_C8c (*Cladocopium*) |
| AACCAATGGCCTCCTGAACGTGCGTTGCACTCTTGGGATTTCCTGAGAGTATGTCTGCTT |
| CAGTCCTTAACTTGCCCCAACTTTGCAAGCAGGATGTGTTTCTGCCCTGCGTTCTTATGA |
| GCTATTGCCCTCTGAGCCAATGGCTTGTGAATTGCTTGGTTCTTGCAAAATGCTTTGCGC |
| GCTGTTATTCAGGTTTCTACCTTCGTGGTTTCACTTGAGTGACGCTGCTCATGCTTGCAA |
| CCGCTGGGATGCAGGTGCATGCCTCTAGCCTGAAGTCAGACAA |
| >GS_C9 (*Cladocopium*) |
| AACCAATGGCCTCCTGAACGTGCGTTGCACTCTTGGGATTTCCTGAGAGTATGTCTGCTT |
| CAGTGCTTAACTTGCCCCAACTTTGCAAGCAGGATGTGTTTCTGCCTTGCATTCCTATGA |
| GCTATTGCCCTCTGAGCCAATGGCTTGTGAATTGCTTGGTTCTTGCAAAATGCTTTGCGC |
| GCTGTTATTCAGGTTTCTACCTTCGTGGTTTTACTTGAGTGACGCTGCTCATGCTTGCAA |
| CCGCTGGGATGCAGGTGCATGCCTCTAGCATGAAGTCAGACAA |
| >GS_C10a (*Cladocopium*) |
| AACCAATGGCCTCCTGAACGTGCGTTGCACTCTTGGGATTTCCTGAGAGTATGTCTGCTT |
| CAGTGCTTAACTTGCCCCAACTTTGCAAGCAGGATGTGTTTCTGCCTTGCGTTCTTATGA |
| GCTATTGCCCTCTGCGCCAATGGCTTGTGAATTGCTTGGTTCTTGCAAAATGCTTTGCCC |
| GCTGTTATTCAGGTTTCTACCTTCGTGGTTTTACTTGAGTGACGCTGCTCATGCTTGCAA |
| CCGCTGGGATGCAGGTGCATGCCTCTAGCATGAAGTCAGACAA |
| >GS_C11 (*Cladocopium*) |
| AACCAATGGCCTCCTGAACGTGCGTTGCACTCTTGGGATTTCCTGAGAGTATGTCTGCTT |
| CAGTGCTTAACTTGCCCCAACTTTGCAAGCAGGATGTGTTTCTGCCTTGCGTTCTTATGA |
| GCTATTGCCCTCTGAGCCAATGGCTTGTTAATTGCTTGGTTCTTGCAAAATGCTTTGCGC |
| GATGTTATTCAAGTTTCTACCTTCGTGGTTTTACTTGAGTGACGCTGCTCATGCTTGCAA |
| CCGCTGGGATGCAGGTGCATGCCTCTAGCATGAAGTCAGACAA |
| >GS_C12 (*Cladocopium*) |
| AACCAATGGCCTCCTGAACGTGCGTTGCACTCTTGGGATTTCCTGAGAGTATGTCTCTGC |
| TTCAGTGCTTAACTTGCCCCAACTTTGCAAGCAGGATGTGTTTCTGCCTTGTGTTCTTAT |
| GAGCTATTGCCCTCTGAGCCAATGGCTTGTTAATTGCTTGGTTCTTGCAAAATGCTTTGC |
| GCGCTGTTATTCAAGTTTCTACCTTCGTGGTTTTACTTGAGTGACGCTGCTCATGCTTGC |
| AACCGCTGGGATGCAGGTGCATGCCTCTAGCATGAAGTCAGACAA |
| >GS_C10=C13 (*Cladocopium*) |
| AACCAATGGCCTCCTGAACGTGCGTTGCACTCTTGGGATTTCCTGAGAGTATGTCTGCTT |
| CAGTGCTTAACTTGCCCCAACTTTGCAAATGCAAGCAGGATGTGTTTCTGCCTTGCGTTC |
| TTATGAGCTATTGCCCTCTGAGCCAATGGCTTGTGAATTGCTTGGTTCTTGCAAAATGCT |
| TTGCCCGCTGTTATTCAGGTTTCTACCTTCGTGGTTTTACTTGAGTGACGCTGCTCATGC |
| TTGCAACCGCTGGGATGCAGGTGCATGCCTCTAGCATGAAGTCAGACAA |
| >GS_C14 (*Cladocopium*) |
| AATCAATGGCCTCCTGAACGTGCGTTGCACTCTTGGGATTTCCTGAGAGTATGTCTGCTT |
| CAGTGCTTAACTTGCCCCAACTTTGCAAGCAGGATGTGTTCTGCCTTGCGTTCTTATGAG |
| TTATTGTCCTCTGAGCCAATGGCTTGTGAATTGCTTGGTTCTTGCAAAATGCTTTGCGCG |
| CTGTTATTCAGGTTTCTACCTTCGTGGTTTTACTTGAGTGACGCTGCTCATGCTTGCAAC |
| CGCTGGGATGCAGGTGCATGCCTCTAGCATGAAGTCAGACAA |
| >GS_C15a (*Cladocopium*) |
| AACCAATGGCCTCCTGAACGTGCGTTGCACCCTTGGGATTTCCTGAGAGTATGTCTGCTT |
| CAGTGCTTAACTTGCCCCAACTTTGCAAGCAGGATGTGTTTCTGCCTTGCGTTCTTATGA |
| GCTATTGCCTTCTGCGCCAATGGCTTGTTAATTGCTTGGTTCTTGCAAAATGCTTTGCGC |
| GCTGTTATTCAAGTTTCTACCTTCGTGGTTTTACTTGAGTGACGCTGCTCATGCTTGCAA |
| CCGCTGGGATGCAGGTGCATGCCTCTAGCATGAAGTCAGACAA |
| >GS_C15b (*Cladocopium*) |
| AACCAATGGCCTCCTGAACGTGCGTTGCACCCTTGGGATTTCCTGAGAGTCTGTCTGCTT |
| CAGTGCTTAACTTGCCCCAACTTTGCAAGCAGGATGTGTTTCTGCCTTGCGTTCTTATGA |
| GCTATTGCCTTCTGCGCCAATGGCTTGTTAATTGCTTGGTTCTTGCAAAATGCTTTGCGC |
| GCTGTTATTCAAGTTTCTACCTTCGCGGTTTTACTTGAGTGACGCTGCTCATGCTTGCAA |
| CCGCTGGGATGCAGGTGCATGCCTCTAGCATGAAGTCAGACAA |
| >GS_C15c (*Cladocopium*) |
| AACCAATGGCCTCCTGAACGTGCCTTGCACCCTTGGGATTTCCTGAGAGTATGTCTGCTT |
| CAGTGCTTAACTTGCCCCAACTTTGCAAGCAGGATGTGTTTCTGCCTTGCGTTCTTATGA |
| GCTATTGACTTCTGCGCCAATGGCTTGTTAATTGCTTGGTTCTTGCAAAATGCTTTGCGC |
| GCTGTTATTCAAGTTTCTACCTTCGCGGTTTTACTTGAGTGACGCTGCTCATGCTTGCAA |
| CCGCTGGGATGCAGGTGCATGCCTCTAGCATGAAGTCAGACAA |
| >GS_C15d (*Cladocopium*) |
| AACCAATGGCCTCCTGAACGTGCCTTGCACCCTTGGGATTTCCTGAGAGTATGTCTGCTT |
| CAGTGCTTAACTTGCCCCAACTTTGCAAGCAGGATGTGTTTCTGCCTTGCGTTCTTATGA |
| GCTATTGACTTCTGCGCCAATGGCTTGTTAATTGCTTGGTTCTTGCAAAATGCTTTGCGC |
| ACTGTTATTCAAGTTTCTACCTTCGCGGTTTTACTTGAGTGACGCTGCTCATGCTTGCAA |
| CCGCTGGGATGCAGGTGCATGCCTCTAGCATGAAGTCAGACAA |
| >GS_C15e (*Cladocopium*) |
| AACCAATGGCCTCCTGAACGTGCGTTGCACCCTTGGGATTTCCTGAGAGTATGTCTGCTT |
| CAGTGCTTAACTTGCCCCAACTTTGCAAGCAGGATGTGTTTCTGCCTTGCGTTCTTATGA |
| GCTATTGCCTTCTGCGCCAATGGCTTGTTAATTGCTTGGTTCTTGCAAAATGCTTTGCGC |
| GCTGTTATTCAAGTTTCTACCTTCGCGGTTTTACTTGAGTGACGTGCTCATGCTTGCAAC |
| CGCAGGGATGCAGGTGCATGCCTCTAGCATGAAGTCAGACAA |
| >GS_C15f (*Cladocopium*) |
| AACCAATGGCCTCCTGAACGTGCGTTGCACCCTTGGGATTTCCTGAGAGTATGTCTGCTT |
| CAGTGCTTAACTTGCCCCAACTTTGCAAGCAGGATGTGTTTCTGCCTTGCGTTCTTATGA |
| GCTATTGCCTTCTGCGCCCAATGGCTTGTTAATTGCTTGGTTCTTGCAAAATGCTTTGCG |
| CGCTGTTATTCAAGTTTCTACCTTCGCGGTTTTACTTGAGTGACGCTGCTCATGCTTGCA |
| ACCGCTGGGATGCAGGTGCATGCCTCTAGCATGAAGTCAGACAA |
| >GS_C15g (*Cladocopium*) |
| AACCAATGGCCTCCTGAACGTGCGTTGCACCCTTGGGATTTCCTGAGAGTATGTCTGCTT |
| CAGTGCTTAACTTGCCCTAACTTTGCAAGCAGGATGTGTTTCTGCCTTGCGTTCTTATGA |
| GCTATTGCCTTCTGCGCCAATGGCTTGTTAATTGCTTGGTTCTTGCAAAATGCTTTGCGC |
| GCTGTTATTCAAGTTTCTACCTTCGCGGTTTTACTTGAGTGACGCTGCTCATGCTTGCAA |
| CCGCTGGGATGCAGGTGCATGCCTCTAGCATGAAGTCAGACAA |
| >GS_C15h (*Cladocopium*) |
| AATCAATGGCCTCCTGAACGTGCGTTGCACCCTTGGGATTTCCTGAGAGTATGTCTGCTT |
| CAGTGCTTAACTTGCCCCAACTTTGCAAGCAGGATGTGTTTCTGCCTTGCGTTCTTATGA |
| GCTATTGCCTTCTGCGCCAATGGCTTGTTAATTGCTTGGTTCTTGCAAAATGCTTTGCGC |
| GCTGTTATTCAAGTTTCTACCTTCGCGGTTTTACTTGAGTGACGCTGCTCATGCTTGCAA |
| CCGCTGGGATGCAGGTGCATGCCTCTAGCATGAAGTCAGACAA |
| >GS_C15i (*Cladocopium*) |
| AACCAATGGCCTCCTGAACGTGCGTTGCACCCTTGGGATTTCCTGAGAGTATGTCTGCTT |
| CAGTGCTTAACTTGCCCCAACTTTGCAAGCAGGATGTGTTTCTGCCTTGCGTTCTTATGA |
| GCTATTGCCTTCTGCGCCAATGGCTTGTTAATTGCTCGGTTCTTGCAAAATGCTTTGCGC |
| GCTGTTATTCAAGTTTCTACCTTCGCGGTTTTACTTGAGTGACGCTGCTCATGCTTGCAA |
| CCGCTGGGATGCAGGTGCATGCCTCTAGCATGAAGTCAGACAA |
| >GS_C15j (*Cladocopium*) |
| AACCAATGGCCTCCTGAACGTGCGTTGCACCCTTGGGATTTCCTGAGAGTATGTCTGCTT |
| CAGTGCTTAACTTGCCCCAACTTTGCAAGCAGGATGTGTTTCTGCCTTGCGTTCTTATGA |
| GCTATTGCCTTCTGCGCCAATGGCTTGTTAATTGCTTGGTTCTTGCAAAATGCTTTGCGC |
| GCTGTTATTCAAGTTTCTACCTTCGCGGTTTTACTTGAGTGACGCTGCTCGTGCTTGCAA |
| CCGCTGGGATGCAGGTGCATGCCTCTAGCATGAAGTCAGACAA |
| >GS_C15k (*Cladocopium*) |
| AACCAATGGCCTCCTGAACGTGCGTTGCACCCTTGGGATTTCCTGAGAGTATGTCTGCTT |
| CAGTGCTTAACTTGCCCCAACTTTGCAAGCAGGATGTGTTTCTGCCTTGCGTTCTTATGA |
| GCTATTGCCTTCTGCGCCAATGGCTTGTTAATTGCTTGGTTCTTGCAAAATGCTTTGCGC |
| GCTGTTATTCAAGTTTCTACCTTCGCGGTTTTACTTGAGTGACGCTGCTCATGCTTGCAA |
| CCGCTGGGATGCAGGTGCATGCCTCTAGACAGAAGTCAGACAA |
| >GS_C15L (*Cladocopium*) |
| AACCAATGGCCTCCTGAACGTGCGTTGCACCCTTGGGATTTCCTGAGAGTATGTCTGCTT |
| CAGTGCTTAACTTGCCCCAACTTTGCAAGCAGGATGTGTTTCTGCCTTGTGTTCTTATGA |
| GCTATTGCCTTCTGCGCCAATGGCTTGTTAATTGCTTGGTTCTTGCAAAATGCTTTGCGC |
| GCTGTTATTCAAGTTTCTACCTTCGCGGTTTTACTTGAGTGACGCTGCTCATGCTTGCAA |
| CCGCTGGGATGCAGGTGCATGCCTCTAGCATGAAGTCAGACAA |
| >GS_C15m (*Cladocopium*) |
| AATCAATGGCCTCCTGAACGTGCGTTGCACCCTTGGGATTTCCTGAGAGTATGTTTGCTT |
| CAGTGCTTAACTTGCCCCAACTTTGCAAGCAGGATGTGTTTCTGCCTTGCGTTCTTATGA |
| GCTATTGCCTTCTGCGCCAATGGCTTGTTAATTGCTTGGTTCTTGCAAAATGCTTTGCGC |
| GCTGTTATTCAAGTTTCTACCTTCGCGGTTTTACTTGAGTGACGCTGCTCATGCTTGCAA |
| CCGCTGGGATGCAGGTGCATGCCTCTAGCATGAAGTCAGACAA |
| >GS_C15.1 (*Cladocopium*) |
| AACCAATGGCCTCCTGAACGTGCGTTGCACCCTTGGGATTTCCTGAGAGTATGTCTGCTT |
| CAGTGCTTAACTTGCCCCAACTTTGCAAGCAGGATGTGCTTCTGCCTTGCGTTCTTATGA |
| GCTATTGCCTTCTGCGCCAATGGCTTGTTAATTGCTTGGTTCTTGCAAAATGCTTTGCGC |
| GCTGTTATTCAAGTTTCTACCTTCGCGGTTTTACTTGAGTGACGCTGCTCATGCTTGCAA |
| CCGCTGGGATGCAGGTGCATGCCTCTAGCATGAAGTCAGACAA |
| >GS_C15.2_(type_1) (*Cladocopium*) |
| AACCAATGGCTTCCTGAACGTGCGTTGCACCCTTGGGATTTCCTGAGAGTATGTCTGCTT |
| CAGTGCTTAACTTGCCCCAACTTTGCAAGCAGGATGTGTTTCTGCCTTGCGTTCTTATGA |
| GCTATTGCCTTCTGCGCCAATGGCTTGTTAATTGCTTGGTTCTTGCAAAATGCTTTGCGC |
| GCTGTTATTCAAGTTTCTACCTTCGCGGTTTTACTTGAGTGACGCTGCTCATGCTTGCAA |
| CCGCTGGGATGCAGGTGCATGCCTCTAGCATGAAGTCAGACAA |
| >GS_C15.2_(type_2) (*Cladocopium*) |
| AACCAATGGCCTCCTGAACGTGCGTTGCACCCTTGGGATTTCCTGAGAGTATGTCTGCTT |
| CAGTGCTTAACTTGCCCCAACTTTGCAAGCAGGATGTGTTTCTGCCTTGCGTGCTTATGA |
| CCTATTGCCTTCTGCGCCAATGGCTTGTTAATTGCTTGGTTCTTGCAAAATGCTTTGCGC |
| GCTGTTATTCAAGTTTCTACCTTCGCGGTTTTACTTGAGTGACGCTGCTCATGCTTGCAA |
| CCGCTGGGATGCAGGTGCATGCCTCTAGCATGAAGTCAGACAA |
| >GS_C15.3 (*Cladocopium*) |
| AACCAATGGCTTCCTGAACGTGCGTTGCACCCTTGGGATTTCCTGAGAGTATGTCTGCTT |
| CAGTGCTTAACTTGCCCCAACTTTGCAAGCAGGATGTGTTTCTGCCTTGCGTTCTTATGA |
| GCTATTGCCTTCTGCGCCAATGGCTTGTTAATTGCTTGGTTCTTGCAAAATGCTTTGCGC |
| GCTGTTATTCAAGTTTCTACCTTCGTGGTTTTACTTGAGTGACGCTGCTCATGCTTGCAA |
| CCGCTGGGATGCAGGTGCATGCCTCTAGCATGAAGTCAGACAA |
| >GS_C15.4 (*Cladocopium*) |
| AAGCAATGGCCTCCTGAACGTGCGTTGCACCCTTGGGATTTCCTGAGAGTATGTCTGCTT |
| CAGTGCTTAACTTGCCCCAACTTTGCAAGCAGGATGTGTTTCTGCCTTGCGTTCTTATGA |
| GCTATTGCCTTCTGCGCCAATGGCTTGTTAATTGCTTGGTTCTTGCAAAATGCTTTGCGC |
| GCTGTTATTCAAGTTTCTACCTTCGTGGTTTTACTTGAGTGACGCTGCTCATGCTTGCAA |
| CCGCTGGGATGCAGGTGCATGCCTCTAGCATGAAGTCAGACAA |
| >GS_C15.5 (*Cladocopium*) |
| AAGCAATGGCCTCCTGAACGTGCGTTGCACCCTTGGGATTTCCTGAGAGTATGTCTGCTT |
| CAGTGCTTAACTTGCCCCAACTTTGCAAGCAGGATGCGTTTCTGCCTTGCGTTCTTATGA |
| GCTATTGCCTTCTGCGCCAATGGCTTGTTAATTGCTTGGTTCTTGCAAAATGCTTTGCGC |
| GCTGTTATTCAAGTTTCTACCTTCGTGGTTTTACTTGAGTGACGCTGCTCATGCTTGCAA |
| CCGCTGGGATGCAGGTGCATGCCTCTAGCATGAAGTCAGACAA |
| >GS_C15.6 (*Cladocopium*) |
| AAGCAATGGCCTCCTGAACGTGCGTTGCACCCTTGGGATTTCCTGAGAGTATGTCTGCTT |
| CAGTGCTTAACTTGCCCCAACTTTGCAAGCAGGATGTGTTTCTGCCTTGCGTTCTTATGA |
| GCTATTGCCTTCTGCGCCAATGGCTTGTTAATTGCTTGGTTCTTGCAAAATGCTTTGCGC |
| GCTGTTATTCAAGTTTCTACCTTCGCGGTTTTACTTGAGTGACGCTGCTCATGCTTGCAA |
| CCGCTGGGATGCAGGTGCATGCCTCTAGCATGAAGTCAGACAA |
| >GS_C15.7 (*Cladocopium*) |
| AACCAATGGCCTCCTGAACGTGCGTTGCACCCTTGGGATTTCCTGAGAGTATGTCTGCTT |
| CAGTGCTTAACTTGCCCCAACTTTGCAAGCAGGATGTGTTTCTGCCTTGCGTTCTTATGA |
| GCTATTGCCTTCTGCGCCAATGGCTTGTTAATTGCTTGGTTCTTGCAAAATGCTTTGCGC |
| GCTGTTATTCAAGTTTCTACCTTCGCGGTTTTACTTGAGTGACGCTGCTCATGCTTGCAA |
| CCGCTGGGATGCAGGTGCATGCCTCTAGCATGAAGTCAGAGAA |
| >GS_C15.8 (*Cladocopium*) |
| AACCAATGGCCTCCTGAACGTGCGTTGCACCCTTGGGATTTCCTGAGAGTATGTCTGCTT |
| CAGTACTTAACTTGCCCCAACTTTGCAAGCAGGATGTGTTTCTGCCTTGCGTTCTTATGA |
| GCTATTGCCTTCTGCGCCAATGGCTTGTTAATTGCTTGGTTCTTGCAAAATGCTTTGCGC |
| GCTGTTATTCAAGTTTCTACCTTCGCGGTTTTACTTGAGTGACGCTGCTCATGCTTGCAA |
| CCGCTGGGATGCAGGTGCATGCCTCTAGCATGAAGTCAGACAA |
| >GS_C15.9 (*Cladocopium*) |
| AACCAATGGCCTCCTGAACGTGCGTTGCACCCTTGGGATTTCCTGAGAGTATGTCTGCTT |
| CAGTGCTTAACTTGCCCCAACTTTGCAAGCAGGATGTGTTTCTGCCTTGCGTTCTTATGA |
| GCTATTGCCTTCTGCGCCAATGGCTTGTTAATTGCTTGGTTCTTGCAAAATGCTTTGCGC |
| GCTGTTATTCAAGTTTCTACCTTTGCGGTTTTACTTGAGTGACGCTGCTCATGCTTGCAA |
| CCGCTGGGATGCAGGTGCATGCCTCTAGCATGAAGTCAGACAA |
| >GS_KB1 (*Cladocopium*) |
| AACCAATGGCGAAGGTGTGTTGCACCCTTGGGATTTCCTGAGAGTATGTCTGCTTCAGTG |
| CTTAACTTGCCCCAACTTTGCAAGCAGGATGTGTTTCTGCCTTGCGTTCTTATGAGCTAT |
| TGCCTTCTGCGCCAATGGCTTGGTAATTGCTTGGTTCTTGCAGAACGCTTTGCGTGCTGT |
| TATCATTTTCTACCTTCGCGGTTTTACTTGAGTGACGCTGCTCATGCTTGCAACCGCTGG |
| GATGCAGGTGCATGCCTCTAGCATGAAGTCAGACAA |
| >GS_KB2 (*Cladocopium*) |
| AACCAATGGCGAAGGTGTGTTGCACCTTTGGGATTTCCTGAGAGTATGTCTGCTTCAGTG |
| CTTAACTTGCCCCAACTTTGCAAGCAGGATGTGTTTCTGCCTTGCGTTCTTATGAGCTAT |
| TGCCTTCTGCGCCAATGGCTTGGTAATTGCTTGGTTCTTGCAGAACGCTTTGCGTGCTGT |
| TATTCCAGTTTCTACCTTCGCGGTTTTACTTGAGTGACGCTGCTCATGCTTGCAACCGCT |
| GGGATGCAGGTGCATGCCTCTAGCATGAAGTCAGACAA |
| >GS_KB3 (*Cladocopium*) |
| AACCAATGGCGAACGTGTGTTGCACCCTTGGGATTTCCTGAGAGTATGTCTGCTTCAGTG |
| CTTAACTTGCCCCAACTTTGCAAGCAGGATGTGTTTCTGCCTTGCGTTCTTATGAGCTAC |
| TGCCTTCTGCGCCAATGGCTTGGTAATTGCTTGGTTCTTGCAAAACGCTTTGCGTGCTGT |
| TATTCCAGTTTCTACCTTCGCGGTTTTACTTGAGTGACGCTGCTCATGCTTGCAACCGCT |
| GGGATGCAGGTGCATGCCTCTAGCATGAAGTCAGACAA |
| >GS_KB4 (*Cladocopium*) |
| AACCAATGGCGAACGTGTGTTGCACCCTTGGGATTTCCTGAGAGTATGTCTGCTTCAGTG |
| CTTAACTTGCCCCAACTTTGCAAGCAGGATGTGTTTCTGCCTTGCGTTCTTATGAGCTAC |
| TGGCTTCTGCGCCAATGGCTTGGTAATTGCTTGGTTCTTGCAAAACGCTTTGCGTGCTGT |
| TATTCCAGTTTCTACCTTCGCGGTTTTACTTGAGTGACGCTGCTCATGCTTGCAACCGCT |
| GGGATGCAGGTGCATGCCTCTAGCATGAAGTCAGACAA |
| >GS_C16 (*Cladocopium*) |
| AACCAATGGCCTCCTGAACGTGCGTTGCACTCTTGGGATTTCCTGAGAGTATGTCTGCTT |
| CAGTACTTAACTTGCCCCAACTTTGCAAGCAGGATGTGTTTCTGCCTTGCGTTCTTATGA |
| GCTATTGCCCTCTGAGCCAATGGCTTGTTAATTGCTTGGTTCTTGCAAACTGCTTTGCGC |
| GCTGTTATTCAAGTTTCTACCTTCGTGGTTTTACTTGAGTGACGCTGCTCATGCTTGCAA |
| CCGCTGGGATGCAGGTGCATGCCTCTAGCATGAAGTCAGACAA |
| >GS_C16a (*Cladocopium*) |
| AACCAATGGCCTCCTGAACGTGCGTTGCACTCTTGGGATTTCCTGAGAGTATGTCTGCTT |
| CAGTACTTAACTTGCCCCAACTTTGCAAGCAGGATGTGTTTCTGCCTTGCGTTCTTATGA |
| GCTATTGCCCTCTGAGCCAATGGCTTGTTAATTGCTTGGTTCTTGCAAACTGCTTTGCGC |
| GCTGTTATTCAAGTTTCTACCTTCGTGGTTTTACTTGAGTAACGCTGCTCATGCTTGCAA |
| CCGCTGGGATGCAGGTGCATGCCTCTAGCATGAAGTCAGACAA |
| >GS_C17a (*Cladocopium*) |
| AACCAATGGCCTCCTGAACGTGCGTTGCACTCTTGGGATTTCCTGAGAGTATGTCTGCTT |
| CAGCGCTTAACTTGCCCCAACTTTGCAAGCATTTCTGCCTTGCGTTCTTATGAGCTATTG |
| CCCTCTGAGTCAATGGCTTGTTAATTGCTTGGTTCTTGCAAAATGCTTTGCGCGCTGTTA |
| TTCAAGTTTCTACCTTCGTGGTTTTACTTGAGTGACGCTGCTCATGCTTGCAACCGCTGG |
| GATGCAGGTGCATGCCTCTAGCATGAAGTCAGACAA |
| >GS_C17=C17.2 (*Cladocopium*) |
| AACCAATGGCCTCCTGAACGTGCGTTGCACTCTTGGGATTTCCTGAGAGTATGTCTGCTT |
| CAGCGCTTAACTTGCCCCAACTTTGCAAGCATTTCTGCCTTGCGTTCTTATGAGCTATTG |
| CCCTCTCTGAGCCAATGGCTTGTTAATTGCTTGGTTCTTGCAAAATGCTTTGCGCGCTGT |
| TATTCAAGTTTCTACCTTCGTGGTTTTACTTGAGTGACGCTGCTCATGCTTGCAACCGCT |
| GGGATGCAGGTGCATGCCTCTAGCATGAAGTCAGACAA |
| >GS_C18 (*Cladocopium*) |
| AACCAATGGCCTCCTGAACGTGCGTTGCACTCTTGGGATTTCCTGAGAGTATGTCTGCTT |
| CAGTGCTTAACTTGCCCCAACTTTGCAAGCAGGATGTGTTTCTGCCTTGCGTTCTTATGA |
| GCTATTGCCCTCTGAGCCAATGGCTTGTTAATTGCTTGGTTCTTGCAAAATGCGTTGCGC |
| GCTGTTATTCAGGTTTCTACCTTCGTGGTTTTACTTGAGTGACGCTGCTCATGCTTGCAA |
| CCGCTGGGATGCAGGTGCATGCCTCTAGCATGAAGTCAGACAA |
| >GS_C19 (*Cladocopium*) |
| AACCAATGGCCTCCTGAACGTGCGTTGCACCCTTGGGATTTCCTGAGAGTATGTCTGCTT |
| CAGTGCTTAACTTGCTCCAACTTTGCAAGCAGGATGTGTTTCTGCCTTGCGTTCTTATGA |
| GCTATTGCCTTCTGGCCAATGGCTTGTTAATTGCTTGGTTCTTGCAAAATGCTTTGCGCG |
| CTGTTATTCAAGTTTCTACCTTCGCGGTTTTACTTGAGTGACGCTGCTCATGCTTGCAAC |
| CGCTGGGATGCAGGTGCATGCCTCTAGCATGAAGTCAGACAA |
| >GS_C20 (*Cladocopium*) |
| AACCAATGGCCTCCTGAACGTGCGTTGCACTCTTGGGTTTTCCTGAGAGTATGTCTGCTT |
| CAGTGCTTAACTTGCCCCAACTTTGCAAGCAGGATGTGTTTCTGCCTTGCGTTCTTATGA |
| GCTATTGCTCTCTGAGCCAATGGCTTGTCAATTGCTTGGTTCTTGCAAAATGCTTTGCGC |
| GCTGTTATTCAAGTTTCTACCTTCGTGGTTTTACTTGAGTGACGCTGCTCATGCTTGCAA |
| CCGCTGGGATGCAGGTGCATGCCTCTAGCATGAAGTCAGACAA |
| >GS_C21.11 (*Cladocopium*) |
| AACCAATGGCCTCCTGAACGTGCGTTGCACTCTTGGGATTTCCTGAGAGTATGTCTGCTT |
| CAGTGCTTAACTTGCCCCAACTTTGCAAGCATTTCTGCCTTGCGTTTTTATGAGCTATTG |
| CCCTCTGAGCCAATGGCTTGTTAATTGCTTGGTTCTTGCAAAATGCTTTGCGCGCTGTTA |
| TTCAAGTTTCTACCTTCGTGGTTTTACTTGAGTGACGCTGCTCATGCTTGCAACCGCTGG |
| GATGCAGGTGCATGCCTCTAGCATGAAGTCAGACAA |
| >GS_C21.12 (*Cladocopium*) |
| AACCAATGGCTTCGTGAACGTGCGTTGCACTCTTGGGATTTCCTGAGAGTATGTCTGCTT |
| CAGTGCTTAACTTGCCCCAACTTTGCAAGCATTTCTGCCTTGCGTTCTTATGAGCTATTG |
| CCCTCTGAGCCAATGGCTTGTTAATTGCTTGGTTCTTGCAAAATGCTTTGCGCGCTGTTA |
| TTCAAGTTTCTACCTTCGTGGTTTTACTTGAGTGACGCTGCTCATGCTTGCAACCGCTGG |
| GATGCAGGTGCATGCCTCTAGCATGAAGTCAGACAA |
| >GS_C21.13 (*Cladocopium*) |
| AACCAATGGCTTCGTGAACGTGCGTTGCACTCTTGGGATTTCCTGAGAGTATGTCTGCTT |
| CAGTGCTTAACTTGCCCCAACTTTGCAAGCATTTCTGCTTTGCGTTCTTATGAGCTATTG |
| CCCTCTGAGCCAATGGCTTGTTAATTGCTTGGTTCTTGCAAAATGCTTTGCGCGCTGTTA |
| TTCAAGTTTCTACCTTCGTGGTTTTACTTGAGTGACGCTGCTCATGCTTGCAACCGCTGA |
| GATGCAGGTGCATGCCTCTAGCATGAAGTCAGACAA |
| >GS_C21.14 (*Cladocopium*) |
| AACCAATGGCCTCCTGAACGTGCGTTGCACTCTTGGGATTTCCTGAGAGTATGTCTGCTT |
| CAGTGCTTAACTTGCCCCAACTTTGCAAGCATTTCTGCCTTGCGTTCTTATGAGCTATTG |
| CCCTCTGAGCCAATGGCTTGTTAATTGCTTGGTTCTTGCAAAATGCTTTGCGCGCTGTTA |
| TTCAAGTTTCTACCTTCGTGGTTTTACTTGAGTGACGCTGCTCATGCTTGCAACTGCTGG |
| GATGCAGGTGCATGCCTCTAGCATGAAGTCAGACAA |
| >GS_C21.16 (*Cladocopium*) |
| AACCAATGGCCTCCTGAACGTGCGTTGCACTCTTGGGATTTCCTGAGAGTATGTCTGCTT |
| CAGTGCTTAACTTGCCCCAACTTTGCAAGCATTTCTGCCTTGCGTTCTTATGAGCTATTG |
| CCCTCTGAGCCAATGGCTTGTTAATTGCTTGGTTCTTGCAAAATGCTTTGCGCGCTGTTA |
| TTCAAGTTTCTACCTTCGTGGTTTTACTTGAGTGACGTTGCTCATGCTTGCAACCGCTGG |
| GATGCAGGTGCATGCCTCTAGCATGAAGTCAGACAA |
| >GS_C21a (*Cladocopium*) |
| AACCAATGGCCTCCTGAACGTGCGTTGCACTCTTGGGATTTCCTGAGAGGATGTCTGCTT |
| CAGTGCTTAACTTGCCCCAACTTTGCAAGCATTTCTGCCTTGCGTTCTTATGAGCTATTG |
| CCCTCTGAGCCAATGGCTTGTTAATTGCTTGGTTCTTGCAAAATGCTTTGCGCGCTGTTA |
| TTCAAGTTTCTACCTTCGTGGTTTTACTTGAGTGACGCTGCTCATGCTTGCAACCGCTGG |
| GATGCAGGTGCATGCCTCTAGCATGAAGTCAGACAA |
| >GS_C22 (*Cladocopium*) |
| AACCAATGGCCTCCTGAACGTGCGTTGCACTCTTGGGATTTCCTGAGAGTATGTCTGCTT |
| CAGTGCTTAACTTGCCCCAACTTTGCAAGCAGGATGTGTTTCTGCCTTGCGTTCTTATGA |
| GCTATTGCCCTCTGAGCCAATGGCTTGTTAATTGCTTGGTTCTTGCAAAATGCTTTGCGC |
| GCTGTTATTCAAGTTTCTACCTTCGTGGTTTTACTTGAGTCTCTCATGCTTGCAACCGCT |
| GGGATGCAGGTGCATGCCTCTAGCATGAAGTCAGACAA |
| >GS_C22a (*Cladocopium*) |
| AACCAATGGCCTCCTGAACGTGCGTTGCACTCTTGGGATTTCCTGAGAGTATGTCTGCTT |
| CAGTGCTTAACTTGCCCCAACTTTGCAAGCAGGATGTGTTTCTGCCTTGCGTTCTTATGA |
| GCTATTGCCCTCTGAGCCAATGGCTTGTTAATTGCTTGGTTCTTGCAAAATGCTTTGCGC |
| GCTGTTATTCAAGTTTCCACCTTCGTGGTTTTACTTGAGTCTCTCATGCTTGCAACCGCT |
| GGGATGCAGGTGCATGCCTCTAGCATGAAGTCAGACAA |
| >GS_C23 (*Cladocopium*) |
| AACCAATGGCCTCCTGAACGTGCGTTGCACTCTTGGGATTTCCTGAGAGTATGTCTGCTT |
| CAGTGCTTAACTTGCCCCAACTTTGCAAGCAGGATGTGTGTCTGCCTTGCGTTCTTATGC |
| GCTATTGCCCTCTGAGCCAATGGCTTGTTAATTGCTTGGTTCTTGCAAAATGCTTTGCGC |
| GCTGTTATTCAGGTTTCTACCTTCGTGGTTTTACTTGAGTGACGCTGCTCATGCTTGCAA |
| CCGCTGGGATGCAGGTGCATGCCTCTAGCATGAAGTCAGACAA |
| >GS_C24 (*Cladocopium*) |
| AACCAATGGCCTCCTGAACGTGCGTTGCACTCTTGGGATTTCCTGAGAATATGTCTGCTT |
| CAGTGCTTAACTTGCCCCAACTTTGCAAGCAGGATGTGTTTCTGCCTTGCGTTCTTATGA |
| GCTATTGCCCTCTGAGCCAATGGCTTGTTAATTGCTTGGTTCTTGCAAAATGCTTTGCGC |
| GCTGTTATTCAGGTTTCTACCTTCGTGATTTTACTTGAGTGACGCTGCTCATGCTTGCAA |
| CCGCTGGGATGCAGGTGCATGCCTCTAGCATGAAGTCAGACAA |
| >GS_C25 (*Cladocopium*) |
| AATCAATGGCCTCCTGAACGTTCGTTGCACTCTTGGGATTTCCTGAGAGTATGTCTGCTT |
| CAGTGCTTAACTTGCCCCAACTTTGCAAGCAGGATGTGTTTCTGCCTTGCGCTCTTATGA |
| GTCATTGCCCTCTGAGCCAATGGCTTGTTAATTGCTTGGCTCTTGCAAAATGCTTTGCGC |
| GCTGTTATTCACGTTTCTACCTTCGTGGTTTTACTTGAGTGACACGCTGCTCATGCTTGC |
| AACCGCTGGGATGCAGGTGCATGCCTCTAGCATGAAGTCAGACAA |
| >GS_C26=C26a=C35a_(type_2) (*Cladocopium*) |
| AATCAATGGCCTCCTGAACGTGCGTTGCACTCTTGGGATTTCCTGAGAGTATGTCTGCTT |
| CAGCGCTTAACTTGCCCCAACTTTGCAAGCATTTCTGCCTTGCGTTCTTATGAGCTATTG |
| CCTCTCTGAGCCAATGGCTTGTTAATTGCTTGGTTCTTGCAAAATGCTTTGCGCGCTGTT |
| ATTCAAGTTTCTACCTTCGTGGTTTTACTTGAGTGACGCTGCTCATGCTTGCAACCGCTG |
| GGATGCAGGTGCATGCCTCTAGCATGAAGTCAGACAA |
| >GS_C21.b1 (*Cladocopium*) |
| AACCAATGGCCTCCTGAACGTGCGTTGCACTCTTGGGATTTCCTGAGAGTATGTCTGCTC |
| CAGTGCTTAACTTGCCCCAACTTTGCAAGCATTTCTGCCTTGCGTTCTTATGAGCTATTC |
| CCCTCTGAGCCAATGGCTTGTTAATTGCTTGGTTCTTGCAAAATGCTTTGCGCGCTGTTA |
| TTCAAGTTTCTACCTTCGTGGTTTTACTTGAGTGACGCTGCTCATGCTTGCAACCGCTGG |
| GATGCAGGTGCATGCCTCTAGCATGAAGTCAGACAA |
| >GS_C26.b1=C35_(type_2) (*Cladocopium*) |
| AACCAATGGCCTCCTGAACGTGCGTTGCACTCTTGGGATTTCCTGAGAGTATGTCTGCTT |
| CAGCGCTTAACTTGCCCCAACTTTGCAAGCATTTCTGCCTTGCGTTCTTATGAGCTATTG |
| CCTCTCTGAGCCAATGGCTTGTTAATTGCTTGGTTCTTGCAAAATGCTTTGCGCGCTGTT |
| ATTCAAGTTTCTACCTTCGTGGTTTTACTTGAGTGACGCTGCTCATGCTTGCAACCGCTG |
| GGATGCAGGTGCATGCCTCTAGCATGAAGTCAGACAA |
| >GS_C26.b2 (*Cladocopium*) |
| AACCAATGGCCTCCTGAACGTGCGTTGCACTCTTGGGATTTCCTGAGAGTATGTCTGCTT |
| CAGCGCTTAACTTGCCTCAACTTTGCAAGCATTTCTGCCTTGCGTTCTTATGAGCTATTG |
| CCTCTCTGAGCCAATGGCTTGTTAATTGCTTGGTTCTTGCAAAATGCTTTGCGCGCTGTT |
| ATTCAAGTTTCTACCTTCGTGGTTTTACTTGAGTGACGCTGCTCATGCTTGCAACCGCTG |
| GGATGCAGGTGCATGCCTCTAGCATGAAGTCAGACAA |
| >GS_C26.b3 (*Cladocopium*) |
| AATCAATGGCTTCCTGAACGTGCGTTGCACTCTTGGGATTTCCTGAGAGTATGTCTGCTT |
| CAGCGCTTAACTTGCCCCAACTTTGCAAGCATTTCTGCCTTGCGTTCTTATGAGCTATTG |
| CCTCTCTGAGCCAATGGCTTGTTAATTGCTTGGTTCTTGCAAAATGCTTTGCGCGCTGTT |
| ATTCAAGTTTCTACCTTCGTGGTTTTACTTGAGTGACGCTGCTCATGCTTGCAACCGCTG |
| GGATGCAGGTGCATGCCTCTAGCATGAAGTCAGACAA |
| >GS_C27=GS_C30_(type_1) (*Cladocopium*) |
| AACCAATGGCCTCCTGAACGTGCGTTGCACTCTTGGGATTTCCTGAGAGTATGTCTGCTT |
| CAGTGCTTAACTTGCCCCAACTTTGCAAGCATTTCTGCCTTGCGTTCTTATGAGCTATTG |
| CCCCTCTGAGCCGATGGCTTGTTAATTGCTTAGTTCTTGCAAAATGCTTTGCGCGCTGTT |
| ATTCAAGTTTCTACCTTCGTGGTTTTACTTGAGTGACGCTGCTCATGCTTGCAACCGCTG |
| GGATGCAGGTGCATGCCTCTAGCATGAAGTCAGACAA |
| >GS_C27.1 (*Cladocopium*) |
| AACCAATGGCCTCCTGAACGTGCGTTGCACTCTTGGGATTTCCTGAGAGTATGTCTGCTT |
| CAGTGCTTAACTTGCCCCAACTTTGCAAGCATTTCTGCCTTGCGTTCTTATGAGCTATTG |
| CCCCTCTGAGCCGATGGCTTGTTAATTGCTTAGTTCTTGCAAAATGCTTTGCGCGCTGTT |
| ATTCAAGTTTCTACCTTCGTGGTTTTACTTGAGTGACGCTGCTCATGCTTGCAACCGCTG |
| GGATGCAGGTGCATGCCTCTAGCATGAAGTTAGACAA |
| >GS_C28 (*Cladocopium*) |
| AACCAATGGCCTCCTGAACGTGCGTTGCACTCTTGGGATTTCCTGAGAGTATGTCTGCTT |
| CAGTGCTTAACTTTTGCCCCAACTTTGCAAGCAGGATGTGTTTCTGCCTTGCGTTCATAT |
| GAGCTATTGCCCTCTGAGCCAATGGCTTGTTAATTGCTTGGTTCTTGCAAAATGCTTTGC |
| GCGCTGTTATTCAAGTTTCTACCTTCGTGGTTTTACTTGAGTGACGCTGCTCATGCTTGC |
| AACCGCTGGGATGCAGGTGCATGCCTCTAGCATGAAGTCAGACAA |
| >GS_C29 (*Cladocopium*) |
| AACCAATGGCCTCCTGAACGTGCGTTGCACTCTTGGGATTTCCTGAGAGTATGTCTGCTT |
| CAGTGCTTAACTTGCCCCAACTTTGCAAGCAGGATGTGTTTCTGCCTTGCGTTCTTATGA |
| GCTATTGCCCTCTGAGCCAATGGCTTGTTAATTGCTTGGCTCTTGCAAAATGCTTTGCGC |
| GCTGTTATTCAAGTTTCTACCTTCGTGGTTTTACTTGAGTGACGCTGCTCATGCTTGCAA |
| CCGCTGGGATGCAGGTGCATGCCTCTAGCATGAAGTCAGACAA |
| >GS_C31d=C30_(type_2) (*Cladocopium*) |
| AACCAATGGCCTCCTGAACGTGCGTTGCACTCTTGGGATTTCCTGAGAGTATGTCTGCTT |
| CAGCGCTTAACTTGCCCCAACTTTGCAAGCACTCTTCTGCCTTGCGTTCTTATGAGCTAT |
| TGCCCTCTCTGAGCCAATGGCTTGTTAATTGCTTGGTTCTTGCAAAATGCTTTGCGCGCT |
| ATTATTCAAGTTTCTACCTTCGTGGTTTTACTTGAGTGACGCTGCTCATGCTTGCAACCG |
| CTGGGATGCAGGTGCATGCCTCTAGCATGAAGTCAGACAA |
| >GS_C31 (*Cladocopium*) |
| AACCAATGGCCTCCTGAACGTGCGTTGCACTCTTGGGATTTCCTGAGAGTATGTCTGCTT |
| CAGCGCTTAACTTGCCCCAACTTTGCAAGCACTCTTCTGCCTTGCGTTCTTATGAGCTAT |
| TGCCCTCTCTGAGCCAATGGCTTGTTAATTGCTTGGTTCTTGCAAAATGCTTTGCGCGCT |
| GTTATTCAAGTTTCTACCTTCGTGGTTTTACTTGAGTGACGCTGCTCATGCTTGCAACCG |
| CTGGGATGCAGGTGCATGCCTCTAGCATGAAGTCAGACAA |
| >GS_C31a (*Cladocopium*) |
| AATCAATGGCCTCCTGAACGTGCGTTGCACTCTTGGGATTTCCTGAGAGTATGTCTGCTT |
| CAGCGCTTAACTTGCCCCAACTTTGCAAGCACTCTTCTGCCTTGCGTTCTTATGAGCTAT |
| TGCCCTCTCTGAGCCAATGGCTTGTTAATTGCTTGGTTCTTGCAAAATGCTTTGCGCGCT |
| GTTATTCAAGTTTCTACCTTCGTGGTTTTACTTGAGTGACGCTGCTCATGCTTGCAACCG |
| CTGGGATGCAGGTGCATGCCTCTAGCATGAAGTCAGACAA |
| >GS_C31c (*Cladocopium*) |
| AACCAATGGCCTCCTGAACGTGCGTTGCACTCTTGGGATTTCCTGAGAGTATGTCTGCTT |
| CAGCGCTTAACTTGCCCCAACTTTGCAAGCACTTCTGCCTTGCGTTCTTATGAGCTATTG |
| CCCTCTCTGAGCCAATGGCTTGTTAATTGCTTGGTTCTTGCAAAATGCTTTGCGCGCTGT |
| TATTCAAGTTTCTACCTTCGTGGTTTTACTTGAGTGACGCTGCTCATGCTTGCAACCGCT |
| GGGATGCAGGTGCATGCCTCTAGCATGAAGTCAGACAA |
| >GS_C31.1 (*Cladocopium*) |
| AACCAATGGCCTCCTGAACGTGCGTTGCACTCTTGGGATTTCCTGAGAGTATGTCTGCTT |
| CAGCGCTTAACTTGCCCCAACTTTGCAAGCACTCTTCTGCCTTGCGTTCTTATGAGCTAT |
| TGCCCTCTCTGAGCCAATGGCTTGTTAATTGCTTGATTCTTGCAAAATGCTTTGCGCGCT |
| GTTATTCAAGTTTCTACCTTCGTGGTTTTACTTGAGTGACGCTGCTCATGCTTGCAACCG |
| CTGGGATGCAGGTGCATGCCTCTAGCATGAAGTCAGACAA |
| >GS_C31.5 (*Cladocopium*) |
| AACCAATGGCCTCCTGAACGTGCGTTGCACTCTTGGGATTTCCTGAGAGTATGTCTGCTT |
| CAGCGCTTAACTTGCCCCAACTTTGCAAGCACTCTTCTGCCTTGCGTTCTTATGAGCTAT |
| TGCCCTCTCTGAGCCAATGGCTTGTTAATTGCTTGGTTCTTGCAAAATGCTTTGCGCGCT |
| GTTATTCAAGTTTCTACTTTCGTGGTTTTACTTGAGTGACGCTGCTCATGCTTGCAACCG |
| CTGGGATGCAGGTGCATGCCTCTAGCATGAAGTCAGACAA |
| >GS_C31.6 (*Cladocopium*) |
| AACCAATGGCCTCCTGAACGTGCGTTGCACTCTTGGGATTTCCTGAGAGTATGTCTGCTT |
| CAGCGCTTAACTTGCCCCAACTTTGCAAGCACTCTTCTGCCTTGCGTTCTTATGAGCTAT |
| TGCCCTCTCTGAGCCAATGGCTTGTTAATTGCTTGGTTCTTGCAAAATGCTTTGCGCGCT |
| GTTATTCAAGTTTCTACCTTCGTGGTTTTACTTGAGTGACGCTGCTCATGCTTGCAACCG |
| CTGGGATGCGGGTGCATGCCTCTAGCATGAAGTCAGACAA |
| >GS_C31.9 (*Cladocopium*) |
| AACCAATGGCCTCCTGAACGTGGGTTGCACTCTTGGGATTTCCTGAGAGTATGTCTGCTT |
| CAGCGCTTAACTTGCCCCAACTTTGCAAGCATCTTCTGCCTTGCGTTCTTATGAGCTATT |
| GCCCTCTCTGAGCCAATGGCTTGTTAATTGCTTGGTTCTTGCAAAATGCTTTGCGCGCTG |
| TTATTCAAGTTTCTACCTTCGTGGTTTTACTTGAGTGACGCTGCTCATGCTTGCAACCGC |
| TGGGATGCAGGTGCATGCCTCTAGCATGAAGTCAGACAA |
| >GS_C31.10 (*Cladocopium*) |
| AACCAATGGCCTCCTGAACGTGCGTTGCACTCTTGGGATTTCCTGAGAGTATGTCTGCTT |
| CAGCGCTTAACTTGCCCCAACTTTGCAAGCATCTTCTGCCTTGCGTTCTTATGAGCTATT |
| GCCCTCTCTGAGCCAATGGCTTGTTAATTGCTTGGTTCTTGCAAAATGCTTTGCGCGCTG |
| TTATTCAAGTTTCTACCTTCGTGGTTTTACTTGAGTGACGCTGCTCATGCTTGCAACCGC |
| TGGGATGCAGGTGCATGCCTCTAGCATGAAGTCAGACAA |
| >GS_C32 (*Cladocopium*) |
| AACCAATGGCCTCCTGAACGTGCGTTGCACTCTTGGGATTTCCTGAGAGTATGTCTGCTT |
| CAGCGCTTAACTTGCCCCAACTTTGCAAGCATTTCTGCCTTGCGTTTTTATGAGCTATTG |
| CCCTCTGAGCCAATGGCTTGTTAATTGCTTGGTTCTTGCAAAATGCTTTGCGCGCTGTTA |
| TTCAAGTTTCTACCTTTGTGGTTTTACTTGAGTGACGCTGCTCATGCTTGCGACCGCTGG |
| GATGCAGGTGCATGCCTCTAGCATGAAGTCAGACAA |
| >GS_C33a=C33_(type_1) (*Cladocopium*) |
| AATCAATGGCCTCCTGAACGTGCGTTGCACTCTCGGGATTTCCTGAGAGCATGTCTGCTT |
| CAGTGCTTACCTTGCCCCAACTTTGCAAGCAGGATGTGTTTCTGCCTTGCGTTCTTATGA |
| GTTATTGCCCTCTGAGGCAATGGCTTGTTAATTGCTTGGTTCTTGCAAAATGCTTTGCGC |
| GCTGTTATTCAGGTTTCTACCTTCGTGGTTTTACTTGAGTGACGCTGCTCATGCTTGCAA |
| CCGCTGGGATGCAGGTGCATGCCTCTAGCATGAAATCAGACAA |
| >GS_C33_(type_2) (*Cladocopium*) |
| AATCAATGGCCTCCTGAACGTGCGTTGCACTCTCGGGATTTCCTGAGAGCATGTCTGCTT |
| CAGTGCTTACCTTGCCCCAACTTTGCAAGCAGGATGTGTTTCTGCCTTGCGTTCTTATGA |
| GTTATTGCCCTCTGAGGCAATGGCTTGTTAATGGCTTGGTTCTTGCAAAATGCTTTGCGC |
| GCTGTTATTCAGGTTTCTACCTTCGTGGTTTTACTTGAGTGACGCTGCTCATGCTTGCAA |
| CCGCTGGGATGCAGGTGCATGCCTCTAGCATGAAATCAGACAA |
| >GS_C33.1 (*Cladocopium*) |
| AACCAATGGCCTCCTGAACGTGCGTTGCACTCTTGGGATTTCCTGAGAGTATGTCTGCTT |
| CAGTGCTTAACTTGCCCCAACTTTGCAAGCAGGATGTGTTTCTGCCTTGCGTTCTTATGA |
| GCTATTGCCCTCTGAGCCAATGGCTTGTTAATTGCTTGGGTCTTGCAAAATGCTTTGCGC |
| GCTGTTATTCAGGTTTCTACCTTCATGGTTTTACTTGAGTGACGCTGCTCATGCTTGCAA |
| CCGCTGGGATGCAGGTGCATGCCTCTAGCATGAAGTCAGACAA |
| >GS_C34 (*Cladocopium*) |
| AACCAATGGCCTCCTGAACGTGCGTTGCACTCTCGGGATTTCCTGAGAGCATGTCTGCTT |
| CAGTGCTTAACTTGCCCCAACTTTGCAAGCAGGATGTGTTTCTGCCTTGCGTTCTTATGA |
| GTTATTGCCCTCTGAGGCAATGGCTTGTTAATTGCTTGGTTCTTGCAAAATACTTTGCGC |
| GCTGTTATTCAGGTTTCTACCTTCGTGGTTTTACTTGAGTGACGCTGCTCATACTTGCAA |
| CCGCTGGGATGCAGGTGCATGCCTCTAGCATGAAGTCAGACAA |
| >GS_C35_(type_1)=C35a_(type_1) (*Cladocopium*) |
| AACCAATGGCCTCCTGAACGTGCGTTGCACTCTTGGGATTTCCTGAGAGTATGTCTGCTT |
| CAGTGCTTACCTTGCCCCAACTTTGCAAGCAGGATGTGTTTCTGCCTTGCGTTCTTATGA |
| GCTATTGGCCTCTGAGCCAATGGCTTGTTAATTGCTTGGTTCTTGCAAAATGCTTTGCGC |
| GCTGTTATTCAAGTGTCTACCTTCGTGGTTTTACTTGAGTGACACTGCTCATGCTTGCAA |
| CCGCTGGGATGCAGGTGCATGCCTCTAGCATGAAGTCAGACAA |
| >GS_C35_(type_3) (*Cladocopium*) |
| AACCAATGGCCTCCTGAACGTGCGTTGCACTCTTGGGATTTCCTGAGAGTATGTCTGCTT |
| CAGTGCTTAACTTGCCCCAACTTTGCAAGCAGGATGTGTTTCTGCCTTGCGTTCTTATGA |
| GCTATTGGCCTCTGAGCCAATGGCTTGTTAATTGCTTGGTTCTTGCAAAATGCTTTGCGC |
| GCTGTTATTCAAGTGTCTACCTTCGTGGTTTTACTTGAGTGACACTGCTCATGCTTGCAA |
| CCGCTGGGATGCAGGTGCATGCCTCTAGCATGAAGTCAGACAA |
| >GS_C36 (*Cladocopium*) |
| AACCAATGGCCTCCTGAACGTGCGTTGCACTCTTGGGATTTCCTGAGAGTATGTCTGCTT |
| CAGTGCTTAACTTGCCCCAACTTTGCAAGCAGGATGTGTTTCTGCCTTGCGTTCTTATGA |
| GCTATTGCCCTCTGAGCCAATGGCTTGTTAATTGCTTGGTTCTTGCAAAATGCTTTGCGC |
| GCTGTTATTCAAGTTTCTACCTTCGTGGTTTTACTTGAGTGACGCTGCTCATGCTTGCAA |
| CCGCCGGGATGCAGGTGCATGCCTCTAGCATGAAGTCAGACAA |
| >GS_C37 (*Cladocopium*) |
| AACCAATGGCCTCCTGAACGTGCGTTGCACTCTTGGGATTTCCTGAGAGTATGTCTGCTT |
| CAGTGCTTAACTTGTCCCAACTTTGCAAGCAGGATGTGTTTCTGCCTTGCGTTCTTATGA |
| GCTATTGCCCTCTGAGCCAATGGCTTGTTAATTACTTGGTTCTTGCAAAATGCTTTGCGC |
| GCTGTTATTCAGGTTTCTACCTTCGTGGTTTTACTTGAGTGACGCTGCTCGTGCTTGCAA |
| CCGCTGGGATGCAGGTGCATGCCTCTAGCATGAAGTCAGACAA |
| >GS_C38 (*Cladocopium*) |
| AACCAATGGCCTCCTGAACGTGCGTTGCACTCTTGGGATTTCCTGAGAGTATGTCTGCTT |
| CAGTGCTTAACTTGCCCCAACTTTGCAAGCAGGATGTGTTTCTGCCTTGTGTTCTTATGA |
| GCTATTGCCCTCTGAGCCAATGGCTTGTTAATTGCTTGGTTCTTGCAAAATGCTTTGCGC |
| GCTGTTATTCAAGTTTCTACCTTCGTGGTTTTACTTGAGTGACGCTGCACATGCTTGCAA |
| CCGCTGGGATGCAGGTGCATGCCTCTAGCATGAAGTCAGACAA |
| >GS_C38a (*Cladocopium*) |
| AACCAATGGCCTCCTGAACGTGCGTTGCACTCTTGGGATTTCCTGAGAGTATGTCTGCTT |
| CAGTGCTTAACTTGCCCCAACTTTGCAAGCAGGATGTGTTTCTGCCTTGTGTTCTTATGA |
| GCTATTGCCCTCTGAGCCAATGGCTTGTTAATTGCTTGGTTCTTGCAAAATGCTTTGCGC |
| GCTGTTATTCAAGTTTCTACCCTTTGTGGTTTTACTTGAGTGACGCTGCTCATGCTTGCA |
| ACCGCTGGGATGCAGGTGCATGCCTCTAGCATGAAGTCAGACAA |
| >GS_C39 (*Cladocopium*) |
| AACCAATGGCCTCCTGAACGTGCGTTGCACTCTTGGGATTTCCTGAGAGTATGTCTGCTT |
| CAGTGCTTAACTTGCCCCAACTTTGCAAGCAGGATGTGTTTCTGCCTTGCGTTCTTATGA |
| GCTATTGCCCTCTGAGCCAATGGCTTGTTAATTGCTTGGTTCTTGCAAAATGCTTTGCGC |
| GCTGTTATTCAGGTTTTTACTTGAGTGACGCTGCTCATGCTTGCAACCGCTGGGATGCAG |
| GTGCATGCCTCTAGCATGAAGTCAGACAA |
| >GS_C40 (*Cladocopium*) |
| AACCAATGGCCTCCTGAACGTGCGTTGCACTCTTGGGATTTCCTGAGAGTATGTCTGCTT |
| CAGTGCTTAACTTGCCCCAACTTTGCAAGCAGGATGTGTTTCTGCCTTGCGTTCTTATGA |
| GCTATTGCCCTCTGAGCCAATGGCTTGTTAATTGCTTGGTTCTGGCAAAATGCTTTGCGC |
| GCTGTTATTCAAGTTTCTACCTTCGTGGTTTTACTTGAGTGACGCTGCTCATGCTTGCGA |
| CCGCTGGGATGCAGGTGCATGCCTCTAGCATGAAGTCAGACAA |
| >GS_C40a (*Cladocopium*) |
| AACCAATGGCCTCCTGAACGTGCGTTGCACTCTTGGGATTTCCTGAGAGTATGTCTGCTT |
| CAGTGCTTAACTTGCCCCAACTTTGCAAGCAGGATGTGTTTCTGCCTTGCATTCTTATGA |
| GCTATTGCCCTCTGAGCCAATGGCTTGTTAATTGCTTGGTTCTGGCAAAATGCTTTGCGC |
| GCTGTTATTCAAGTTTCTACCTTCGTGGTTTTACTTGAGTGACGCTGCTCATGCTTGCGA |
| CCGCTGGGATGCAGGTGCATGCCTCTAGCATGAAGTCAGACAA |
| >GS_C40b (*Cladocopium*) |
| AACCAATGGCCTCCTGAACGTGCGTTGCACTCTTGGGATTTCCTGAGAGTATGTCTGCTT |
| CAGTGCTTAACTTGCCCCAACTTTGCAAGCAGGATGTGTTTCTGCCTTGCGTTCTTATGA |
| GCTATTGCCCTCTGAGCCAATGGCTTGGTTAATTGCTTGGTTCTGGCAAAATGCTTTGCG |
| CGCTGTTATTCAAGTTTCTACCTTCGTGGTTTTACTTGAGTGACGCTGCTCATGCTTGCG |
| ACCGCTGGGATGCAGGTGCATGCCTCTAGCATGAAGTCAGACAA |
| >GS_C41 (*Cladocopium*) |
| AACCAATGGCCTCCTGAACGTGCGTTGCACTCTTGGGATTTCCTGAGAGTATGTCTGCTT |
| CAGTGCTTAACTTGCCCCAACTTTGCAAGCAGGATGTGTTTCTGCCTTGCGTTCTTATGA |
| GCTATTGCCCTCTGAGCCAATGGCTTGTTAATTGCTTGGTTCTTGCAAAATGCTTTGCGC |
| GCTGTTATTCAGGTTTCTACCTTCGTGATTTTACTTGAGTGACGCTGCTCATGCTTGCAA |
| CCGCTGGGATGCAGGTGCATGCCTCTAGCATGAAGTCAGACAA |
| >GS_C42a=C42_(type_1) (*Cladocopium*) |
| AATCAATGGCCTCCTGAACGTGCGTTGCACTCTTGGGATTTCCTGAGAGTATGTCTGCTT |
| CAGTGCTTAACTTGCCCCAACTTTGCAAGCAGGATGTGTTTCTGCCTTGCGTTCTTATGC |
| GCTATTGCCCTCTGAGCCAATGGCTTGTGAATTGCTTGGTTCTTGCAAAATGCTTTGCGC |
| GCTGTTATTCAGGTTTCTACCTTCGTGGTTTTACTTGAGTGACGCTGCTCATGCTTGCAA |
| CCGCTGGGATGCAGGTGCATGCCTCTAGCATGAAGTCAGACAA |
| >GS_C42_(type_2) (*Cladocopium*) |
| AATCAATGGCCTCCTGAACGTGCGTTGCACTCTTGGGATTTCCTGAGAGTATGTCTGCTT |
| CAGTGCTTAACTTGCCCCAACTTTGCAAGCAGGATGTGTTTCTGCCTTGCGTTCTTATGA |
| GCTATTGCCCTCTGAGCCAATGGCTTGTGAATTGCTTGGTTCTTGCAAAATGCTTTGCGC |
| GCTGTTATTCAGGTTTCTACCTTCGTGGTTTTACTTGAGTGACGCTGCTCATGCTTGCAA |
| CCGCTGGGATGCAGGTGCATGCCTCTAGCATGAAGTCAGACAA |
| >GS_C42b (*Cladocopium*) |
| AATCAATGGCCTCCTGAACGTGCGGTGCACTCTTGGGATTTCCTGAGAGTATGTCTGCTT |
| CAGTGCTTAACTTGCCCCAACTTTGCAAGCAGGATGTGTTTCTGCCTTGCGTTCTTATGC |
| GCTATTGCCCTCTGAGCCAATGGCTTGTGAATTGCTTGGTTCTTGCAAAATGCTTTGCGC |
| GCTGTTATTCAGGTTTCTACCTTCGTGGTTTTACTTGAGTGACGCTGCTCATGCTTGCAA |
| CCGCTGGGATGCAGGTGCATGCCTCTAGCATGAAGTCAGACAA |
| >GS_C43 (*Cladocopium*) |
| AATCAATGGCCTCCTGAACGTGCGTTGCACTCTTGGGATTTCCTGAGAGTATGTCTGCTT |
| CAGTGCTTAACTTGCCCCAACTTTGCAAGCAGGATGTGTTTCTGCCTTGCGTTCTTATGA |
| GTTATTGTCCTCTGAGCCAATGGCTTGTGAATTGCTTGGTTCTTGCAAAATGCTTTGCGC |
| GCTGTTATTCAGGTTTCTACCTTCGTGGTTTTACTTGAGTGACGCTGCTCATGCTTGCAA |
| CCGCTGGGATGCAGGTGCATGCCTCTAGCATGAAGTCAGACAA |
| >GS_C44 (*Cladocopium*) |
| AACCAATGGCCTCCTGAACGTGCGTTGCACTCTTGGGATTTCCTGAGAGTATGTCTGCTT |
| CAGTGCTTAACTTGCCCCAACTTTGCAAGCAGGATGTGTTTCTGCCTTGCGTTCTTATGA |
| GCTATTGCCCTCTGAGCCAATGGCTTGTGAATTGCTTGGTTCTTGCAAAATGCTTTGTGC |
| GCTGTTATTCAGGTTTCTACCTTCGTGGTTTTACTTGAGTGACGCTGCTCATGCTTGCAA |
| CCGCTGGGATGCAGGTGCATGCCTCTAGCATGAAGTCAGACAA |
| >GS_C44a (*Cladocopium*) |
| AACCAATGGCCTCCTGAACGTGCGTTGCACTCTTGGGATTTCCTGAGAGTATGTCTGCTT |
| CAGTGCTTAACTTGCCCCAACTTTGCAAGCAGGATGTGTTTCTGCCTTGCGTTCTTATGA |
| GCTATTGCCCTCTGAGCCAATGGCTTGTGAATTGCTTGGTTCTTGCAAAATGCTTTGTGC |
| GCTGTTATTCAGGTTTCTACCTTCGTGGTTTTACTTGAGTGATGCTGCTCATGCTTGCAA |
| CCGCTGGGATGCAGGTGCATGCCTCTAGCATGAAGTCAGACAA |
| >GS_C45.3 (*Cladocopium*) |
| AACCAATGGCCTCCTGAACGTGCGTTGCACTCTTGGGATTTCCTGAGAGTATGTCTGCTT |
| CAGTGCTTAACTTGCCCCAACTTTGCAAGCAGGATGTGCTTCTGCCTTGCGTTCTTATGA |
| GCTATTGCCCTCTGAGCCAATGGCTTGTGAATTGCTTGGTTCTTGCAAAATGCTTTGCGC |
| GCTGTTATTCAGGTTTCTACCTTCGTGGTTTTACTTGAGTGACGCTGCTCATGCTTGCAA |
| CCGCTGGGATGCAGGTGCATGCCTCTAGCATGAAGTCAGACAA |
| >GS_C45a (*Cladocopium*) |
| AACCAATGGCCTCCTGAACGTGCGTTGCACTCTTGGGATTTCCTGAGAGTATGTCTGCTT |
| CAGTGCTTAACTTGCTCCAACTTTGCAAGCAGGATGTGTTTCTGCCTTGCGTTCTTATGA |
| GCTATTGCCCTCTGAGCCAATGGCTTGTGAATTGCTTGGTTCTTGCAAAATACTTTGTGC |
| GCTGTTATTCAGGTTTCTACCTTCGTGGTTTTACTTGAGTGACGCTGCTCATGCTTGCAA |
| CCGCTGGGATGCAGGTGCATGCCTCTAGCATGAAGTCAGACAA |
| >GS_C46 (*Cladocopium*) |
| AACCAATGGCCTCCTGAACGTGCGTTGCACTCTTGGGATTTCCTGAGAGTATGTCTGCTT |
| CAGTGCTTAACTTGCCCCAACTTTGCAAGCAGGATGTGTTTCTGCCTTGCGTTCTTATGA |
| GCTATTGCCCTCTGAGCCAATGGCTTGTGAATTGCTTGGTTCTTGCAAAATGCTATGCGC |
| GCTGTTATTCAGGTTTCTACCTTCGTGGTTTTACTTGAGTGACGCTGCTCATGCTTGCAA |
| CCGCTGGGATGCAGGTGCATGCCTCTAGCATGAAGTCAGACAA |
| >GS_C46a (*Cladocopium*) |
| AACCAATGGCCTCCTGAACGTGCGTTGCACTCTTGGGATTTCCTGAGAGTATGTCTGCTT |
| CAGTGCTTAACTTGCCCCAACTTTGCAAGCAGGACGTGTTTCTGCCTTGCGTTCTTATGA |
| GCTATTGCCCTCTGAGCCAATGGCTTGTGAATTGCTTGGTTCTTGCAAAATGCTATGCGC |
| GCTGTTATTCAGGTTTCTACCTTCGTGGTTTTACTTGAGTGACGCTGCTCATGCTTGCAA |
| CCGCTGGGATGCAGGTGCATGCCTCTAGCATGAAGTCAGACAA |
| >GS_C47 (*Cladocopium*) |
| AACCAATGGCCTCCTGAACGTGCGTTGCACTCTTGGGATTTCCTGAGAGTATGTCTGCTT |
| CAGTGCTTAACTTGCCCCAACTTTGCAAGCAGGATGTGTTTCTGCCTTGCGTTCTTATGA |
| GCTATTGCCCTCTGCGCCAATGGCTTGTGAATTGCTTGGTTCTTGCAAAATGCTTTGTGC |
| GCTGTTATTCAGGTTTCTACCTTCGTGGTTTTACTTGAGTGACGCTGCTCATGCTTGCAA |
| CCGCTGGGATGCAGGTGCATGCCTCTAGCATGAAGTCAGACAA |
| >GS_C48 (*Cladocopium*) |
| AACCAATGGCCTCCTGAACGTGCGTTGCACTCTTGGGATTTCCTGAGAGTATGTCTGCTT |
| CAGTGCTTAACTTGCCCCAACTTTGCAAGCAGGATGTGTTTCTGCCTTGCGTTCTTATGA |
| GCTATTGCCCTCTGAGCCAATGGCTTGTTAATTGCTTGGTTCTTGCAAAATGCTTTGCGC |
| GTGCTGTTATTCAAGTTTTTACCTTCGTGGTTTTACTTGAGTGACGCTGCTCATGCTTGC |
| AACCGCTGGGATGCAGGTGCATGCCTCTAGCATGAAGTCAGACAA |
| >GS_C49 (*Cladocopium*) |
| AACCAATGGCCTCCTGAACGTGCGTTGCACTCTTGGGATTTCCTGAGAGTATGTCTGCTT |
| CAGTGCTTAACTTGCCCCAACTTTGCAAGCAGGATGTGTTTCTGCCTTGCGTTCTTATGA |
| GCTATTGCCCTCTGAGCCAATGGCTTGTTAATTGCTTGGTTCTTGCAAAATGCTGAGCCA |
| ATGGCTTGTTAATTGCTTGGTTCTTGCAAAATGCTTTGCGCGCTGTTATTCAAGTTTCTA |
| CCTTCGTGGTTTTACTTGAGTGACGCTGCTCATGCTTGCAACCGCTGGGATGCAGGTGCA |
| TGCCTCTAGCATGAAGTCAGACAA |
| >GS_C50 (*Cladocopium*) |
| AACCAATGGCCTCCTGAATGTGCGTTGCACTCTTGGGATTTCCTGAGAGTATGTCTGCTT |
| CAGTGCTTAACTTGCCCCAACTTTGCAAGCAGGATGTGTTTCTGCCTTGCGTTCTTATGA |
| GCTATTGCCCTCTGAGCCAATGGCTTGTTAATTGCTTGGTTCTTGCAAAATGCTTTGCGC |
| GCTGTTATTCAAGTTTCTACCTTCGTGGTTTTACTTGAGTGACACTGCTCATGCTTGCAA |
| CCGCTGGGATGCAGGTGCATGCCTCTAGCATGAAGTCAGACAA |
| >GS_C51 (*Cladocopium*) |
| AAGCAATGGCCTCCTGAACGTGTGTTGCACTCTTGGGATTTCCTGAGAGTATGTCTGCTT |
| CAGTGCTTAACTTGCCCCAACTTTGCAAACAGGATGTGTTTCTGCCTTGCGTTCTTATGA |
| GCTATTGCCCTCTGAGCCAATGGCTTGTTAATTGCTTGGTTCTTGCAAAATGCTTTGCGC |
| AATGTTATTCAAGTTTCTACCTTCGTGGTTTTACTTGAGTGACGCTGCTCATGCTTGCAA |
| CCGCTGGGATGCAGGTGCATGCCTCTAGCATGAAGTCAGACAA |
| >GS_C52 (*Cladocopium*) |
| AGCCAATGGCCTCCTGAACGTGCGTTGCACTCTTGGGATTTCCTGAGAGTATGTCTGCTT |
| CAGTGCTTAACTTGCCCCAACTTTGCAAGCAGGATGTGTTTCTGCCTTGCGTTCTTACGA |
| GCTATTGCCCTCTGAGCCAATGGCTTGTTAATTGCTTGGTTCTTGCAAAATGCTTTGCGC |
| GCTGTTATTCAAGTTTCTACCTTCGTGGTTTACTTGAGTGACGCTACTCATGCTTGCAAC |
| CGCTGGGATGCAGGTGCATGCCTCTAGCATGAAGTCAGACAA |
| >GS_C53 (*Cladocopium*) |
| AACCAATGGCCTCCTGAACGTGCGTTGCACTCTTAGTATTTCCTGAGAGTATGTCTGCTT |
| CAGTGCTTACCTTGCCCCAACTTTGCAAGCAGGATGTGTTTCTGCCTTGCGTTCTTATGA |
| GCTATTGCCCTCTGAGCCAATGGCTTGTTAATTGCTTGGTTGTTGCAAAATGCTTTGCGC |
| GCTGTTACTCAAGTTTCTACCTTCGTGGGTTTACTGGAGTGACGCTGCTCATGCTTGCAA |
| CCGCTGGGATGCAGGTGCATGCCTCTAGCATGAAGTCAGACAA |
| >GS_C54a (*Cladocopium*) |
| AATCAATGGCCTCCTGAACGTGCGTTGCACTCTTGGGATTTCCTGAGAGTATGTCTGCTT |
| CAGTGCTTAACTTGCCCCAACTTTGCAAGCAGGATGTGTTTCTGCCTTGCGTTCTTATGA |
| GCTATTGCCCTCTGAGCCAATGGCTTGTTACTTGCTTGGTTCTTGCAAAATGCTTTGCGC |
| GCTGTTATTCAAGTTTCTACCTTCGTGGTTTTACTTGAGTGACACTGCTCATGCTTGCAA |
| CCGCTGGGATGCAGGTGCATGCCTCTAGCATGAAGTCAGACAA |
| >GS_C15=C55 (*Cladocopium*) |
| AACCAATGGCCTCCTGAACGTGCGTTGCACCCTTGGGATTTCCTGAGAGTATGTCTGCTT |
| CAGTGCTTAACTTGCCCCAACTTTGCAAGCAGGATGTGTTTCTGCCTTGCGTTCTTATGA |
| GCTATTGCCTTCTGCGCCAATGGCTTGTTAATTGCTTGGTTCTTGCAAAATGCTTTGCGC |
| GCTGTTATTCAAGTTTCTTTCTACCTTCGCGGTTTTACTTGAGTGACGCTGCTCATGCTT |
| GCAACCGCTGGGATGCAGGTGCATGCCTCTAGCATGAAGTCAGACAA |
| >GS_C56 (*Cladocopium*) |
| AACCAATGGCCTCCTGAACGTACGTTGCACCCTTGGGATTTCCTGAGAGTATGTCTGCTT |
| CAGTGCTTAACTTGCCCCAACTTTGCAAGCAGGATGTGTTTCTGCCTTGCGTTCTTATGA |
| GCTATTGCCTTCTGCGCCAATGGCTTGTTAATTGCTTGGTTCTTGCAAAATGCTTTGCGC |
| GCTGCTATTCACGTTTCTACCTTCGCGGTTTTACTTGAGTGACGCTGCTCATGCTTGCAA |
| CCGCTGGGATGCAGGTGCATGCCTCTAGCATGAAGTCAGACAA |
| >GS_C56a (*Cladocopium*) |
| AACCAATGGCCTCCTGAACGTGCGTTGCACCCTTGGGATTTCCTGAGAGTATGTCTGCTT |
| CAGTGCTTAACTTGCCCCAACTTTGCAAGCAGGATGTGTTTCTGCCTTGCGTTCTTATGA |
| GCTATTGCCTTCTGCGCCAATGGCTTGTTAATTGCTTGGTTCTTGCAAAATGCTTTGCGC |
| GCTGATATTCAAGTTTCTACCTTCGCGGTTCTACTTGAGTGACGCTGCTCATGCTTGCAA |
| CCGCTGGGATGCAGGTGCATGCCTCTAGCATGAAGTCAGACAA |
| >GS_C57a (*Cladocopium*) |
| AACCAATGGCCTCCTGAACGTGCGTTGCACTCTTGGGATTTCCTGAGAGTATGTCTGCTT |
| CAGTGCTTAACTTGCCCCAACTTTGCAAGCAGGATGTTTTTCTGCCTTGCGTTCTTATGA |
| GCTGTTGTCCTCTGTGCCAATGGCTTGTTAATTGCTTGGTGCTTGCAAAATGCTTTGCGC |
| GCTGTTATTCAAGTTTCTACCTTCGTGGTTTTACTTGAGTGACGCTGCTCATGCTTGCAA |
| CCCGCTGGGATGCAGGTGCATGCCTCTAGCATGAAGTCAGACAA |
| >GS_C57 (*Cladocopium*) |
| AACCAATGGCCTCCTGAACGTGCGTTGCACTCTTGGGATTTCCTGAGAGTATGTCTGCTT |
| CAGTGCTTAACTTGCCCCAACTTTGCAAGCAGGATGTTTTTCTGCCTTGCGTTCTTATGA |
| GCTATTGTCCTCTGTGCCAATGGCTTGTTAATTGCTTGGTGCTTGCAAAATGCTTTGCGC |
| GCTGTTATTCAAGTTTCTACCTTCGTGGTTTTACTTGAGTGACGCTGCTCATGCTTGCAA |
| CCCGCTGGGATGCAGGTGCATGCCTCTAGCATGAAGTCAGACAA |
| >GS_C58 (*Cladocopium*) |
| AACCAATGGCCTCCTGAACGTGCGTTGCACTCTTGGGATTTCCTGAGAGTATGTCTGCTT |
| CAGTGCTTAACTTGCCCCAACTTTGCAAGCATTTCTGCCTTGCGTTCTTATGAGCTATTG |
| CCCTCTGAGCCCAATGGCTTGTTAATTGCTTGGTTCTTGCAAAATGCTTTGCGCGCTGTT |
| ATTCAAGTTTCTACCTTCGTGGTTTTACTTGAGTGACGCTGCTCATGCTTGCAACCGCTG |
| GGATGCAGGTGCATGCCTCTAGCATGAAGTCAGACAA |
| >GS_C59 (*Cladocopium*) |
| AACCAATGGCCTCCTGAACGTGCGTTGCACTCTTGGGATTTCCTGAGAGTATGTCTGCTT |
| CAGTGCTTAACTTGCCCCAACTTTGCAAGCAGGATGTGTTTCTGCCTTTGCGCTCTTATG |
| AGCTATTGCCCTCTGAGCCAATGGCTTGTTAATTGCTTGGTTCTTGCAAACTGCTTTGCG |
| CGTTGTTATTCAGGTTTCTACCTTCGTGGTTTTACTTGAGTGACGGTGCTCATTGCTTGC |
| AACCGCTGGGATGCAGGTGCATGCCTCTAGCATGAAGTCAGACAA |
| >GS_C60 (*Cladocopium*) |
| AACCAATGGCCTCCTGAACGTGCGTTGCACCCTTGGGATTTCCTGAGAGTATGTCTGCTT |
| CAGTGCTTAACTTGCCCCAACTTTGCAAGCAGGATGTGTTTCTGCCTTGCGTTCTTATGA |
| GCTACTGCCTTCTGCGCCAATGGCTTGTTAATTGCTTGGTTCTTGCAAAATGCTTTGCGC |
| GCTGTTATTCAAGTTTCTACCTTCGCGGTTTTACTTGAGTGACGCTGCTCATGCTTGCAA |
| CCGCTGGGATGCAGGTGCATGCCTCTAGCATGAAGTCAGACAA |
| >GS_C61 (*Cladocopium*) |
| AACCAATGGCCTCCTGAACGTGCGTTGCACTCTTGGGATTTCCTGAGTGTATGTCTGCTT |
| CAATGCTTAACTTGCCCCAACTTTGCAAGCATTTTTCTGCCTTGCGTTTTTATGAGCTAT |
| TGCCCTCTGAGCCAATGGCTTGTTAATTGCTTGGTTCTTGCAAAATGCTTTGTGCGCTGT |
| TATTCAAGTTTCTACCGTCGTGGTTTTACTTGAGTGATGCTGCTCATGCTTGCAACCGCT |
| GGATGCAGGTGCATGCCTCTAGCATGAAGTCAGACAA |
| >GS_C62 (*Cladocopium*) |
| AACCAATCGCCTCCTGAACGTGCGTTGCACTCTTGGGATTTCCTGAGAGTATGTCTGCTT |
| CAGTGCTTAACTTGCCCCAACTTTGCAAGCAGGATGTGTTTCTGCCTTGCGTTCTTACGA |
| GCTATTGCCCTCTGAGCCAATGGCTTGTTAATTGCTTGGTTCTTGCAAAATGCTTTGCGC |
| GCTGTTATTCAAGTTTCTACCTTCGTGGTTTTACTTGAGTGACGCTGCTCATGCTTGCAA |
| CCGCTGGGATGCAGGTGCATGCCTCTAGCATGAAGTCAGACAA |
| >GS_C63a (*Cladocopium*) |
| AACCAATGGCCTCCTGAACGTGCGTTGCACTCTTGGGATTTCCTGAGAGTATGTCTGCTT |
| CAGTGCTTAACTTGCCCCAACTTTGCAAGCATTTCTGCCTTGCGTTCTTATGAGCTATTG |
| CCCTCTCTGAGCCAATGGCTTGTTAATTGCTTGGTTCTTGCGAAATGCTTTGCGCGCTGT |
| TATTCAAGGTTCTACCTTCGTGGTTTTACTTGAGTGACGCTGCTCATGCTTGCAACCGCT |
| GGGATGCAGGTGCATGCCTCTAGCATGAAGTCAGACAA |
| >GS_C64 (*Cladocopium*) |
| AACCAATGGCCTCCTGAACGTGCGTTGCACTCTTGGGATTTCCTGAGAGTATGTCTGCTT |
| CAGTGCTTAACTTGCCCCAACTTTGCAAGCAGGATGTGTTTCTGCCTTGTGTTCTTATGA |
| GTATTGCCCTCTGAGCCAATGGCTTGTTAATTGCTTGGTTCTTGCAAAATGCTTTGTGCG |
| CTGTTATTCAAGTTTCTACTTTCGTGGTTTTACTTGAGTGACGCTGCTCATGCTTGCAAC |
| CGCTGGGATGCAGGTGCATGCCTCTAGCATGAAGTCAGACAA |
| >GS_C65 (*Cladocopium*) |
| AACCAATGGCCTCCTGAACGTGCGTTGCACTCTTGGGATTTCCTGAGAGTATGTCTGCTT |
| CAGTGCTTAACTTGCCCCAACTTTGCAAGCAGGATGTGTTTCTGCCTTGCGTTCTTATGA |
| GCTATTGCCCTCTGAGCCAATGGCTTGTTAATTGCTTGGTTCTTGCAAAATGCTTTGCGC |
| GCTGTTATTCAAGTTTCTACCTTCGTCGTTTTACTTGAGTGACGCTGCTCATGCTTGCAA |
| CCGCTGGGATGCAGGTGCATGCCTCTAGCATGAAGTCAGACAA |
| >GS_C65a (*Cladocopium*) |
| AATCAATGGCCTCCTGAACGTGCGTTGCACTCTTGGGATTTCCTGAGAGTATGTCTGCTT |
| CAGTGCTTAACTTGCCCCAACTTTGCAAGCAGGATGTGTTTCTGCCTTGCGTTCTTATGA |
| GCTATTGCCCTCTGAGCCAATGGCTTGTTAATTGCTTGGTTCTTGCAAAATGCTTTGCGC |
| GCTGTTATTCAAGTTTCTACCTTCGTCGTTTTACTTGAGTGACGCTGCTCATGCTTGCAA |
| CCGCTGGGATGCAGGTGCATGCCTCTAGCATGAAGTCAGACAA |
| >GS_C66 (*Cladocopium*) |
| AACCAATGGCCTCCTGAACGTGCGTTGCACTCTTGGGATTTCCTGAGAGTATGTCTGCTT |
| CAGTGCTTAACTTGCCCCAACTTTGCAAGCAGGATGTTTTTCTGCCTTGCGTTCTTATGA |
| GCTATTGTCCTCTGCGCCAATGGCTTGTTAATTGCTTGGTGCTTGCAAAATGCTTTGCGC |
| GCTGTTATTCAAGTTTCTACCTTCGTGGTTTTACTTGAGTGACGCTGCTCATGCTTGCAA |
| CCCGCTGGGATGCAGGTGCATGCCTCTAGCATGAAGTCAGACAA |
| >GS_C66a (*Cladocopium*) |
| AACCAATGGCCTCCTGAACGTCTGCTTCAGTGCTTAACTTGCCCCAACTTTGCAAGCAGG |
| ATGTTTTTCTGCCTTGCGTTCTTATGAGCTATTGTCCTCTGCGCCAATGGCTTGTTAATT |
| GCTTGGTGCTTGCAAAATGCTTTGCGCGCTGTTATTCAAGTTTCTACCTTCGTGGTTTTA |
| CTTGAGTGACGCTGCTCATGCTTGCAACCCGCTGGGATGCAGGTGCATGCCTCTAGCATG |
| AAGTCAGACAA |
| >GS_C66b (*Cladocopium*) |
| AACCAATGGCCTCCTGAACGTGCGTTGCACTCTTGGGATTTCCTGAGAGTATGTCTGCTT |
| CAGTGCTTAGCTTGCCCCAACTTTGCAAGCAGGATGTTTTTCTGCCTTGCGTTCTTATGA |
| GCTATTGTCCTCTGCGCCAATGGCTTGTTAATTGCTTGGTGCTTGCAAAATGCTTTGCGC |
| GCTGTTATTCAAGTTTCTACCTTCGTGGTTTTACTTGAGTGACGCTGCTCATGCTTGCAA |
| CCCGCTGGGATGCAGGTGCATGCCTCTAGCATGAAGTCAGACAA |
| >GS_C67 (*Cladocopium*) |
| AACCAATGGCCTCCTGAACGTGCGTTGCACTCTTGGGATTTCCTGAGAGTATGTCTGCTT |
| CAGTGCTTAACTTGCCCCAACTTTGCAAGCAGGATGTGTTTCTGCCTTGCGTTCTTATGA |
| GCTATTGCCCTCCGAGCCAATGGCTTGTGAATTGCTTGGTTCTTGCAAAATGCTTTGCGC |
| GCTGTTATTCAGGTTTCTACCTTCGTGGTTTTACTTGAGTGACGCTGCTCATGCTTGCAA |
| CCGCTGGGATGCAGGTGCATGCCTCTAGCATGAAGTCAGACAA |
| >GS_C69 (*Cladocopium*) |
| AACCAATGGCCTCCTGAACGTGCGTTGCACTCTTGGGATTTCCTGAGAGAATGTCTGCTT |
| CAGTGCTTAACTTGCCCCAACTTTGCAAGCAGGATGTGTTTCTGCCTTGCGTTCTTATGA |
| GCTATTGCCCTCTGAGCCAATGGCTTGTTAATTCCTTGGTTCTTGCAAAATGCTTTGCGC |
| GCTGTTACTCAGATTTCTACCTGAGTGACGCTGCTCATGCTTGCAAACCGCTGGGATGCA |
| GGTGCATGCCTCTAGCATGAAGTCAGACAA |
| >GS_C69a (*Cladocopium*) |
| AATCAATGGCCTCCTGAACGTGCGTTGCACTCTTGGGATTTCCTGAGAGAATGTCTGCTT |
| CAGTGCTTAACTTGCCCCAACTTTGCAAGCAGGATGTGTTTCTGCCTTGCGTTCTTATGA |
| GCTATTGCCCTCTGAGCCAATGGCTTGTTAATTCCTTGGTTCTTGCAAAATGCTTTGCGC |
| GCTGTTACTCAGATTTCTACCTGAGTGACGCTGCTCATGCTTGCAAACCGCTGGGATGCA |
| GGTGCATGCCTCTAGCATGAAGTCAGACAA |
| >GS_C70 (*Cladocopium*) |
| AACCAATGGCCTCCTGAACGTGCGTTGCACTCTTGGGATTTCCTGAGAGTATGTCTGCTT |
| CAGTGCTTAACTTGCCCCAACTTTGCAAGCAGGATGTGTTTCTGCCTTGCGTTCTTACGA |
| GCTATTGCCCTCTGAGCCAATGGCTTGTTAATTGCTTGGTTCTTGCAAAATGCTTTGCGC |
| GCTGTTATTCAAGTTTCTACCTTCGTGGTTTTACTTGAGTGACGCTGCTCATGCTTGCAA |
| CCGCTGGGATGCAGGTGCATGCCTCTAGCATGAAGTCAGACAA |
| >GS_C71 (*Cladocopium*) |
| AACCAATGGCCTCCTGAACGTGCGTTGCACTCTTGGGATTTCCTGAGAGTATGTCTGCTT |
| CAGTGCTTAACTTGCCCCAACTTTGCAAGCAGGATGTGTTTCTGCCTTGCGTTCTTATGA |
| GCTATTGCCCTCTGAGCCAATGGCTTGTTAATTGCTTGGTTCTTGCAAAATGCTTTGCGC |
| GCTGTTATTCAAGTTTCTACCTTCGTCGTTTTACTTGAGTGACGCTGCTCGTGCTTGCAA |
| CCGCTGGGATGCAGGTGCATGCCTCTAGCATGAAGTCAGACAA |
| >GS_C71a (*Cladocopium*) |
| AGCCAATGGCCTCCTGAACGTGCGTTGCACTCTTGGGATTTCCTGAGAGTATGTCTGCTT |
| CAGTGCTTAACTTGCCCCAACTTTGCAAGCAGGATGTGTTTCTGCCTTGCGTTCTTATGA |
| GCTATTGCCCTCTGAGCCAATGGCTTGTTAATTGCTTGGTTCTTGCAAAATGCTTTGCGC |
| GCTGTTATTCAAGTTTCTACCTTCGTCGTTTTACTTGAGTGACGCTGCTCGTGCTTGCAA |
| CCGCTGGGATGCAGGTGCATGCCTCTAGCATGAAGTCAGACAA |
| >GS_C72 (*Cladocopium*) |
| AAGCAATGGCCTCCTGAACGTGCGTTGCACTCTTGGGATTTCCTGAGAGTATGTCTGCTT |
| CAGTGCTTAACTTGCCCCAACTTTGCAAGCAGGATGTGTTTCTGCCTTGCGTTCTTATGA |
| GCTATTGCCCTCCAATGGCTTGTTAATTGCTTGGTTCTTGCAAAATGCTTTGCGCGCTGT |
| TATTCAGGTTTCTACCTTCGTGGTTTTACTTGAGTGACGCTGCTCATGCTTGCAACCGCT |
| GGGATGCAGGTGCATGCCTCTAGCATGAAGTCAGACAA |
| >GS_C73 (*Cladocopium*) |
| AACCAGTGGCCTCCTGAACGTGCGTTGCACTCTTGGGATTTCCTGAGAGTATGTCTGCTT |
| CAGCGCTTAACTTGCCCCAACTTTGCAAGCATTTCTGCCTTGCGTTCTTATGAGCTATTG |
| CCCTCTGAGCCAATGGCTTGTTAATTGCTTGGTTCTTGCAAAATGCCTTGCGCGCACTGT |
| TATTCAAGCTTCTACCTTCGTGGTTTTACTTGAGTGATGACGCTGCTCATGCTTGCAACC |
| GCTGGGATGCAGGTGCATGCCTCTAGCATGAAGTCAGACAA |
| >GS_C74 (*Cladocopium*) |
| AACCAATGGCCTCCTGAACGTGCGTTGCACTCTTGGGATTTCCTGAGAGTATGTCTGCTT |
| TAGTGCTTAACTTGCCCCAACTTTGCAAGCAGGATGTTTTTCTGCCTTGCGTTCTTATGA |
| GCTATTGTCCTCTGCGCCAATGGCTTGTTAATTGCTTGGTGCTTGCAAAATGCTTTGCGC |
| GCTGTTATTCAAGTTTCTACCTTCGTGGTTTTACTTGAGTGATGCTGCTCATGCTTGCAA |
| CCCGCTGGGATGCAGGTGCATGCCTCTAGCATGAAGTCAGACAA |
| >GS_C75 (*Cladocopium*) |
| AACCAATGGCCTCCTGAACGTGCGTTGCACTCTTGGGATTTCCTGAGAGTATGTCTGCTT |
| CAGTGCTTAACTTGCCCCAACTTTGCAAGCAGGATGTGTTTCTGCCTTGCGGTTTTATGA |
| GCTATTGCCCTCTGAGCCAATGGCTTGTTAATTGCTTGGTTCTTGCAAAATGCTTTGCGC |
| GCTGTTATTCAAGTTTCTACCTTCGTGGTTTTACTTGAGTGACGCTGCTCATGCTTGCAA |
| CCGCTGGGATGCAGGTGCATGCCTCTAGCATGAAGTCAGACAA |
| >GS_C76 (*Cladocopium*) |
| AATCAATGGCCTCCTGAACGTGCATTGCACTCTTGGGATTTCCTGAGAGTATGTCTGCTT |
| CAGTGCTTAACTTGCCCCAACTTTGCAAGCAGGATGTGTTTCTGCCTTGCGTTCTTATGA |
| GCTATTGCCCTCTGAGCTAATGGCTTGTTAATTGCTTGGTCTTGCAAAATGCTTTGCGCG |
| CTTTATTCAAGTTTCTACCTTCGTGGTTTTACTTGAGTGACGCTGCTCATGCTTGCAACC |
| GCTGGGATGCAGGTGCATGCCTCTAGCATGAAGTCAGACAA |
| >GS_C77 (*Cladocopium*) |
| AAGCAATGGCCTCCTGAACGTGCGTTGCACTCTTGGGATTTCCTGAGAGTATGTCTGCTT |
| CAGTGCTTAACTTGCCCCAACTTTGCAAGCAGGATGTGTTTCTGCCTTGCGTTCTTATGA |
| GCTATTGCCCTCTGAGCTAATGGCTTGTTAATTGCTTGGTCTTGCAAAATGCTTTGCGCG |
| CGCTGTCATTCAAGTTTCTACCTTCGTGGTTTTACTTGAGTGACGCTGCTCATGCTTGCA |
| ACCGCTGGGATGCAGGTGCATGCCTCTAGCATGAAGTCAGACAA |
| >GS_C78 (*Cladocopium*) |
| AATCAATGGCCTCCTGAACGCGCGTTGCAGTCTTGGGATTTCCTGAGAGTATGTCTGCTT |
| CAGTGCTTAACTTGCCCCAACTTTGCAAGCAGGATGTGTTTCTGTCTTGCGTTCGTATGA |
| GCTATTGCCCTCTGAGCCAGTGGCTTGTTAATTGCTTGGTTCTTGCAACATGCTTTGCGC |
| GCTGTTATTCAGGTTTCTACCTTCGCGGTTTTACTTGAGTGACACTGCTCATGCTTGCAA |
| CCGCTGGGATGGAGGTGCATGCCTCTAGCATGAAGTCAGACAA |
| >GS_C78a (*Cladocopium*) |
| AACCAATGGCCTCCTGAACGTGCGTTGCACTCTTGGGATTTCCTGAGAGTATGTCTGCTT |
| CAGTGCTTAACTTGCCCCAACTTTGCAAGCAGGATGTGTTTCTGCCTTGCGTTCTTATGA |
| GCTATTGCCCTCTGAGCCAATGGCTTGTTAATTGCTTGGTTCTTGCAAAATGCTTTGCGC |
| GCTGTTATTCAGGTTTCTACCTTCGTGGTTTTACTTGAGTGACGCTGCTCATGCTTGCNA |
| CCGCTGGGATGCAGGTGCATGCCTCTAGCATGAAGTCAGACAA |
| >GS_C79 (*Cladocopium*) |
| AACCAATGGCCTCCTGAACGTGCGTGGCACTCTTGGGATTTCCTGAGAGTATGTCTGCTT |
| CAGTGCTTAACTTGCCCCAACTTTGCAAGCAGGATGTGTTTCTGCCTTGCGTTCTTATGA |
| GCTATTGGCCTCTGAGCCAATGGCTTGTTAATTACTTGGTTCTTGCAAAATGCTTTGCGC |
| GCTGTTATTCAAGTTTCTACCTTCGTGGTTTTACTTGAGTGACACTGCTCATGCTTGCGA |
| CCGCTGGGATGCAGGTGCATGCCTCTAGCATGAAGTCGGACAA |
| >GS_C1a=C80 (*Cladocopium*) |
| AACCAATGGCCTCCTGAACGTGCGTTGCACTCTTGGGATTTCCTGAGAGTATGTCTGCTT |
| CAGTGCTTAACTTGCCCCAACTTTGCAAGCAGGATGTGTTTCTGCCTTGCGTTCTTATGA |
| GCTATTGCCCTCTGAGCCAATGGCTTGTTAATTGCTTGGTTCTTGCAAAATGCTTTGCGC |
| GCTGTTATTCAGGTTTCTACCTTCGCGGTTTTACTTGAGTGACGCTGCTCATGCTTGCAA |
| CCGCTTGCAACCGCTGGGATGCAGGTGCATGCCTCTAGCATGAAGTCAGACAA |
| >GS_C81 (*Cladocopium*) |
| AACCAATGGCCTCCTGAACGTGCGTTGCACTCTTGGGATTTCTTTCCTGAGAGTATGTCT |
| GCTTCAGTGCTTAACTTGCCCCAAATTTGCAAGCAGGATGTGTTTCTGCCTTGTGTTCTT |
| ATGAGCTATTGCCCTCTGAGCCAATGGCTTGTTAATTGCTTGGTTCTTGCAAAATGCTTT |
| GCGCGCTGTTATTCAAGTTTCTACCGCTGCTCATGCTTGCAACCGCTGGGATGCAGGTGC |
| ATGCTTCTAGCATGAAGTCAGACAA |
| >GS_C82=C82b (*Cladocopium*) |
| AACCAATGGCCTCCTGAACGTGCGTTGCACTCTTGGGATTTCCTGAGAGTATGTCTGCTT |
| CAGTGCTTAACTTGCCTCAATTTTGCAAGCAGGATGTGTTTCTGCCTTGCGTTCTTATGA |
| GCCATTGCCCTCTGAGCCAATGGCTTGTGAATTGCTTGGTTCTTGCAAAATGCTTTGCGC |
| GCTGTTATTCAGGTTTCTACCTTCGTGGTTTTACTTGAGTGACGCTGCTCATGCTTGCAA |
| CCGCTGGGATGCAGGTGCATGCCTCTAGCATGAAGTCAGACAA |
| >GS_C3=C83=Cspc (*Cladocopium*) |
| AACCAATGGCCTCCTGAACGTGCGTTGCACTCTTGGGATTTCCTGAGAGTATGTCTGCTT |
| CAGTGCTTAACTTGCCCCAACTTTGCAAGCAGGATGTGTTTCTGCCTTGCGTTCTTATGA |
| GCTATTGCCCTCTGAGCCAATGGCTTGTTAATTGCTTGGTTCTTGCAAAATGCTTTGCGC |
| GCGCTGTTATTCAAGTTTCTACCTTCGTGGTTTTACTTGAGTGACGCTGCTCATGCTTGC |
| AACCGCTGGGATGCAGGTGCATGCCTCTAGCATGAAGTCAGACAA |
| >LJ_C84 (*Cladocopium*) |
| TTGTCTGACTTCATGCTAGAGGCATGCACCTGCATCCCAGCGGTTGCAAGCATGAGCAGCGTCACTCAACTAAAACCACGAAGGTAGAAACTTGAATAACAGCGCGCAGCATTTTGCAAGAACCAAGCAATTAACAAGCCATTGCCTCAGAGGGCAATAGTTCATAAGAAAACGCAAGGCAGAAACACATCCTGCTTGCAAAGTTGGGGCAAGTTAAGCACTGAAGCAGACATACTCTCAGGAAATCCCAAGAGTGCAACGCACATTCAGGAGGCCACTGGTT |
| >LJ_C84a (*Cladocopium*) |
| TTGTCTGACTTCATGCTAGAGGCATGCACCTGCATCCCAGCGGTTGCAAGCATGAGCAGCGTCACTCAACTAAAACCACGAAGGTAGAAACTTGAATAACAGCGCGCAGCATTTTGCAAGAACCAAGCAATTAACAAGCCATTGCCTCAGAGGGCAATAGTTCATAAGAAAATGCAAGGCAGAAACACATCCTGCTTGCAAAGTTGGGGCAAGTTAAGCACTGAAGCAGACATACTCTCAGGAAATCCCAAGAGTGCAACGCACATTCAGGAGGCCACTGGTT |
| >LJ_C86 (*Cladocopium*) |
| TTGTCTGACTTCATGCTAGAGGCATGCACCTGCATCCCAGCGGTTGCAAGCATGAGCAGCGTCACTCAAGTAAAACCACGAAGGTAGAAACCTGAATAACAGCGGTCAAAGCATTTTGCAAGAACCAAGCAATTCACAAGCCATTGGCTCAGAGGGCAATAGCTCATAAGAACGCAAGGCAGAAACACATCCTGCTTGCAAAGTTGGGGCAAGTTAAGCACTGAAGCAGACATACTCTCAGGAAATCCCAAGAGTGCAACGCACGTTCAGGAGGCCATTGGTT |
| >LJ_C87 (*Cladocopium*) |
| TTGTCTGACTTCATGCTAGAGGCATGCACCTGCATCCCAGCGGTTGCAAGCATGAGCAGCGTCACTCAAGTAAAACCACGAAGGTAGAAACTTGAATAACAGCGCGCAAAGCATTTTGCAAGAACCAAGCAATTAACAAGCCATTGGCTCAGAGGGCAATAAGCTCATAAGAACGCAAGGCAGAAACACATCCTGCTTGCAAAGTTGGGGCAAATTAAGCACTGAAGCAGACATACTCTCAGGAAATCCCAAGAGTGCAACGCACGTTCAGGAGGCCATTGGTT |
| >GS_C88a (*Cladocopium*) |
| AATCAATGGCCTCCTGAACGTGCGTTGCACTCTTGGGATTTCCTGAGAGTATGCCTGCTT |
| CAGCGCTTAACTTGCCCCAACTTTGCAAGCATTCTCCTTGCGTTCCTATGAGCTATTGCC |
| CTCTGAGCCAATGGCTTGTTAATTGCTTGGTTCTTGCAAAATGCTTTGCGTGCTGTTATT |
| CAAGTTTCTACCTTCGTGGTTTTACTTGAGTGACGCTGCTCATGCTTGCAACCGCTGGGA |
| TGCAGGTGCATGCCTCTAGCATGAAGTCAGACAA |
| >GS_C89 (*Cladocopium*) |
| AACCAATGGCCTCCTGAACGTGCGTTGCACTCTTGGGATTTCCTGAGAGTATGTCTGCTT |
| CAGTGCTTAACTTGCCCCAACTTTGCAAGCAGGATGTGTTTCTGCCTTGCGTTCTTATGA |
| GCTATTGCCCTCTGAGCCAATGGCTTGTTAATTGCTTGGTTCTTGCAAAATGCTTTGCGC |
| GCTGTTATTCAGGTTTCTACCTTCGTGGTTTTACTTGAGTGATGCTGCTCATGCTTGCAA |
| CCGCTGGGATGCAGGGTGCATGCCTCTAGCATGAAGTCAGACAA |
| >GS_C90 (*Cladocopium*) |
| GCTTCAGTGCTTAACTTGCCCCAACTTTGCAAGCAGGATGTGTTTCTGCCTTGCGTTCTT |
| ATGAGCTATTGCCCTCTGCGCCAATGGCTTGTTAATTGCTTGGTTCTTGCAAAATGCTTT |
| GCGCGCTGTTATTCAAGTTTCTACCTTCAAGTGGTTTTACTTGAGTGACGCTGCTCATGC |
| TTGCAACCGCTGGGATGCAGGTGCATGCCTCTAGCATGAAGTCAGACAA |
| >GS_C91 (*Cladocopium*) |
| AACCAATGGCCTCCTGAACGTGCGTTGCACTCTTGGGATTTCCTGAGAGTATGTCTGCTT |
| CAGTGCTTAACTTGCCCCAACTTTGCAAGCAGGATGTGTTTCTGCCTTGCGTTCTTATGA |
| GCTATTGCCCTCTGCGCCAATGGCTTGTTAATTGCTTGGTTCTTGCAAAATGCTTTGCGC |
| GCTGTTATTCAAGTTTCTACCTTCGCGGTTTTACTTGAGTGACGCTGCTCATGCTTGCAA |
| CCGCTGGGATGCAGGTGCATGCCTCTAGCATGAAGTCAGACAA |
| >GS_C91a (*Cladocopium*) |
| AACCAATGGCCTCCTGAACGTGCGTTGCACTCTTGGGATTTCCTGAGAGTATGTCTGCTT |
| CAGTGCTTAACTTGCCCCAACTTTGCAAGCAGGATGTGTTTCTGCCTTGCGTTCTTATGA |
| GCTATTGCCCTCTGCGCCAATGGCTTGTTAATTGCTTGGTTCTTGCAAAATGCTTTGCGC |
| GCTGTTATTCAAGTTTCTACCTTCGCGGCTTTACTTGAGTGACGCTGCTCATGCTTGCAA |
| CCGCTGGGATGCAGGTGCATGCCTCTAGCATGAAGTCAGACAA |
| >GS_C91b (*Cladocopium*) |
| AACCAATGGCCTCCTGAACGTGCGTTGCACTCTTGGGATTTCCTGAGAGTATGTCTGCTT |
| CAGTGCTTAACTTGCCCCAACTTTGCAAGCAGGATGTGTTTCTGCCTTGCGTTCTTATGA |
| GCTATTGCCCTCTGCGCCAATGGCTTGTTAATTGCTTGGTTCTTGCAAAATGCTTTGCGC |
| GCTGTTATTCAAGTTTCTACCTTCGCGATTTTACTTGAGTGACGCTGCTCATGCTTGCAA |
| CCGCTGGGATGCAGGTGCATGCCTCTAGCATGAAGTCAGACAA |
| >GS_C91c (*Cladocopium*) |
| AACCAATGGCCTCCTGAACGTGCGTTGCACTCTTGGGATTTCCTGAGAGTATGTCTGCTT |
| CAGTGCTTAACTTGCCCCAACTTTGCAAGCAGGATGTGTTTCTGCCTTGCGTTCTTATGA |
| GCTATTGCCCTCTGCGCCAATGGCTTGTTAATTGCTTGGTTCTTGCAAAATGCTTTGCGC |
| GCTGTTATTCGAGTTTCTACCTTCGCGGTTTTACTTGAGTGACGCTGCTCATGCTTGCAA |
| CCGCTGGGATGCAGGTGCATGCCTCTAGCATGAAGTCAGACAA |
| >GS_C91d (*Cladocopium*) |
| AACCAATGGCCTCCTGAACGTGCGTTGCACTCTTGGGATTTCCTGAGAGTATGTCTGCTT |
| CAGTGCTTAACTTGCCCCAACTTTGCAAGCAGGATGTGTTTCTGCCTTGCGTTCTTATGA |
| GCTATTGCCCTCTGCGCCAATGGCTTGTTAATTGCTTGGTTCTTGCAAAATGCTTTGCGC |
| GCTGTTATTCTAGTTTCTACCTTCGCGGTTTTACTTGAGTGACGCTGCTCATGCTTGCAA |
| CCGCTGGGATGCAGGTGCATGCCTCTAGCATGAAGTCAGACAA |
| >GS_C92 (*Cladocopium*) |
| AACCAATGGCCTCCTGAACGTGCGTTGCACTCTTGGGATTTCCTGAGAGTATGTCTGCTT |
| CAGTGCTTAACTTGCCCCAACTTTGCAAGCAGGATGTGTTTCTGCCTTGCGTTCTTATGA |
| GCTATTGCCCTCTGCGCCAATGGCTTGTTAATTGCTCGGTTCTTGCAAAATGCTTTGCGC |
| GCTGTTATTCAAGTTTCTACCTTCGTGGTTTTGCTTGAGTGACGCTGCTCATGCTTGCAA |
| CCGCTGGGATGCAGGTGCATGCCTCTAGCATGAAGTCAGACAA |
| >GS_C92a (*Cladocopium*) |
| AACCAATGGCCTCCTGAACGTGCGTTGCACTCTTGGGATTTCCTGAGAGTATGTCTGCTT |
| CAGTGCTTAACTTGCCCCAACTTTGCAAGCAGGATGTGTTTCTGCCTTGCGTTCTTATGA |
| GCTATTGCCCTCTGCGCCAATGGCTTGTTAATTGCTCGGTTCTTGCAAAATGCTTTGCGC |
| GCTGTTATTCAAGTTTCTACCTTCGTGGTTTTGCTTGAGTGACGCTGCTCATGCTTGCAA |
| CTGCTGGGATGCAGGTGCATGCCTCTAGCATGAAGTCAGACAA |
| >GS_C93_(type_1) (*Cladocopium*) |
| AACCAATGGCCTCCTGAACGTGCGTTGCACCCTTGGGATTTCCTGAGAGTATGTCTGCTT |
| CAGTGCTTAACTTGCCCCAACTTTGCAAGCAGGATGTGTTTCTGCCTTGCGTTCTTATGA |
| GCTATTGCCCTCTGAGCCAATGGCTTGTTAATTGCTTGGTTCTTGCAAAATGCTTTGCGC |
| GCTGTTATTCAAGTTTCTACCTTCGTGGTTTTACTTGAGTGACGCTGCTCATGCTTGCAA |
| CCGCTGGGATGCAGGTGCATGCCTCTAGCATGAAGTCAGACAA |
| >GS_C93_(type_2) (*Cladocopium*) |
| AACCAATGGCCTCCTGAACGTGCGTTGCACTCTTGGGATTTCCTGAGAGTATGTCTGCTT |
| CAGTGCTTAACTTGCCCCAACTTTGCAAGCAGGATGTGTTTCTGCCTTGCGTTCTTATGA |
| GCTATTGCCTTTTGCGCCAATGGCTTGTTAATTGCTTGGTTCTTGCAAAATGCTTTGCGC |
| GCTGTTATTCAAGTTTCTACCTTCGCGGTTTTACTTGAGTGACGCTGCTCATGCTTGCAA |
| CCGCTGGGATGCAGGTGCATGCCTCTAGCATGAAGTCAGACCA |
| >GS_C93a (*Cladocopium*) |
| AACCAATGGCCTCCTGAACGTGCGTTGCACTCTTGGGATTTCCTGAGAGTATGTCTGCTT |
| CAGTGCTTAACTTGCCCCAACTTTGCAAGCAGGATGTGTTTCTGCCTTGCGTTCTTATGA |
| GCTATTGCCTTTTGCGCCAATGGCTTGTTAATTGCTTGGTTCTTGCAAAATGCTTTGCGC |
| GCTGTTATTCAAGTTTCTACCTTCGCGGTTTTACTTGAGTGACGCTGCTCATGCTTGCAA |
| CCGCTGGGATGCAGGTGCATGCCTCTAGCATGAAGTCAGACAA |
| >GS_C94 (*Cladocopium*) |
| AACCAATGGCCTCCTGAACGTGCGTTGCACTCTTGGGATTTCCTGAGAGTATGTCTGCTT |
| CAGTGCTTAACTTGCCCCAACTTTGCAAGCAGGATGTGTTTCTGCCTTGCGTTCTTATGA |
| GCCATTGCCCTCTGAGCCAATGGCGTGTTAATTGCTTGGTTCTTGCAAAATGCTTTGCGC |
| GCTGTTATTCAAGTTTCTACCTTCGTGGTTTTACTTGAGTGACGCTGCTCATGCTTGCAA |
| CCGCTGGGATGCAGGTGCATGCCTCTAGCATGAAGTCAGACAA |
| >GS_C94a (*Cladocopium*) |
| AACCAATGGCCTCCTGAACGTGCGTTGCACTCTTGGGATTTCCTGAGAGTATGTCTGCTT |
| CAGTGCTTAACTTGCTCCAACTTTGCAAGCAGGATGTGTTTCTGCCTTGCGTTCTTATGA |
| GCCATTGCCCTCTGAGCCAATGGCGTGTTAATTGCTTGGTTCTTGCAAAATGCTTTGCGC |
| GCTGTTATTCAAGTTTCTACCTTCGTGGTTTTACTTGAGTGACGCTGCTCATGCTTGCAA |
| CCGCTGGGATGCAGGTGCATGCCTCTAGCATGAAGTCAGACAA |
| >GS_C96 (*Cladocopium*) |
| AACCAATGGCCTCCTGAACGTGCGTTGCACTCTTGGGATTTCCTGAGAGTATGTCTGCTT |
| CAGTGCTTAACTTGCCCCGACTTTGCAAGCACTCTTCTGCCTTGCGTTCTTATGAGCTAT |
| TGCCCTCTCTGAGCCAATGGCTTGTTAATTGCTTGGTTCTTGCAAAATGCTTTGCGCGCT |
| GTTATTCAAGTTTCTACGTTCGTGGTTTTACTTGAGTGATGCTGCTCATGCTTGCAACCG |
| CTGGGATGCAGGTGCATGCCTCTAGCATGAAGTCAGACAA |
| >GS_C101 (*Cladocopium*) |
| AACCAATGGCCTCCTGAACGTGCGTTGCACTCTTGGGATTTCCTGAGAGTATGTCTGCTT |
| CAGTGCTTAACTTGCCCCAACTTTGCAAGCAGGATGTGTTTCTGCCTTGCGTTCTTATGA |
| GCTATTGCCCTCTGAGCCAATGGCTTGTTAATTGCTTGGTTCTTGCAAAATGCTTTGCGC |
| GCTGTTATTCAAGTTTCTACCTTCGTGGTTTTACTTGAGTGATGCTGCTCATGCTTGCAA |
| CCGCTGGGATGCAGGTGCATGCCTCTAGCATGAACTCAGACAA |
| >GS_C105 (*Cladocopium*) |
| AACCAATGGCCTCCTGAACGTGCGTTGCACTCTTGGGATTTCCTGAGAGTATGTCTGCTT |
| CAGTGCTTCACTTGCCCCAACTTTGCAAGCAGGATGTGTTTCTGCCTTGCGTTCTTATGA |
| GCTATTGCCCTCCAATGGCTTGTTAATTGCTTGGTTCTTGCAAAATGCTTTGCGCGCTGT |
| TAATTCAGGTTTCTACCTTCGTGGTTTTACTTGAGTGACGCGGCTCATGCTTGCAACCGC |
| TGGGATGCAGGTGCATGCCTCTAGCATGAAGTCAGACAA |
| >GS_C105a (*Cladocopium*) |
| AACCAATCGCCTCCTGAACGTGCGTTGCACTCTTGGGATTTCCTGAGAGTATGTCTGCTT |
| CAGTGCTTCACTTGCCCCAACTTTGCAAGCAGGATGTGTTTCTGCCTTGCGTTCTTATGA |
| GCTATTGCCCTCCAATGGCTTGTTAATTGCTTGGTTCTTGCAAAATGCTCCTCGCTGTTA |
| ATTCAGGTTTCTACCTTCGTGGTTTTACTTGAGTGACGCGGCTCATGCTTGCAACCGCTG |
| GGATGCAGGTGCATGCCTCTAGCATGAAGTCAGACAA |
| >GS_C106 (*Cladocopium*) |
| AACCAATGGCCTCCTGAACGTGCGTTGCACTCTTGGGATTTCCTGAGAGTATGTCTGCTT |
| CAGTGCTTAACTTGCCCCAACTTTTGCAAGCAGGATGTGTTTCTGCCTTGCGTTCTTATG |
| AGCTATTGCCCTCTGAGCCAATGGCTTGTTAATTGCTTGGTTCTTGCAAAATGCTTTTGC |
| GCGCTGTTATTCAGGTTTCTACCTTCGTGGTTTTACTCGAGTGATGCTGCTCGTGCTTGC |
| AACCACTGGGATGCAGGTGCATGCCTCTAGCATGAAGTCAGACAA |
| >GS_C106a (*Cladocopium*) |
| AACCAATGGCCTCCTGAACGTGCGTTGCACTCTTGGGATTTCCTGAGAGTATGTCTGCTT |
| CAGTGCTTAACTTGCCCCAACTTTGCAAGCAGGATGTGTTTCTGCCTTGCGTTCTTATGA |
| GCTATTGTCCTCTGAGCCAATGGCTTGTTAATTGCTTGGTTCTTGCAAAATGCTTTTGCG |
| CGCTGTTATTCAGGTTTCTACCTTCGTGGTTTTACTTGAGTGATGCTGCTCGTGCTTGCA |
| ACCACTGGGATGCAGGTGCATGCCTCTAGCATGAAGTCAGACAA |
| >GS_C107 (*Cladocopium*) |
| AAGCAATGGCCTCCTGAACGTGCGTTGCACTCTTGGGATTTCCTGAGAGTATGTCTGCTT |
| CAGTGTTTAACTTGCCCCAACTTTGCAAGCAGGATGTGTTTCTGCCTTGCGTTCTTATGA |
| GCTATTGCCCTCTGAGCCAATGGCTTGTTAATTGCTTGGTTCTTGCAACATGCTTTGCGC |
| GCGCTGTTATTCAAGTTTCTACCTTCGTGGTTTTACTTGAGTGACGCTGCTCATGCTTGC |
| AACCGCTGGGATGCAGGTGCATGCCTCTAGCATGAAGTCAGACAA |
| >GS_C107a (*Cladocopium*) |
| AACCAATGGCCTCCTGAACGTGCGTTGCACTCTTGGGATTTCCTGAGAGTATGTCTGCTT |
| CAGTGTTTAACTTGCCCCAACTTTGCAAGCAGGATGTGTTTGTGCCTTGCGTTCTTATGA |
| GCTATTGCCCTGTGAGCCAATGGCTTGTTAATTGCTTGGTTCTTGCAAAATGCTTTGCGC |
| GCTGTTATTCAAGTTTCTACCTTCGTGGTTTTACTTGAGTGACGCTGCTCATGCTTGCAA |
| CCGCTGGGATGCAGGTGCATGCCTCTAGCATGAAGTCAGACAA |
| >GS_C107b (*Cladocopium*) |
| AACCAATGGCCTCCTGAACGTGCGTTGCACTCTTGGGATTTCCTGAGAGTATGTCTGCTT |
| CAGTGTTTAACTTGCCCCAACTTTGCAAGCAGGATGTGTTTGTGCCTTGCGTTCTTATGA |
| GCTATTGCCCTCTGAGCCAATGGCTTGTTAATTGCTTGGTTCTTGCAACATGCTTTGCGC |
| GCGCGCTGTTATTCAAGTTTCTACCTTCGTGGTTTTACTTGAGTGACGCTGCTCATGCTT |
| GCAACCGCTGGGATGCAGGTGCATGCCTCTAGCATGAAGTCAGACAA |
| >GS_C110 (*Cladocopium*) |
| AACCAATGGCCTCCTGAACGTGCGTTGCACTCTTGGGATTTCCTGAGAGTATGCCTGCTT |
| CAGCGCTTAACTTGCCCCAACTTTGCAAGCATTCTCCTTGCGTTCCTATGAGCTATTGCC |
| CTCTGAGCCAATGGCTTGTTAATTGCTTGGTTCTTGCAAAATGCTTTGCGTGCTGTTATT |
| CAAGTTTCTACCTTCGTGGTTTTACTTGAGTGACGCTGCTCATGCTTGCAACCGCTGGGA |
| TGCAGGTGCATGCCTCTAGCATGAAGTCAGACAA |
| >GS_C112 (*Cladocopium*) |
| AACCAATGGCCTCCTGAACGTGCGTTGCACTCTTGGGATTTCCTGAGAGTAGGTCTGCTT |
| CAGTGCTTAACTTGCCCCAACTTTGCAAGCAGGATGTGTTTCTGCCTTGCGTTCTTATGA |
| GCTATTGCCCTCTGAGCCAATGGCTTGTGAATTGCTTGGTTCTTGCAAAATGCTTTGCGC |
| GCTGTTATTCAGGTTTCTACCTTCGTGGTTTTACTTGAGTGACGCTGCTCGTGCTTGCAA |
| CCGCTGGGATGCAGGTGCATGCCTCTAGCATGAAGTCAGACAA |
| >GS_C113=C114 (*Cladocopium*) |
| AACCAATGGCCTCCTGAACGTGCGTTGCACTCTTGGGATTTCCTGAGAGTATGTCTGATT |
| CAGTGCTTAACTTACCCCAACTTTGCAAGCAGGATGTGTTTCTGCCTTGCGTTCTTATGA |
| GCTATTGCCCTCTGAGCCAATGGCTTGTTACTTGCTTGGTTCTTGCAAAATGCTTTGCGC |
| GCTGTTACTCAGGTTTCTACCTTCGTGGTTTTACTTGAGTGACGCTGCTCATGCTTGCAA |
| CCGCTGGGATGCAGGTGCATGCCTCTAGCATGAAGTCAGACAA |
| >GS_C115 (*Cladocopium*) |
| AACCAATGGCCTCCTGAACGTGCGTTGCACTCTTGGGATTTCCTGAGAGTATGTCTGCTT |
| CAGTGCTTAACTTGCCCCAACTTTGCAAGCAGGATGTGTTTCTGCCTTGCGTTCTTATGA |
| GCTATTGCCCTCTGAGCCAATGGCTTGTTAATTGCTTGGTTCTTGCAAAATGCTTTGCGC |
| GCTGTTATTCAAGTTTCTACCTTCGTGGTTTTACTTGACTGACGCTGCTCATGCTTGCAA |
| CCGCTGGGATGCAGGTGCATGCCTCTAGCATGAAGTCAGACAA |
| >GS_C115a (*Cladocopium*) |
| AACCAATGGCCTCCTGAACGTGCGTTGCACTCTTGGGATTTCCTGAGAGTATGTCTGCTT |
| CAGTGCTTAACTTGCCCCAACTTTGCAAGCAGGATGTGTTTCTGCCTTGCGTTCTTATGA |
| GCTATTGCCCTCTGAGCCAATGGCTTGTTAATTGCTTGGTTCTTGCAAAATGCTTTGCGC |
| GCTGTTATTCAAGTTTCTACCTTTGTGGTTTTACTTGACTGACGCTGCTCATGCTTGCAA |
| CCGCTGGGATGCAGGTGCATGCCTCTAGCATGAAGTCAGACAA |
| >GS_C116 (*Cladocopium*) |
| AACCAATGGCCTCCTGAACGTGCGTTGCACCCTTGGGATTTCCTGAGAGTATGTCTGCTT |
| CAGTGCTTAACTTGCCCCAACTTTGCAAGCAGGATGTGTTTCTGCCTTGCGTTCTTATGA |
| GCTATTGCCTTCTGCGCCAATGGCTTGTTAATTGCTTGGTTCTTGCAAAATGCTTTGCGC |
| GCTGTTATTCAAGTTTCTACCTTCCCGGTTTTACTTGAGTGACGCTGCTCATGCTTGCAA |
| CCGCTGGGATGCAGGTGCATGCCTCTAGCATGAAGTCAGACAA |
| >GS_C119 (*Cladocopium*) |
| AAGCAATGGCCTCCTGAACGTGCGTTGCACTCTTGGGATTTCCTGAGAGTATGTCTGCTT |
| CAGTGCTTAACTTGCCCCAACTTTGCAAGCAGGATGTGTTTCTGCCTTGCGTTCTTATGA |
| GCTATTGCCCTCTGAGCCAATGGCTTGTTAATTGCTTGGTTCTTGCAAAATGCTTTGCGC |
| GCTGTTATTCAAGTTTCTACCTTCGCGATTTTACTTGAGTGACGCTGCTCATGCTTGCAA |
| CCGCTGGGATGCAGGTGCATGCCTCTAGCATGAAGTCAGACAA |
| >GS_C120 (*Cladocopium*) |
| AACCAATGGCCTCCTGAACGTGCGTTGCACTCTTGGGATTTCCTGAGAGTATGTCTGCTT |
| CAGTGCTTAACTTGCCCCAACTTTGCAAGCAGGATGTGTTTCTGCCTTGCGTGCTTATGA |
| GCTATTGCCCTCTGAGCCAATGGTTTGTGAATTGCTTGGTTCTTGCAAAATGCTTTGCGC |
| GCTGTTATTCAGGTTTCTACCTTCGTGGTTTTACTTGAGTGACGCTGCTCATGCTTGCAA |
| CCGCTGGGATGCAGGTGCATGCCTCTAGCATGAAGTCAGACAA |
| >GS_C120a (*Cladocopium*) |
| AACCAATGGCGTCCTGAACGTGCGTTGCACTCTTGGGATTTCCTGAGAGTATGTCTGCTT |
| CAGTGCTTAACTTGCCCCAACTTTGCAAGCAGGATGTGTTTCTGCCTTGCGTGCTTATGA |
| GCTATTGCCCTCTGAGCCAATGGTTTGTGAATTGCTTGGTTCTTGCAAAATGCTTTGCGC |
| GCTGTTATTCAGGTTTCTACCTTCGTGGTTTTACTTGAGTGACGCTGCTCATGCTTGCAA |
| CCGCTGGGATGCAGGTGCATGCCTCTAGCATGAAGTCAGACAA |
| >GS_C123 (*Cladocopium*) |
| AACCAATGGTCTCCTCAACGTGCGTTGCACTCTTGGGATTTCCTGAGAGTATGTCTGCTT |
| CAGTGCTTAACTTGCCCCAACTTTGCAAGCAGATGTGTTTCTGCCTTGCGTTCTTATGAG |
| CTATTGCCCTCTGAGCCAATGGCTTGTTAATTGCTTGGTTCTTGCAAAATGCTTTGCGCG |
| CTGTTATTCAGGTTTCTGCCTTCGTGGTTTTACTTGAGTGACGCTGCTCATGCTTGCAAT |
| TGCTGGGATGCAGGTGCATGCCTCTAGCATGAAGTCAGACAA |
| >GS_C124 (*Cladocopium*) |
| AACCAATGGCCTCCTGAACGTGCGTTGCACTCTTGGGCTTTCCTGAGAGTATGTCTGCTT |
| CAGTGCTTAACTTGTCCCAACTTTGCAAGCAGGACGTGTTTCTACCTTGCATTCTTATGA |
| GCTATTGCCCTCTGAGCTAATGGCTTGTTAAATTGCTTGGTTCTTGCAAAATGCTTTGCG |
| CGCTGTTATTCAAGTTTCTACCTTCGTGGTTTTATTTGAGTGACGCTGCTCATGCTTGCA |
| ACCGCTGGGATGCAGGTGCATGCCTCTAGCATGAAGTCAGACAA |
| >GS_C125 (*Cladocopium*) |
| ACGTGCGTTGCACTCTCGGGATTTCCTGAGAGCATGTCTGCTTCAGTGCTTAGCTTGCCC |
| CAACTTTGCAAGCAGGATGTGTTTCTGCCTTGCGTTCTTATGAGTTATTGCCCTCTGAGG |
| CAATGGCTTGTTAATTGTTTGGTTCTTGCAAAATGCTTTGCGCGCTGTTATTCAGGTTTC |
| TACCTTCGTGGTTTTACTTGAGTGACGCTGCTCATGCTTGCAACCGCTGGGATGTAGGTG |
| CATGCCTCTAGCATGAAGTCAGACAA |
| >GS_C126 (*Cladocopium*) |
| ACGTGCGTTGCACTCTTGGGATTTCCTGAGAGCATGTCTGCTTCAGTGCTTAGCTTGCCC |
| CAACTTTGCAAGCAGGATGTGTTTCTGCCTTGCGTTCTTATGAGTTATTGCCCTCTGAGG |
| CAATGGCTTGTTAATTGTTTGGTTCTTGCAAAATGCTTTGCGCGCTGTTATTCAGGTTTC |
| TACCTTCGTGGTTTTACTTGAGTGACGCTGCTCATGCTTGCAACCGCTGGGATGTAGGTG |
| CATGCCTCTAGCATGAAGTCAGACAA |
| >GS_C128 (*Cladocopium*) |
| AATCAATGGCCCCCTGAACGGGTGTTGCACTCTTGGGATTTCCTGAGAGTATGTCTGCTT |
| CAGTGCTTAACTTGCCCCAACTTTGCAAGCAGGATGTGTTTCTGCCTTGCGTACTTATGA |
| ACTATTGCCCTCTGAGTCAATGGCTTGTTAATTGCTTGGTTCTTGCAAAATGCTTTGTGC |
| GCTGTTATTCACGTTTCTACCTTCGTGGTTTTACTTGAGTGACGCTGCTCATGCTTGCAA |
| CCGCTGGGATGCTGGGATGCAGGTGCATGCCTCTAGCATGAAGTCAGACAA |
| >LJ_C160 (*Cladocopium*) |
| GTCTGACTTCATGCTAGAGGCATGCACCTGCATCCCAGCGGTTGCAAGCATGAGCAGCGTCACTCAAGTAAAACCACGAAGGTAGAAACTTGAATAACAGCGCGCAAAGCATTTTGCAAGAACCAAGCAATTAACAAGCCATTGGCTCAGAGGGCAATAGCTCATAAGAACGCAAGGCAGAAATGCTTGCAAAGTTGGGGCAAGTTAAGCACTGAAGCAGACATACTCTCAGGAAATCCCAAGAGTGCAACGCACGTTCAGGAGGCCATTGTTT |
| >LJ_C161 (*Cladocopium*) |
| GTCTGACTTCATGCTAGAGGCATGCACCTGCATCCCAGCGGTTGCAAGCATGAGCAGCGTCACTCAAGTAAAACCACGAAGGTAGAAACCTGAATAACAGCGCGCAAAGCATTTTGCAAGAACCAAGCAATTAACAAGCCATTGGAGGGCAATAGCTCATAAGAACGCAAGGCAGAAACACATCCTGCTTGCAAAGTTGGGGCAAGTTAAGCACTGAAGCAGACATACTCTCAGGAAATCCCAAGAGTGCAACGCACGTTCAGGAGGCCATTGGTTCACGGAGTTCTGCAATTCA |
| >LJ_C163 (*Cladocopium*) |
| GAGCAGCGTCACTCAAGTAAAACCAAGAAGGTAGAAACTTGAATAACAGCGCGCAAAGCATTTTGCAAGAACCAAGCAATTAACAAGCCATTGGCTCAGAGGGCAATAGCTCATAAGAACGCAAGGCAGAAACACATCTTGCAAAGTTGGGGCAAGTTAAGCACTGAAGCAGACATACTCTCAGGAAATCCCAAGAGTGCAACGCACGTTCAGGAGGCCATTGGTT |
| >LJ_C163a (*Cladocopium*) |
| GTCTGACTTCATGCTAGAGGCATGCACCTGCATCCCAGCGGTTGCAAGCATGAGCAGCGTCACTCAAGTAAAACCAAGAAGGTAGAAACTTGAATAACAGCGCGCAAAGCATTTTGCAAGAACCAAGCAATTAACAAGCCATTGGCTCAGAGGGCAATAGCTCATAAGAACACAAGGCAGAAACACATCTTGCAAAGTTGGGGCAAGTTAAGCACTGAAGCAGACATACTCTCAGGAAATCCCAAGAGTGCAACGCACGTTCAGGAGGCCATTGGTT |
| >LJ_C163b (*Cladocopium*) |
| GTCTGACTTCATGCTAGAGGCATGCACCTGCATCCCAGCGGTTGCAAGCATGAGCAGCGTCACTCAAGTAAAACCAAGAAGGTAGAAACCTGAATAACAGCGCGCAAAGCATTTTGCAAGAACCAAGCAATTAACAAGCCATTGGCTCAGAGGGCAATAGCTCATAAAAACGCAAGGCAGAAACACATCTTGCAAAGTTGGGGCAAGTTAAGCACTGAAGCAGACATACTCTCAGGAAATCCCAAGAGTGCAACGCACGTTCAGGAGGCCATTGGTTCACGGAGTTCTGCAATTCAC |
| >GS_D1 (*Durusdinium*) |
| AACCAATGGCCCCCTGAACGCGCATTGCACTCTTGGGACTTCCTGAGAGTATGTTTGCTT |
| CAGTGCTTATTTTACCTCCTTGCAAGGTTCTGTCGCAACCTTGTGCCCTGGCCAGCCATG |
| GGTTAACTTGCCCATGGCTTGCTGAGTAGTGATCTTTTAGAGCAAGCTCTGGCACGCTGT |
| TGTTTGAGGCAGCCTATATTGAGGCTATTTCAAATGACGTTGCTACAAGCTTGATGTGTC |
| CTTCTGCGCCGTTGCGCATCCCATAGCATGA |
| >GS_D1a (*Durusdinium*) |
| AACCAATGGCCCCCTGAACGCGCATTGCACTCTTGGGACTTCCTGAGAGTATGTTTGCTT |
| CAGTGCTTATTTTACCTCCTTGCAAGGTTCTGTCGCAACCTTGTGCCCTGGCCAGCCACG |
| GGTTAACTTGCCCATGGCTTGCTGAGTAGTGATCTTTTAGAGCAAGCTCTGGCACGCTGT |
| TGTTTGAGGCAGCCTATATTGAGGCTATTTCAAATGACGTTGCTACAAGCTTGATGTGTC |
| CTTCTGCGCCGTTGCGCATCCCATAGCATGA |
| >GS_D1.1 (*Durusdinium*) |
| AACCAATGGCCTCCTGAACGTGCATTGCACTCTTGGGATTTCCTGAGAGTATGTTTGCTT |
| CAGTGCTTAGTTTGGTCAACTTTGTTTGGATCCTGCTCCTGGGAACTGAGCGCCTCTGTG |
| AATCATTGGAGCAAACAGGACTTTGTCTCTGTTTTTTTGCCAATGGTTTGCTTGTCTCTA |
| CAATCCTTTGTGTAGCGCAAGCATATGTGCGTTGTTGTTTGATACATTTATCTCTTTGTA |
| GCATAAATGCTTTCTGCACGAAGCACATTGATATGCTTGCTACTGCTACTGTGCTGCGGA |
| GAGTTGGTTTTTGCATGTGTACCAGGTGTATAGTATGCTTATACTCTCTTGCACAACCCA |
| TAGCATGA |
| >GS_D1.2 (*Durusdinium*) |
| AACCAATGGCCTCCTGAACGCGCATTGCACTCTTGGGATTTCCTGAGAGTATGTTTGCTT |
| CAGTGCTTAGTTTGGTCAACTTTGTTTGGATCCTGCTTCTGGGAACCAAGCACCTCTGTG |
| AATCATTGGAACAAACAGGACTTCGTCTCTGTTTTGCCAATGGTTTGCTTGTCTCTTCAA |
| TCCTTTGTGTAGCACAAGCATATGTGCGTTGTTGTTTGATACATTTATATCTCTTTGTAG |
| TATAGATGCTATCTGCACGAAGCACATCTGATATGCTTGCTACTGCTATTGTGCTGCTGA |
| GAATTCTTATATGCATATGTACCATGTGTATATGATGTCTATATGATTTCTCTCCCTTGC |
| ACAACCCATAGCATGA |
| >GS_D1.6 (*Durusdinium*) |
| AACCAATGGCCCCCTGAACGCGCATTGCACTCTTGGGACTTCCTGAGAGTATGTTTGCTT |
| CAGTGCTTATTTTACCTCCTTGCAAGGTTCTGTCGCAACCTTGTGCCCTGGCCAGCCATG |
| GGTTAACTTGCCCATGGCTTGCTGAGTAGTGATCTTTTAGAGCAAGCTCTGGCACGCTGT |
| TGTTTGAGGCAGCCTATATTGAGGCTATTTCAAATGACGTTGCTACAAGCTTGATGTGTC |
| CTTCTGCGCCGTTGCGCAACCCATAGCATGA |
| >GS_D2 (*Durusdinium*) |
| AACCAATGGCCCCCTGAACGCGCATTGCACTCTTGGGACTTCCTGAGAGTATGTTTGCTT |
| CAGTGCTTATTTTACCTCCTTGCAAGGACCTTGTGCCCTGGCCAGCCACGGGTTAACTTG |
| CCCATGGCTTGCTGAGTAGTGATCTTTTAGAGCAAGCTCTGGCACGCTGTTGTTTGAGGC |
| AGCCTATATTGAGGCTATTTCAAATGACGTTGCTACAAGCTTGATGTGTCCTTCTGCGCC |
| GTTGCGCATCCCATAGCATGA |
| >GS_D2.2 (*Durusdinium*) |
| AACCAATGGCCCCGTGAACGCGCATTGCACTCTTGGGACTTCCTGAGAGTATGTTTGCTT |
| CAGTGCTTATTTTACCTCCTTGCAAGGTTCTGTCGCAACCTTGTGCCCTGGCCAGCCACG |
| GGTTAACTTGCCCATGGCTTGCTGAGTAGTGATCTTTTAGAGCAAGCTCTGGCACGCTGT |
| TGTTTGAGGCAGCCTATATTGAGGCTATTTCAAATGACGTTGCTACAAGCTTGATGTGTC |
| CTTCTGCGCCGTTGCGCATCCCATAGCATGA |
| >GS_D3 (*Durusdinium*) |
| AACCAATGGCCCCCTGAACGCGCATTGCACTCTTGGGACTTCCTGAGAGTATGTTTGCTT |
| CAGTGCTTATTTTACCTCTTTGCAAGGTTCTGTCGCAACCTTGTGCCCTGGCCAGCCATG |
| GGTTAACTTGCCCATGGCTTGCTGAGTAGTGATCTTTTAGAGCAAGCTCTGGCACGCTGT |
| TGTTTGAGGCAGCCTATATTGAGGCTATTTCAAATGACGTTGCTACAAGCTTGATGTGTC |
| CTTCTGCGCCGTTGCGCATCCCATAGCATGA |
| >GS_D4 (*Durusdinium*) |
| AACCAATGGCCCCCTGAACGCGCATTGCACTCTTGGGACTTCCTGAGAGTATGTTTGCTT |
| CAGTGCTTATTTTACCTCCTTGCAAGGTTCTGTGCGCAACCTTGTGCCCTGGCCAGCCAC |
| GGGTTAACTTGCCCATGGCTTGCTGAGTAGTGATCTTTTAGAGCAAGCTCTGGCACGCTG |
| TTGTTTGAGGCAGCCTATATTGAGGCTATTTCAAATGACGTTGCTACAAGCTTGATGTGT |
| CCTTCTGCGCCGTTGCGCATCCCATAGCATGA |
| >GS_D5 (*Durusdinium*) |
| AACCAATGGCCCCCTGAACGCGCATTGCACTCTTGGGACTTCCTGAGAGTATGTTTGCTT |
| CAGTGCTTGTTTTACCTCCTTGCAAGGTTCTGTCGCAACCTTGTGCCCTGGCCAGCCACG |
| GGTTAACTTGCCCATGGCTTGCTGAGTAGTGATCTTTTAGAGCAAGCTCTGGCACGCTGT |
| TGTTTGAGGCAGCCTATATTGAGGCTATTTCAAATGACGTTGCTACAAGCTTGATGTGTC |
| CTTCTGCGCCGTTGCGCATCCCATAGCATGA |
| >GS_D6 (*Durusdinium*) |
| AACCAATGGCCCCCTGAACGCGCATTGCACTCTTGGGACTTCCTGAGAGTATGTTTGCTT |
| CAGTGCTTATTTTACCTCCTTGCAAGGTTCTGTCGCAACCTTGTGCCCTGGCCAGCCACG |
| CGTTAACTTGCCCATGGCTTGCTGAGTAGTGATCTTTTAGAGCAAGCTCTGGCACGCTGT |
| TGTTTGAGGCAGCCTATATTGAGGCTATTTCAAATGACGTTGCTACAAGCTTGATGTGTC |
| CTTCTGCGCCGTTGCGCATCCCATAGCATGA |
| >GS_D7 (*Durusdinium*) |
| AACCAATGGCCCCCTGAACGTGCATTGCACTCTTGGGACTTCCTGAGAGTATGTTTGCTT |
| CAGTGCTTGTTTTACCTCCTTGCAAGGTTCTGTCGCAACCTTGTGCCCTGGCCAGCCACG |
| GGTTAACTTGCCCATGGCTTGCTGAGTAGTGATCTTTTAGAGCAAGCTCTGGCACGCTGT |
| TGTTTGAGGCAGCCTATATTGAGGCTATTTCAAATGACGTTGCTACAAGCTTGATGTGTC |
| CTTCTGCGCCGTTGCGCATCCCATAGCATGA |
| >GS_D8 (*Durusdinium*) |
| AACCAATGGCCCCCTGAACGCGCATCGCACTCTTGGGACTTCCTGAGAGTATGTTTGCTT |
| CAGTGCTTATTTTACCTCCTTGCAAGGTTCTGTCAACCTTGTGCCCTGGCCAGCCACGGG |
| TTAACTTGCCCATGGCTTGCTGAGTAGTGATCTTTTAGAGCAAGCTCTCTGGCACGCTGT |
| TGTTTGAGGCAGCCTGTATTGAGGCTATTTCAAATGACGTTGCTACAAGCTTGATGTGTC |
| CTTCTGCGCCGTTGCGCATCCCATAGCATGA |
| >GS_D9 (*Durusdinium*) |
| AACCAATGGCCCCCTGAATGCGCATTGCACTCTTGGGACTTCCTGAGAGTATGTTTGCTT |
| CAGTGCTTATTTTACCTCCTTGCAAGGTTCTGTCGCAACCTTGTGCCCTGGCCAGCCACG |
| GGTTAACTTGCCCATGGCTTGCTGAGTAGTGATCTTTTAGAGCAAGCTCTGGCACGCTGT |
| TGTTTGAGGCAGCCTATATTGAGGCTATTTCAAATGACGTTGCTACAAGCTTGATGTGTC |
| CTTCTGCGCCGTTGCGCATCCCATAGCATGA |
| >GS_D10 (*Durusdinium*) |
| AACCAATGGCCCCCTGAATGCGCATTGCACTCTTGGGACTTCCTGAGAGTATGTTTGCTT |
| CAGTGCTTATTTTACCTCCTTGCAAGGTTCTGTCGCAACCTTGTGCCCTGGCCAGCCATG |
| GGTTAACTTGCCCATGGCTTGCTGAGTAGTGATCTTTTAGAGCAAGCTCTGGCACGCTGT |
| TGTTTGAGGCAGCCTATATTGAGGCTATTTCAAATGACGTTGCTACAAGCTTGATGTGTC |
| CTTCTGCGCCGTTGCGCATCCCATAGCATGA |
| >GS_D11 (*Durusdinium*) |
| AACCAATGGCCCCCTGAACGCGCATTGCACTCTTGGGACTTCCTGAGAGTATGTTTGCTT |
| CAGTGCTTATTTTACCTCCTTGCAAGGTTCTGTCGCAACCTTGTGCCCTGGCCAGCCATG |
| GGTTAACTTGCCCATGGCTTGCTGAGTAGTGATCTTTTAGAGCAAGCTCTGGCACGCTGT |
| TGTTTGAGGCAGCCTATATTGAGGCTATTTCAAATGACAAGCTACAAGCTTGATGTGTCC |
| TTCTGCGCCGTTGCGCATCCCATAGCATGA |
| >LJ_D17 (*Durusdinium*) |
| GTTTGACTTCATGCTATGGGATGCGCAACGGCGCAGAAGGACACATCAAGCTTGTAGCAACGTCATTTGAAATAGCCTCAATATAGGCTGCCTCAAACAACAGCGTGCCAGAGCTTGCTCTAAAAGATCACTACTCAGCAAGCCATGGGCAAGTTAACCCATGGCTGGCCAGGGCACAAGGTTGCGACAGAACCTTGCAAGGAGGTAAAATAAGCACTGAAGCAAACATACTCTCAGGAAGTCCCAAGAGTGCAATGCACGTTCAGGGGGCCATTGGTT |
| >GS_E1 (*Effrenium*) |
| AACCAATAGCACCCTGAACTCGCATTGCACTCTTGGGACACGCCTGAGAGTATGTCTGCT |
| TCAGTGCTTTTCATATCTTCGCAGTGCGGGCTTCCTGGAGAAGCCTTGAGCCTCTTTGTG |
| CGCTGCTGCATCAGAATTTGCAGCGGCGCGCTGAACACAAACCGGGAGGTAAGCTGGACT |
| GATTTGTCGCGCATCACTGGGCACGTGTGTCCGTTTTGGCCCAATCATGCCAGCCTGCCA |
| AGCAATTGGTGCTCAAATACCAATCTTAGCATGAAGTCGAGACAA |
| >GS_F1 (*Fugacium*) |
| AACCAATGGCCTCCTGAACGTACGTTGCACTCTTGGGATTTCCTGAGAGTATGTCTGCTT |
| CAGTGCTTAGCTTGCCCAATCTTGCGGATAGATTTTGTTTCTGTCTTGCGCCCCTGTGAG |
| CCATTGAAACTCTAGTCAATGGCTTATTGAATGAGTTGGTCTTGCAAAAGCTTTGCGCGA |
| TGCTATTCAAGATTCCACCTTGAAATGGTATTTCTTGAGTGACGCTGCTTATGCTTGCAA |
| CTGCTGGGATGCTAGCGCATGCCTCTAGCATGAAGTCAGACAAGTGAA |
| >GS_F2 (Clade F) |
| AGTCAATGGCCTCCTGAACGTACGTTGCACTCTTGGGGTTTCCTGAGAGTATGTCTGCTT |
| CAGTGCTTAGCTTGCCCAACCATGCAAGCAGTTTTGTCGTCTGCCTTGCGCTCCTACGAG |
| CCATTGTGACTACCAATGGCTTGTTGAATGTTCTTACTTGCATATGAAAGTTTTGCGCGC |
| TATTGTCCAAGTTTTACCTTCTCCGGTATTGCTTGAGTGACGCTGCTAACGCTTGCAACT |
| GCCGGGCTGCTGGTGCATGCCTCTAGCATGAAGTCAGGCAAGTGAA |
| >GS_F2a (Clade F) |
| AGTCAATGGCCTCCTGAACGTACGTTGCACTCTTGGGGTTTCCTGAGAGTATGTCTGCTT |
| CAGTGCTTAGCTTGCCCAACCATGCAAGCAGTTTTGTCGTCTGCCTTGCGCTCCTACGAG |
| CCATTGTGACTACCAATGGCTTGTTGAATRATCTTACTTGCATATGAAAGTTTTGCGCGC |
| TATTGTCCAAGTTTTACCTTCTCCGGTATTGCTTGAGTGACGCTGCTAACGCTTGCAACT |
| GCCGGGCTGCTGGTGCATGCCTCTAGCATGAAGTCAGGCAAGTGAA |
| >GS_F2b (Clade F) |
| AGTCAATGGCCTCCTGAACGTACGTTGCACTCTTGGGGTTTCCTGAGAGTATGTCTGCTT |
| CAGTGCTTAGCTTGCCCAACCACGCAAGCAGTTTTGTCGTCTGCCTTGCGCTCCTACGAG |
| CCATTGTGACTACCAATGGCTTGTTGAATGTTTTTACTTGCATATGAAAGTTTTGCGCGC |
| TATTGTTCAAGTTTTACCTTCGCCGGTATTGCTTGAGTGACGCTGCTAACGCTTGCAACT |
| GCTGGGCTGCTGGTGCATGCCTCTAGCATGAAGTCAGGCAAGTGAA |
| >GS_F3.1 (*Freudenthalidium*) |
| AATCAATGGCCTCCTGAACGTACGTTGCACTCTCGGGGTTTCCTGAGAGTATGTCTGCTT |
| CAGTGCTTAGCTTGCCCGACCTTGCACAAGCGGCTTTGTGTCTGCTTTGCGCTCTTATGA |
| GCCATTGGTTTGTGAAGCCTATGGCTCGTTAAATCGTTGTGTTTGCGAACGAAAAGCTTT |
| GCGCGCTGTTATTCGAGTAAAGTGCCCTTCGGGGTTCTGGGCTTGAGTGACGCTGCTCTT |
| GCTTGCAACTGCCAGGCTGCTGTGGTGCACGCCTCTAGCATGAAGTCAGGCAAGTGAA |
| >GS_F3.1a (*Freudenthalidium*) |
| AATCAATGGCCTCCTGAACGTACGTTGCACTCTCGGGGTTTCCTGAGAGTATGTCTGCTT |
| CAGTGCTTAGCTTGCCCGACCTTGCACAAGCGGCTTTGAGTCTGCTTTGCGCTCTTATGA |
| GCCATTGGTTTGTGAAGCCTATGGCTCGTTAAATCGTTGTGTTTGCGAACGAAAAGCTTT |
| GCGCGCTGTTATTCGAGTAAAGTGCCCTTTGGGGTTCTGGGCTTGAGTGACGCTGCTCTT |
| GCTTGCAACTGCCAGGCTGCTGTGGTGCACGCCTCTAGCATGAGGTCAGGCAAGTGAA |
| >GS_F3.2 (*Freudenthalidium*) |
| AATCAATGGCCTCCTGAACGTACGTTGCACTCTCGGGGTTTCCTGAGAGTATGTCTGCTT |
| CAGTGCTTAGCTTGCCCAACCTTGCAAAAGCGGCTTTGTGTCTGCTTTGTGCCCTTATGA |
| GCCATTGGTTTCCAACCAATGGCTTGTTAAATCATTGTGTTTGCAAATGAAAAGCTTTGC |
| GCGCTGTTATTCAAGTAAGTACCCTTCAGGGTTCTAAACTTGAGTGACGCTGCTCTTGCT |
| TGCAACTGCCAGGCTGCTGGTGCATGCCTCTAGCATGAAGTCAGGCAAGCGAA |
| >GS_F3.2a (*Freudenthalidium*) |
| AATCAATGGCCTCCTGAACGTACGTTGCACTCTCGGGGTTTCCTGAGAGTATGTCTGCTT |
| CAGTGCTTAGCTTGCCCAACCTTGCAAAAGCGGCTTTGTGTCTGCTTTGTGCTCTTATGA |
| GCCATTGGTTTCCAGCCAATGGCTTGTTGAATCATTGTGTTTGCAAATGACAAGCTTTGC |
| GCGCTGTTATTCAAGTAAGTTCCCTTCAGGGTTCTAAACTTGAGTGACGCTGCTCTTGCT |
| TGCAACTGCCAGGCTGCTGGTGCATGCCTCTAGCATGAAGTCAGGCAAGCGAA |
| >GS_F3.3 (*Freudenthalidium*) |
| AATCAATGGCCTCCTGAACGTACGTTGCACTCTTGGGTTTTCCTGAGAGTATGTCTGCTT |
| CAGTGCTTAGCTTGCCCAACTTTGCAAGAAGCATTACTTGCTTCCTGCGCTCGTATGAGC |
| CATTTGCTTTTTTGCTGATGGCTTGTTGAGTAATAGGTGTTGCAAAAAAAAGCTTTGCGC |
| GCTGTTGTTCAAGTACGTACCCTTGACAGGTCAGCTTGAGTGACGCTGCTCGTGCTTGCA |
| ACTGCCAGGGTGCTGGCGCATGCCTCTAGCATGAAGTCAGGCAAGTGAA |
| >GS_F3.4 (*Freudenthalidium*) |
| AATCAATGGCCTCCTGAACGTACGTTGCACTCTTGGGTTTTCCTGAGAGTATGTCTGCTT |
| CAGTGCTTAGCTTGCCCAACTTTGCAGGAAACATTATTTGCTTTCTGCGCTTGTATGAGC |
| CATTTGCTTTTTAGCAGATGGCTCGTTGAGTAATCAGTGTTGCAAATAAAAGCTTTGCGC |
| GCTATTGTTCAAGCACGTACCTTTGACGGGTTAGCTTGAGTGACGCTGCTTGTGCTTGCA |
| ATTGCCAGGCTGCTGCCGCATGCCTCTAGCATGAAGTCAGGCAAGTGGA |
| >GS_F3.4a (*Freudenthalidium*) |
| AATCAATGGCCTCCTGAACGTACGTTGCACTCTTGGGTTTTCCTGAGAGTATGTCTGCTT |
| CAGTGCTTAGCTTGCCCAACTTTGCAGGAAACATTATTTGCTTTCTGCGCTTGTATGAGC |
| CATTTGCTTTTTAGCAGATGGCTCGCTGAGTAATCAGTGTTGCAAATAAAAGCTTTGCGC |
| GCTATTGTTCAAGCATGTACCTTTGACGGGTTAGCTTGAGTGACGCTGCTTGTGCTTGCA |
| ATTGCCAGGCTGCTGCCGCATGCCTCTAGCATGAAGTCAGGCAAGTGGA |
| >GS_F3.4b (*Freudenthalidium*) |
| AATCAATGGCCTCCTGAACGTACGTTGCACTCTTGGGTTTTCCTGAGAGTATGTCTGCTT |
| CAGTGCTTAGCTTGCCCAACTTTGCAGGAAACATTATTTGCTTTCTGCGCTTGTATGAGC |
| CATTTGCTTTTTAGCAGATGGCTCGTTGAGTCATCAGTGTTGCAAATAAAAGCTTTGCGC |
| GCTATTGTTCAAGCACGTACCTTTGACGGGTTAGCTTGAGTGACGCTGCTTGTGCTTGCA |
| ATTGCCAGGCTGCTGCCGCATGCCTCTAGCATGAAGTCAGGCAAGTGGA |
| >GS_F4.1 (Clade F) |
| AACCAATGGCCTCCTGAACGTACGTTGCACTCTTGGGATTTCCTGAGAGTATGTCTGCTT |
| CAGTGCTTAGCTTTCTCAACTTTGCAAAGCAATTCCCTTATTGCCTTGCGTTCCTATGAG |
| CCATTGACAATCATGGTCCATGGCTTGTTGACCAACCGGTTCCTGCAAAGCTTTGCGCGC |
| TGTTGTTCAAGCTTTTCTGCTTTGAGTGACGCTGCTGACGCTTGCAACCGCCGGGGTGCC |
| CATGCACATCTCTAGCATGAAGTCAGACAAGCAAA |
| >GS_F4.1a (Clade F) |
| AACCAATGGCCTCCTGAACGTACGTTGCACTCTTGGGATTTCCTGAGAGTATGTCTGCTT |
| CAGTGCTTAGCTGTCTCAACTTTGCAAAGCAATTCCCTGATTGCCTTGCGTTCCTATGAG |
| CCATCGACAATCATGGTCCATGGCTTGTTGACCAACCGGTTCCTGCAAAGCTTTGCGCGC |
| TGTTGTTCAAGCTTTTCTGCTTTGAGTGACGCTGCTGACGCTTGCAACCGCCGGGGTGCC |
| CATGCACATCTCTAGCATGAAGTCAGACAAGTAAA |
| >GS_F4.2 (Clade F) |
| AACCAATGGCCTCCTGAACGTACGTTGCACTCTCGGGATTTCCTGAGAGTATGTCTGCTT |
| CAGTGCTTAGCTTTCTCAACCTTGCAAAGCAATTCTTTCTTTGCCTTGCGTTCCTATGAG |
| CCATTGACAATCATCGTCAATGGCTTGTTGACCAACCGGTTCCTGCAAAGCTTTGCGCGC |
| TGTTGTTCAAGCTTCTCTGCTTTGAGTGACGCTGCTCATGCTTGCAACCGCCGGGGTGCC |
| GATGCACAACTCTAGCATGAAGTCAGACAAGTGAA |
| >GS_F4.1b (Clade F) |
| AACCAATGGCCTCCTGAACGTACGTTGCACTCTTGGGATTTCCTGAGAGTATGTCTGCTT |
| CAGTGCTTAGCTTTCTCAACCTTGCAAAGCAATTCCCTTATTGCTTTGCGTTCCTATGAG |
| CCATTGACAATCATGGTCCATGGCTTGTTGACCAACCGGTTCCTGCAAAGCTTTGCGCGC |
| TGTTGTTCAAGCTTTTCTGCTTTGAGTGACGCTGCTGACGCTTGCAACCGCCGGGGTGCC |
| CATGCACATCTCTAGCATGAAGTCAGACAANNNNN |
| >GS_F4.2b (Clade F) |
| AACCAATGGCCTCCTGAACGTACGTTGCACTCTCGGGATTTCCTGAGAGTATGTCTGCTT |
| CAGTGCTTAGCTTTCTCAACCTTGCAAAGCAATTCTTTTTTGCTTTGCGTTCCTATGAGC |
| CATTGACAATCATGGCCAATGGCTTGTTGACCAACCGGTTCCTGCAAAGCTTTGCGCGCT |
| GTTGTTCAAGCTTCTCTGCTTTGAGTGACGCTGCTCATGCTTGCAACCGCCGGGGTGCCG |
| ATGCACAACTCTAGCATGAAGTCAGACAAGTGAA |
| >GS_F4.2a=F4.2c (Clade F) |
| AACCAATGGCCTCCTGAACGTACGTTGCACTCTCGGGATTTCCTGAGAGTATGTCTGCTT |
| CAGTGCTTAGCTTTCTAAACCTTGCAAAGCAATTCTTTTTTTGCTTTGCGTTCCTATGAG |
| CCATTGACAATCATGGCCAATGGCTTGTTGACCAACCGGTTCCTGCAAAGCTTTGCGCGC |
| TGTTGTTCAAGCTTCTCTGCTTTGAGTGACGCTGCTCATGCTTGCAACCGCCGGGGTGCC |
| GATGCACAACTCTAGCATGAAGTCAGACAAGTGAA |
| >GS_F4.3 (Clade F) |
| AACCAATGGCCTCCTGAACGTACGTTGCACTCTCGGGATTTCCTGAGAGTATGTCTGCTT |
| CAGTGCTTAGCTTTCTCAACCTTGCAAAGCAATGTTTCTTTTTGCCTTGCGTTCCTATGA |
| GCCATTGACAATCATGGTCAATGGCTTGTTGACCAACCGGTTCCTGCGAAGCTTTGCGCG |
| CTGTTGTTCAAGCTTTTCTGCTTTGAGTGACGCTGCTCATGCTTGCAACCGCCGGGGTGC |
| CGACGCACATCTCTAGCATGAAGTCAGACAAGTGAA |
| >GS_F4.3a (Clade F) |
| AACCAATGGCCTCCTGAACGTACGTTGCACTCTCGGGATTTCCTGAGAGTATGTCTGCTT |
| CAGTGCTTAGCTTTCTCAACCTTGCAAAGCAATGTTTCTTTTTGCTTTGCGTTCCTATGA |
| GCCATTGACAATCATGGTCAATGGCTTGTTGACCAACCGGTTCCTGCGAAGCTTTGCGCG |
| CTGTTGTTCAAGCTTTTCTGCTTTGAGTGACGCTGCTCATGCTTGCAACCGCCGGGGTGC |
| CGACGCACATCTCTAGCATGAAGTCAGACAAGTGAA |
| >GS_F4.3b (Clade F) |
| AACCAATGGCCTCCTGAACGTACGTTGCACTCTCGGGATTTCCTGAGAGTATGTCTGCTT |
| CAGTGCTTAGCTTTCTCAACCTTGCAAAGCAATGTTTCTTTTTGCCTTGCGTTCCTATGA |
| GCCATCGACAATCATGGTCAATGGCTTGTTGACCAACCGGTTCCTGCGAAGCTTTGCGCG |
| CTGTTGTTCAAGCTTTTCTGCTTTGAGTGACGCTGCTCATGCTTGCAACCGCCGGGGTGC |
| CGACGCACATCTCTAGCATGAAGTCAGACAAGTGAA |
| >GS_F4.3c (Clade F) |
| AACCAATGGCCTCCTGAACGTACGTTGCACTCTCGGGATTTCCTGAGAGTATGTCTGCTT |
| CAGTGCTTAGCTTTCTCAACCTTGCAAAGCAATGTTTCTTTTTGCCTTGCGTTCCTATGA |
| GCCATTGACGACCATGGTCAATGGCTTGTTGACCAACCGGTTCCTGCGAAGCTTTGCGCG |
| CTGTTGTTCAAGCTTTTCTGCTTTGAGTGACGCTGCTCATGCTTGCAACCGCCGGGGTGC |
| CGACGCACATCTCTAGCATGAAGTCAGACAAGTGAA |
| >GS_F4.4 (Clade F) |
| AACCAATGGCCTCCTGAACGTACGTTGCACTCTCGGGATTTCCTGAGAGTATGTCTGCTT |
| CAGTGCTTAGCTTTCTCCACCTTGCAAGCAAATTGTGTTTTTGCCTTGCGTCCCTATGAG |
| CCATTGACGGTCACGTCCAATGGCTTGTTGACCAGTCGGTTCCTGCAAAGCTTTGCGCGC |
| TGTTGTTCAAGCTTTTTCTGCTTTGAGTGACGCTGCTCATGCTTGCAACCGCCGGGGTGC |
| TGATGCACATCTCTAGCATGAAGTCAGACAAGTGAA |
| >GS_F4.4a (Clade F) |
| AACCAATGGCCTCCTGAACGTACGTTGCACTCTCGGGATTTCCTGAGAGTATGTCTGCTT |
| CAGTGCTTAGCTTTCTCCACCTTGCAAGCAAATTTTGTTTTGCCTTGCGTTCCTATGAGC |
| CATTGACAATCACGGCCAATGGCTTGTTGACCAGCCGGTTCCTGCAAAGCTTTGCGCGCT |
| GTTGTTCAAGCTTTTTCTGCTTTGAGTGACGCTGCTCATGCTTGCAACCGCCGGGGTGCT |
| GATGCACATCTCTAGCATGAAGTCAGACAAGTGAA |
| >GS_F4.4b (Clade F) |
| AACCAATGGCCTCCTGAACGTACGTTGCACTCTCGGGATTTCCTGAGAGTATGTCTGCTT |
| CAGTGCTTAGCTTTCTCCACCTTGCAAGCAAATTGTGTTTTGCCTTGCGTTCCTATGAGC |
| CATTGACGATCACGTCCAATGGCTTGTTGACCAGCCGGTTCCTGCAAAGCTTTGCGCGCT |
| GTTGTTCAAGCTTTCCGCTTTGAGTGACGCTGCTCATGCTTGCAACCGCCGGGGTGCTGA |
| TGCACATCTCTAGCATGAAGTCAGACAAGTGAA |
| >GS_F4.5 (Clade F) |
| AACCAATGGCCTCCTGAACGTACGTTGCACTCTCGGGATTTCCTGAGAGTATGTCTGCTT |
| CAGTGCTTAGCTTTCTCAACCTTGCAAGCAAATTTTGATTTGCCTTGCGTTCCTATGAGC |
| CATTGGCAAGCATGGCCCAATGGCTCGTTGACCAGCCGGTTCCTGCAAAGCTTTGCGCGC |
| TGTTGTTCAAGCTTTCTTTCAGCTTTGAGTGACGCTGCTCACGCTTGCAACCGCAGGGGT |
| GCTGACGCACATCTCTAGCATGAAGTCAGACAAGTGAA |
| >GS_F4.6 (Clade F) |
| AACCAATGGCCTCCTGAACGTACGTTGCACTCTCGGGATTTCCTGAGAGTATGTCTGCTT |
| CAGTGCTTAGCTTTCTCAACCTTGCAAAGCAATTCTTCTTTTGCCTTGCGCTCCTATGAG |
| CCATTGACAATCATGGCCAATGGCTTGTTGACCAACCGGTTCCTGCAAAGCTTTGCGCGC |
| TGTTGTTCAAGCTTTTCTGCTTTGAGTGACGCTGCTCACGCTTGCAACCGCCGGGGTGCC |
| CATGCACATCTCTAGCATGAAGTCAGACAAGTACA |
| >GS_F4.7 (Clade F) |
| AACCAATGGCCTCCTGAACGTACGTTGCACTCTCGGGATTTCCTGAGAGTATGTCTGCTT |
| CAGTGCTTAGCTTTCTCAACCTTGCAAAGCATGCGTGTTTTTTTGCCTTGCGTTCCTACG |
| AGCCATTGACAACCACGGTCAGTGGCTTGTTGACCAACCGGTTCCTGCAAAGCTTTGCGC |
| TCTGTTGTTCAAGCTTTTCTGCTTTGAGTGACGCTGCTCACGCTTGCAACCGCCGGAGCG |
| CCGATGCACATCTCTAGCATGAAGTCAGACAAGTGAA |
| >GS_F4.8 (Clade F) |
| AACCAATGGCCTCCTGAACGTACGTTGCACTCTCGGGATTTCCTGAGAGTATGTCTGCTT |
| CAGTGCTTAGCTTTCTCAACCTTGCAAAGCAAAGTCTTTTCTTTGCTGTTGCGCTCCTAT |
| GGGCCATTGACAATCATGGTCAATGGCTTGTTGACTAACCGGTTCCTGCAAAGCTTTGCG |
| CGCTGTTGTTCAAGCTTCTCTGCTCTGAGTGACGCTGCTCACGCTTGCAACCGCCGGGGT |
| GCTGAAGCACAACTCTAGCATGAAGTCAGACAAGTGAA |
| >GS_F4.8a (Clade F) |
| AACCAATGGCCTCCTGAACGTACGTTGCACTCTCGGGATTTCCTGAGAGTATGTCTGCTT |
| CAGTGCTTAGCTTTCTCAACCTTGCAAAGCAAAGTCGTTTCTTTGCTGTTGCGCTCCTAT |
| GGGCCATTGACAATCATGGTCAATGGCTTGTTGACTAACCGGTTCCTGCAAAGCTTTGCG |
| CGCTGTTGTTCAAGCTTCTCTGCTCTGAGTGACGCTGCTCACGCTTGCAACCGCCGGGGT |
| GCTGAAGCACAACTCTAGCATGAAGTCAGACAAGTGAA |
| >GS_F4.8b (Clade F) |
| AACCAATGGCCTCCTGAACGTACGTTGCACTCTCGGGATTTCCTGAGAGTATGTCTGCTT |
| CAGTGCTTAGCTTTCTCAACCTTGCAAAGCAAAGTCGTTTCTTTGCTGTTGCGCTCCTAT |
| GGGCCATTGCCAATCATGGTCGATGGCTTGTTGACTAACCGGTTCCTGCAAAGCTTTGCG |
| CGCTGTTGTTCAAGCTTCTCTGCTCTGAGTGACGCTGCTCACGCTTGCAACCGCCGGGGT |
| GCTGAAGCACAACTCTAGCATGAAGTCAGACAAGTGAA |
| >GS_F4.8c (Clade F) |
| AACCAATGGCCTCCTGAACGTACGTTGCACTCTCGGGATTTCCTGAGAGTATGTCTGCTT |
| CAGTGCTTAGCTTTCTCAACCTTGCAAAGCAAAGTCTTTTCTTTGCTGTTGCGCTCCTGT |
| GGGCCATTGGACAATCATGGTCAATGGCTTGTTGACTAACCGGTTCCTGCAAAGCTTTGC |
| GCGCTGTTGTTCAAGCTTCTCTGCTCTGAGTGACGCTGCTCACGCTTGCAACCGCCGGGG |
| TGCTGAAGCACAACTCTAGCATGAAGTCAGACAAGTGAA |
| >GS_F5.1 (*Fugacium*) |
| AACCAATGGCCTCCTGAACGTACGTTGCACTCTTGGGATTTCCTGAGAGTATGTCTGCTT |
| CAGTGCTTAGCTTGCCCAATCTTGCGGATAGACTTTGTTTCTGTCTTGCGCCCCTGTGAG |
| CCATTGAGCGTCTAGTCAATGGCTTATTGAATGATTCGGTCTTGCAAAAGCTTTGCGCGC |
| TTCTATTCAAGATTCCACCTTGGAGTGGTATTGCTTGAGTGACGCTGCTCATGCTTGCAA |
| CTGCTGGGATGCTAACGCATGCCTCTAGCATGAAGTCAGACAAGCGAA |
| >GS_F5.1a (*Fugacium*) |
| AACCAATGGCCTCCTGAACGTACGTTGCACTCTTGGGATTTCCTGAGAGTATGTCTGCTT |
| CAGTGCTTAGCTTGCCCAATCTTGCGGATAGACTTTGTTTCTGTCTTGCGCCCCTGTGAG |
| CCATTGAGCGTTTAGTCAATGGCTTATTGAATGATTCGGTCTTGCAAAAGCTTTGCGCGC |
| TTCTATTCAAGATTCCACCTTGGAGTGGTATTGCTTGAGTGACGCTGCTCATGCTTGCAA |
| CTGCTGGGATGCTAACGCATGCCTCTAGCATGAAGTCAGACAAGCGAA |
| >GS_F5.1b (*Fugacium*) |
| AACCAATGGCCTCCTGAACGTACGTTGCACTCTTGGGATTTCCTGAGAGTATGTCTGCTT |
| CAGTGCTTAGCTTGCCCAATCTTGCGGATAGACTTTGTTTCTGTCTTGCGCCCCTGTGAG |
| CCATTGAGCGTTTAGTCAATGGCTTATTCAATGATTCGGTCTTGCAAAAGCTTTGCGCGC |
| TTCTATTCAAGATTCCACCTTGGAGTGGTATTGCTTGAGTGACGCTGCTCATGCTTGCAA |
| CTGCTGGGATGCTAACGCATGCCTCTAGCATGAAGTCAGACAAGCGAA |
| >GS_F5.1c (*Fugacium*) |
| AACCAATGGCCTCCTGAACGTACGTTGCACTCTTGGGATTTCCTGAGAGTATGTCTGCTT |
| CAGTGCTTAGCTTGCCCAATCTTGCGGATAGATTTTATTTCTGTCTTGCGCCCCTGTGAG |
| CCATTGAATGTCTAGTCAATGGCTTATTGAATGATTTGGTCTTGCAAAAGCTTTGCGCGC |
| TTCTATTCAAGATTCCACCTTGGAGTGGTATTGCTTGAGTGACGCTGCTCATGCTTGCAA |
| CTGCCGGGATGCTAACGCATGCCTCTAGCATGAAGTCAGACAAGCGAA |
| >GS_F5.2=F5.2a (*Fugacium*) |
| AACCAATGGCCTCCTGAACGTACGTTGCACTCTTGGGATTTCCTGAGAGTATGTCTGCTT |
| CAGTGCTTAGCTTGCCCAATCTTGCGGACAGATTTTGTTTCTGCCCTGCGCCCCTGTGAG |
| CCATTGAATGTCTACCCAATGGCTTATTGAATGATTTGGTCTTGCAAAAGCTTTGCGCGC |
| TGCTATTCAAGATTCCACCTTAAAGTGGTATTGCTTGAGTGACGCTGCTTATGCTTGCAG |
| CTGCTGGGATGCTAGCGCATGCCTCTAGCATGAAGTCAGACAAGCGAA |
| >GS_F5.2b (*Fugacium*) |
| AACCAATGGCCTCCTGAACGTACGTTGCACTCTTGGGATTTCCTGAGAGTATGTCTGCTT |
| CAGTGCTTAGCTTGCCCAATCTTGCGGACAGATTTTGTTTCTGTCCTGCGCCCCTGTGAG |
| CCATTGAATGTCTACCCAATGGCTTATTGAATGATTTGGTCTTGCAAAAGCTTTGCGCGC |
| TGCTATTCAAGATTCCACCTTAAAGTGGTATTGCTTGAGTGACGCTGCTTATGCTTGCAG |
| CTGCTGGGATGCTAGCGCATGCCTCTAGCATGAAGTCAGACAAGCGAA |
| >GS_F5.2c (*Fugacium*) |
| AACCAATGGCCTCCTGAACGTACGTTGCACTCTTGGGATTTCCTGAGAGTATGTCTGCTT |
| CAGTGCTTAGCTTGCCCAATCTTGCGGACAGATTTTGTTTCTGTCCTGCGCCCCTGTGAG |
| CCATTGAATGCCTACTCAATGGCTTATTGAATGATTTGGTCTTGCAAAAGCTTTGCGCGC |
| TGCTATTCAAGATTCCACCTTAAAGTGGTATTGCTTGAGTGACGCTGCTTATGCTTGCAG |
| CTGCTGGGATGCTAGCGCATGCCTCTAGCATGAAGTCAGACAAGCGAA |
| >GS_F5.2d (*Fugacium*) |
| AACCAATGGCCTCCTGAACGTACGTTGCACTCTTGGGATTTCCTGAGAGTATGTCTGCTT |
| CAGTGCTTAGCTTGCCCAATCTTGCGGACAGATTTTGTTTCTGTCCTGCGCCCCTGTGAG |
| CCATTGAATGCCTGCTCAATGGCTTATTGAATGATTTGGTCTTGCAAAAGCTTTGCGCGC |
| TGCTATTCAAGATTCCACCTTAAAGTGGTATTGCTTGAGTGACGCTGCTTATGCTTGCAG |
| CTGCTGGGATGCTAGCGCATGCCTCTAGCATGAAGTCAGACAAGCGAA |
| >GS_F5.2e (*Fugacium*) |
| AACCAATGGCCTCCTGAACGTACGTTGCACTCTTGGGATTTCCTGAGAGTATGTCTGCTT |
| CAGTGCTTAGCTTGCCCAATCTTGCGGACAGATTTTGTTTCTGTCTTGCGCCCCTGTGAG |
| CCATTGAATGTCTACTCAATGGCTTATTGAATGATTTGGTCTTGCAAAAGCTTTGCGCGC |
| TGCTATTCAAGATTCCACCTTAAAGTGGTATTGCTTGAGTGACGCTGCTTATGCTTGCAG |
| CTGCTGGGATGCTAGCGCATGCCTCTAGCATGAAGTCAGACAAGCGAA |
| >GS_F5.2f (*Fugacium*) |
| AACCAATGGCCTCCTGAACGTACGTTGCACTCTTGGGATTTCCTGAGAGTATGTCTGCTT |
| CAGTGCTTAGCTTGCCCAATCTTGCGGACAGATTTTGTTTCTGTCTTGCGCCCCTGTGAG |
| CCATTGAGCGTCTATTCAGTGGCTTATTGAATGATTTGGTCTTGCAAAAGCTTTGCGCGC |
| TGCTATTCAAGATTCCACCTTAAAGTGGTATTGCTTGAGTGACGCTGCTTATGCTTGCAA |
| CTGCTGGGATGCTAGCGCATGCCTCTAGCATGAAGTCAGACAAGCGAA |
| >GS_G1 (*Gerakladium*) |
| AACCAATGGCCTCCTGAACGCGCATTGCACTCTTGGGCTTTCCTGAGAGTATGTTTGCTT |
| CAGTGCTTCTTTTGCTCAACCCTTGCAAGGTCTGGCAGCGCAATGCCCCCTTGAGCCTAG |
| GCATGTCGTTGGCGCATCTGCCAATGACAAGCGACCTCCATGGCTTGTGCAAGCATGCAC |
| GTGCTTATTGTTGTTTTCAGAGCAAACTTCACCACCATGGGTGTGGGCAACGTGGCTGAT |
| GCTTGAGCACGCGCCGGTGCGTTGCTTGCACCTTCTCCATAGCATGAA |
| >GS_G2 (*Gerakladium*) |
| AACCAATGGCCTCCTGAACGCGCATTGCACTCTTGGGCTTTCCTGAGAGTATGTTTGCTT |
| CAGTGCTTCTTTTGCTCAACCTTGCAAGGTCTGGCAATGCAATGCCCCCTTGAGCCTCGG |
| CTTGTTGTTGCACATCTGCCAGTGACAAGCGACTTCCATGGCCTGTGCAAGCATGCACGT |
| GCTTTTTTGTCGTTTTCAGAGCAAACTTCTTCGCCATGGGCGGGGGCAATGTGGCTGATG |
| CTTGAGCACGCGCCGGTGCGTTGCTTGCACCTCCTCCATAGCATGAA |
| >GS_G2a (*Gerakladium*) |
| AACCAATGGCCTCCTGAACGCGCATTGCACTCTTGGGCTTTCCTGAGAGTATGTTTGCTT |
| CAGTGCTTCTTTTGCTCAACCTTGCAAGGTCTGGCAATGTAATGCCCCCTTGAGCCTCGG |
| CTTGTTGTTGCACATCTGCCAGTGACAAGCGACTTCCATGGCCTGTGCAAGCATGCACGT |
| GCTTTTTTGTCGTTTTCAGAGCAAACTTCTTCGCCATGGGCGGGGGCAATGTGGCTGATG |
| CTTGAGCACGCGCCGGTGCGTTGCTTGCACCTCCTCCATAGCATGAA |
| >GS_G3 (*Gerakladium*) |
| AACCAATGGCCTCCTGAACGCGCATTGCACTCTTGGGCTTTCCTGAGAGTATGTTTGCTT |
| CAGTGCTTCTTTTGCTCCACCGTTGCAAGGTTTGGCAGCGCAATGCCTCCTTGTGCCTCG |
| GCGTGTTGTTGGCGTCTCTGCCAACGACGTGCGACCAGCGTGGCCTTTGTGCAAGCATGC |
| ACGTGCTTTGTTGTTTCACTGCAGCCATTCTCCGGAATATGCGTGGGCGACGTGGCTGAT |
| GCTTGCGGACGCGCTACTGTGCTGCTTGCACTTCTTCCATAGCATGAA |
| >GS_G4 (*Gerakladium*) |
| AACCAATGGCCTCCTGAACGCGCATTGCACTCTTGGGCTTTCCTGAGAGTATGTTTGCTT |
| CAGTGCTTCTTCTGTTCCTCCATGGCGAGGGCTTTGGCAAGCTATGCCCCCTCCTGCCTT |
| GGCGTGCCGTTGGTGTGTTTGCCAATGACATGCGACATGCGTGGCCTTTGTGCAGGCAAG |
| CACGCGCGTTGTTGTTTCACGCACCATTCCTCAGAATGTCGTGTGTGTGTGGGCGACGTG |
| ACTGATGCTTGAGGACGCGCTGGAGTGCTGATGCACTTCCCATAGCATGAA |
| >GS_H1 (*Halluxium*) |
| AACCAATGGCCTCCTGAACGTGCGTTGCACTCTCGGGATTTCCTGAGAGTATGTCTGCTT |
| CAGTGCTTAGCTTACCCAACTTTGCAATCAGGCTGACTACCAAGCCTGCTTTTTGCGTTC |
| CTATGAGCTATTGCGCTTCCTTTGCCAATGGCTTGTTGATTGGTAGGTTCCTGCAAAATG |
| CTTTGCGCGCTGTTATTCAAGTTTCGCCTGCACGGCTTTGCTTGAGTGACGCTGCTCATG |
| CGTGCAACCGCTGGGATGCGCTCCGCGCATGCCTCTAGCATGAAGTCAGACAA |
| >GS_H1a (*Halluxium*) |
| AACCAATGGCCTCCTGAACGTGCGTTGCACTCTCGGGATTTCCTGAGAGTATGTCTGCTT |
| CAGTGCTTAGCTTACCCAACTGTGCAATCAGGCTGACTACCAAGCCTGCTTTTTGCGTTC |
| CTATGAGCTATTGCGCTTCCTTTGCCAATGGCTTGTTGATTGGTAGGTTCCTGCAAAATG |
| CTTTGCGCGCTGTTATTCAAGTTTCGCCTGCACGGCTTTGCTTGAGTGACGCTGCTCATG |
| CGTGCAACCGCTGGGATGCGCTCCGCGCATGCCTCTAGCATGAAGTCAGACAA |
| >GS_H1b (*Halluxium*) |
| AACCAATGGCCTCCTGAACGTGCGTTGCACTCTCGGGATTTCCTGAGAGTATGTCTGCTT |
| CAGTGCTTAGCTTACCCAACTTTGCAATCAGGCTGACTACCAAGCCTGCTTTTTGCGTTC |
| CTATGAGCTATTGCACTTCCTTTGCCAATGGCTTGTTGATTGGTAGGTTCCTGCAAAATG |
| CTTTGCGCGCTGTTATTCAAGTTTCGCCTGCACGGCTTTGCTTGAGTGACGCTGCTCATG |
| CGTGCAACCGCTGGGATGCGCTCCGCGCATGCCTCTAGCATGAAGTCAGACAA |
| >GS_H2 (*Halluxium*) |
| AACCAATGGCCTCCTGAACGTGCGTTGCACTCTCGGGATTTCCTGAGAGTATGTCTGCTT |
| CAGTGCTTAGCTTACCCAACTTTGCAAGCAGATTGATTGAATGTCTGCCTTGCGTTCCTA |
| TGAGCTATTGCGCTTCCTTTGCCAATGGCTTGTTGATTGGTAGGTTCCTGCAAAATGCTT |
| TGCGCGCTGTTATTCAAGTTTCGCCTGCACGGCTTTGCTTGAGTGACGCTGCTCATGCAT |
| GCAACCGCTGGGATGCTGGCGCATGCCTCTAGCATGAAGTCAGACAA |
| >GS_H3 (*Halluxium*) |
| AACCAATGGCCTCCTGAACGTGCGTTGCACTCTCGGGATTTCCTGAGAGTATGTCTGCTT |
| CAGTGCTTAGCTTACCCAACTTTGCAAGCAGATTGAAATTTCTGTCTTGCGTTCCTATGA |
| GCTATTGCGCTCCTCGTGCCAATGGCTTGTTGATTGGCTGGTTTCTGCAAAATGCTTTGC |
| GCGCTGTTATTCAAGCTTTGCCCATGTGGCTCCGCTTGAGTGACGCTGCTCATGCATACA |
| ACCGCTGGGATGCTGGCGCATGCCTCTAGCATGAAGTCAGGCAA |
| >GS_H4 (*Halluxium*) |
| AACCAATGGCCTCCTGAACGTGCGTTGCACTCTCGGGATTTCCTGAGAGTATGTCTGCTT |
| CAGTGCTTAGCTTACCCAACTTTGCAAGCAGGTTGTCTGCCAAGCCTGCCTTTGCGTTCC |
| TATGAGCTATTGTGCTTCCTTTGCCAATGGCTTGTTGATTGGTAGGNTCCTGCAAAATGC |
| TTTGCGCGCTGTTATTCAAGTTTCGCCTGCACGGCTTTGCTTGAGTGACGCTGCTTATGC |
| GTGCGACCGCTGGGATGCTCGCCGAGCATGCCTCTAGCATGAAGTCAGACAA |
| >GS_H4a (*Halluxium*) |
| AACCAATGGCCTCCTGAACGTGCGTTGCACTCTCGGGATTTCCTGAGAGTATGTCTGCTT |
| CAGTGCTTAGCTTACCCAACTTTGCAAGCAGGTTGTCTGCCAAGCCTGCGTTTGCGTTCC |
| TATGAGCTATTGTGCTTCCTTTGCCAATGGCTTGTTGATTGGTAGGTTCCTGCAAAATGC |
| TTTGCGCGCTGTTATTCAAGTTTCGCCTGCACGGCTTTGCTTGAGTGACGCTGCTTATGC |
| GTGCGACCGCTGGGATGCTCGCCGAGCATGCCTCTAGCATGAAGTCAGACAA |
| >GS_H5 (*Halluxium*) |
| AACCAATGGCCTCCTGAACGTGCGTTGCACTCTCGGGATTTCCTGAGAGTATGTCTGCTT |
| CAGTGCTTAGCTTACCCAACTTTGCAAGCAGGCTTAAAGTCTGCGTCGCGTTCCTATGAG |
| CTATTGTGCTTCCTTTGCCAATGGCTTGTTGAGTGGTAGGTTCCTGCAAAATGCTTTGCG |
| CGCTGTTATTCAAGTTTCGCCTGCACGGCTTTGCTTGAGTGACGCTGCTCATGCATGCAA |
| CCGCTGGGGTGCGCTCTGCGCATGCCTCTAGCATGAAGTCAGACAA |
| >GS_H5a (*Halluxium*) |
| AACCAATGGCCTCCTGAACGTGCGTTGCACTCTCGGGATTTCCTGAGAGTATGTCTGCTT |
| CAGTGCTTAGCTTACCCAACTTTGCAAGCAGGCTTAAAGTCTGCGTCGCGTTCCTATGAG |
| CTATTGTGCTTCTTTTGCCAATGGCTTGTTGAGTGGTAGGTTCCTGCAAAATGCTTTGCG |
| CGCTGTTATTCAAGTTTCGCCTGCACGGCTTTGCTTGAGTGACGCTGCTCATGCATGCAA |
| CCGCTGGGGTGCGCTCTGCGCATGCCTCTAGCATGAAGTCAGACAA |
| >GS_H5b (*Halluxium*) |
| AACCAATGGCCTCCTGAACGTGCGTTGCACTCTCGGGATTTCCTGAGAGTATGTCTGCTT |
| CAGTGCTTAGCTTACCCAACTTTGCAAGCAGGCTTAAAGTCTGCGTCGCGTTCCTATGAG |
| CTATTGTGCTTCTTCCTTTGCCAATGGCTTGTTGAGTGGTAGGTTCCTGCAAAATGCTTT |
| GCGCGCTGTTATTCAAGTTTCGCCTGCACGGCTTTGCTTGAGTGACGCTGCTCATGCATG |
| CAACCGCTGGGGTGCGCTCCGCGCATGCCTCTAGCATGAAGTCAGACAA |
| >GS_H6 (*Halluxium*) |
| AACCAATGGCCTCCTGAACGTGCGTTGCACTCTCGGGATTTCCTGAGAGTATGTCTGCTT |
| CAGTGCTTAGCTTGCAGATTTCTAGTCTTGAGCCATTGTCTTCTTTGCCAATGGCTTGTT |
| GAGTGGTAGGTTCCTGCAAAATGCTTTGCGCGCTGTTATTCAAGTTTCGCCTGCACGGCT |
| TTGCTTGAGTGACGCTGCTCATGCGTGCAACCGCTGGGATGCGCACACGCGCATGCCTCT |
| AGCATGAAACAAGCGAA |
| >GS_I1 (Clade I) |
| AACCAATGGCCTCCTGAACGCTCATTGCACCCTTGGGATTTCCTGAGGGCATGTCTGCTT |
| CAGTGCTTAGCTTTTACACCTTCGTGCGGGCGCGATGTTTTCGTGTCCTGCACTCCTGCA |
| AGCCATCGCTCAGATTTGCTTCTGATGGCTTGTTGAATGATTGGCTGTTTTGCAAGCTCA |
| AGCGCTTTGTGATTCATAGCAAACCTATGGGATTCGCTTGGGTCGCGCTGCTGATGCCTA |
| CAGCCTTCAGCATGTGAAACCGCATGCATCTTAGCATGAAGTCAGACAAGAGAACCCGCT |
| GAATTTAAGCATATAAGTAA |
| >GS_I2 (Clade I) |
| AACCAATGGCCTCCTGAACGCTCATTGCACCCTTGGGATTTCCTGAGGGCATGTCTGCTT |
| CAGTGCTTAGCTTTTACACCTTCGTGCGGGCGCGATGTTTTTGTGTCCTGCACTCCTGCA |
| AGCCATCGCTCAGATTTGTTTCTGATGGCTTGTTGAATTATTAGCTGTTTTGCAAGCTCA |
| AGCGCTTTGTGATTCATAGCAAACCTACGGGATTCGCTTGGGTCGCGCTGCTGATGCCTA |
| CAGCCTTCAGCATGTGAAGCCGCATGCATCTTAGCATGAAGTCAGACAAGAGAACCCGCT |
| GAATTTAAGCATATAAGTAA |
| >GS_I3 (Clade I) |
| AACCAATGGCCTCCTGAACGCTCATTGCACCCTTGGGATTTCCTGAGGGCATGTCTGCTT |
| CAGTGCTTAGCTTTTACACCTTCCTGCAGGCGCGATGTTTTTGCGTTTTGCACTCCTGCA |
| AGCCATCGCTCAGATTTGCTTCTGATGGCTTGTTGAATTATTGGCTGTTTTGCAAGCTCA |
| AGCGCTTTGTGATTCATAGCAAACCCACGGGATTCGCTTGGGTCGCGCTGCTGATGCCTA |
| CAGCCTTCAGCATGTGAAGCCGCATGCATCTTAGCATGAAGTCAGACAAGAGAACCCGCT |
| GAATTTAAGCATATAAGTAA |
| >GS_I4 (Clade I) |
| AACCAATGGCCTCCTGAACGCTCATTGCACCCTTGGGATTTCCTGAGGGCATGTCTGCTT |
| CAGTGCTTAGCTTTTACACCTTCCTGCAGGCGCGATGTTTTTGCGTCTTGCACTCCTGCA |
| AGCCATCGCTCAGATTTGCTTCTGATGGCTTGTTGAATTATTGGCTGTTTTGCAAGCTCA |
| AGCGCTTTGTGATTCATAGCAAACCCACGGGATTCGCTTGGGTCGCGCTGCTGATGCCTA |
| CAGCCTTCAGCATGTGAAGCCGCATGCATCTTAGCATGAAGTCAGACAAGAGAACCCGCT |
| GAATTTAAGCATATAAGTAA |
