# Supplementary material for: Diversity and dynamics of multiple symbionts contribute to early development of broadcast spawning reef-building coral Dipsastraea veroni
Source: Appl Environ Microbiol. 2025 Jan 29;91(2):e02359-24. doi: 10.1128/aem.02359-24 (PMC11837535; doi:10.1128/aem.02359-24)
Supplement: Supplemental figures — Figures S1 to S3. [file aem.02359-24-s0002.pdf]

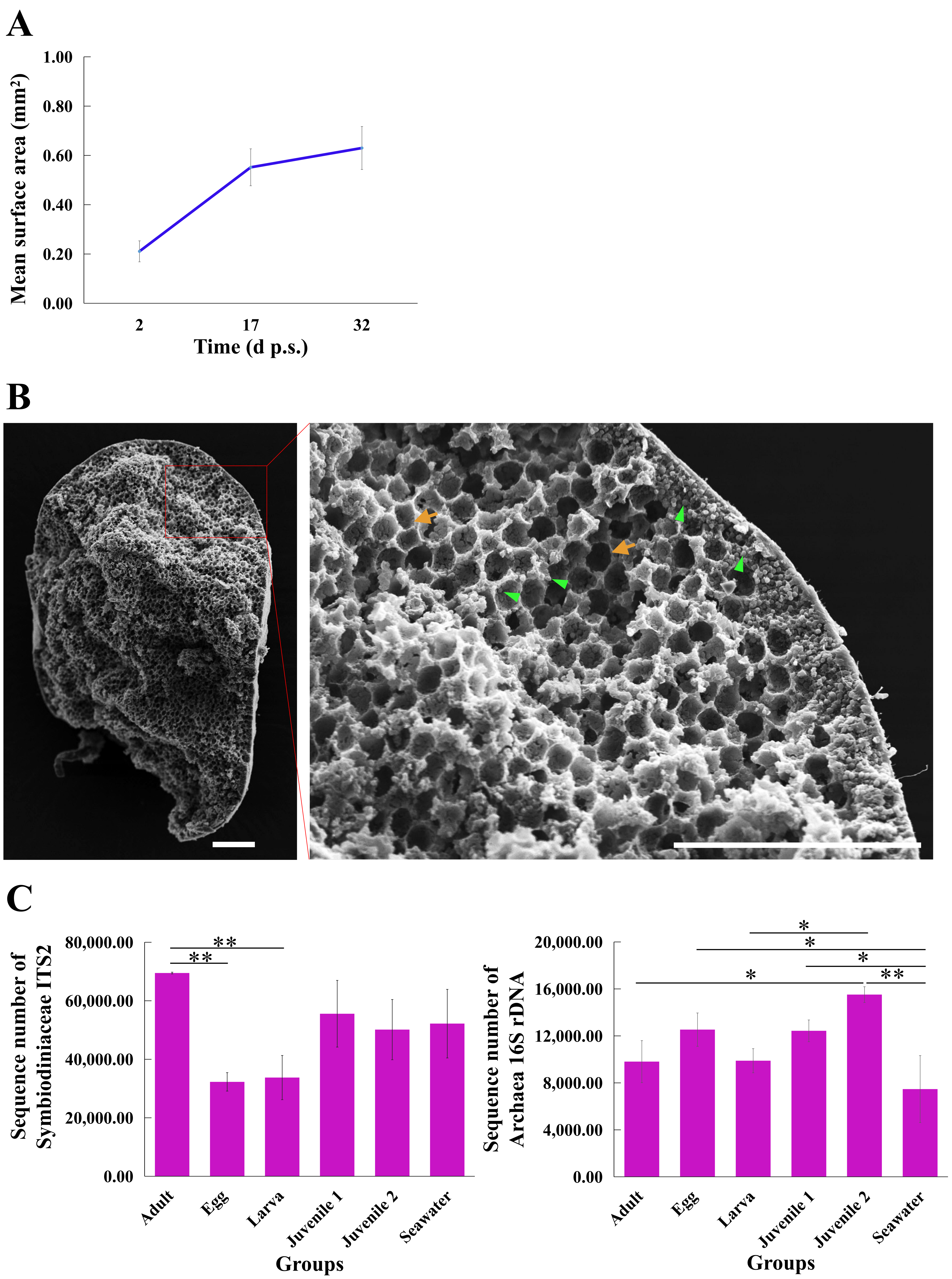

Fig. S1. Juvenile surface area (A), larval section ultrastructure (B) and sequence number of Symbiodiniaceae ITS2 and Archaea 16S rDNA (C). bar = 50 µm. Yolk bodies, green arrowheads; location of lipid granules, orange arrows.

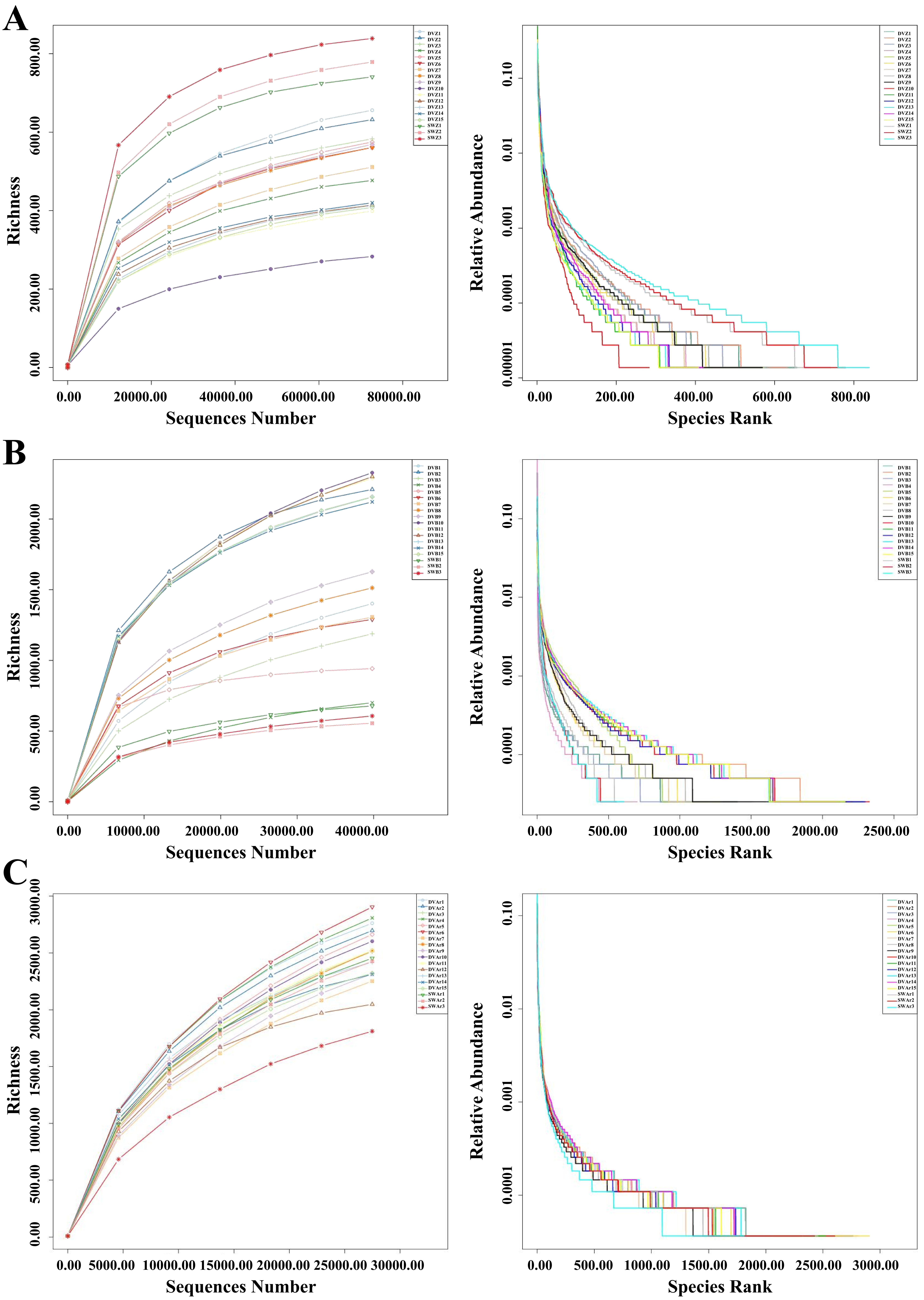

Fig. S2. Rarefaction curves and rank-abundance curves of similarity-based operational taxonomic units (OTUs) of Symbiodiniaceae ITS2(A), Bacteria 16S rDNA (B) and Archaea 16S rDNA (C) at 97% sequence identity threshold.

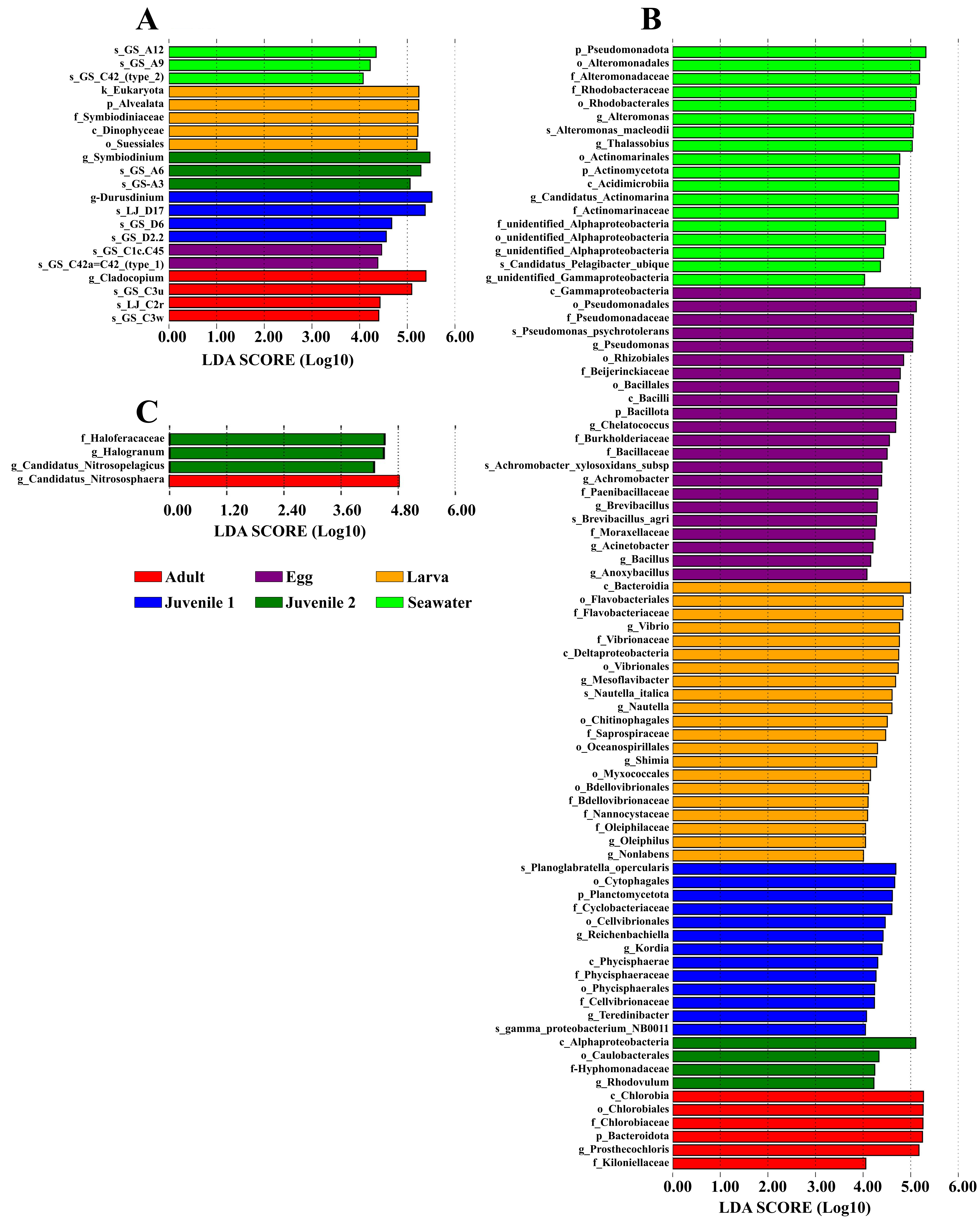

Fig. S3. LDA scores for Symbiodiniaceae (A), Bacteria (B) and Archaea (C) significantly abundant in *D.veroni* and/or seawater.

Red, purple, orange, blue, dark green and fluorescent green bars lable taxa significantly ($P < 0.05$) enriched in adult, egg, larva, juvenile 1, juvenile 2 and seawater, respectively.
